# Extraction Methods of Emerging Pollutants in Sewage Sludge: A Comprehensive Review

**DOI:** 10.3390/toxics13080661

**Published:** 2025-08-05

**Authors:** Tatiana Robledo-Mahón, Filip Mercl, Nallanthigal Sridhara Chary, Jiřina Száková, Pavel Tlustoš

**Affiliations:** Department of Agro-Environmental Chemistry and Plant Nutrition, Faculty of Agrobiology, Food and Natural Resources, Czech University of Life Sciences, Kamýcká 129, 165 00 Prague, Czech Republic; mercl@af.czu.cz (F.M.); sree1706@gmail.com (N.S.C.); szakova@af.czu.cz (J.S.); tlustos@af.czu.cz (P.T.)

**Keywords:** emerging pollutants, sewage sludge, extraction methods, clean-up, separation techniques, detection techniques

## Abstract

Sewage sludge (SS) is commonly applied as a soil amendment. This practice has raised concern about the dissemination of emerging pollutants (EPs). EPs include compounds such as flame retardants, plasticizers, pharmaceuticals, and personal care products, among others, which may pose risks to human health and ecosystems. The complexity of the SS matrix, combined to the absence of an international legislation framework, makes it necessary to evaluate the techniques available for detecting these contaminants. Detection is typically performed using sensitive analytical techniques; however, the extraction strategy selected remains a crucial step. This review aims to compile different methodologies for the determination of EPs in SS, focusing on extraction strategies reported between 2010 and 2025. Ultrasound-assisted extraction (UAE), pressurized liquid extraction (PLE), and microwave-assisted extraction (MAE) are the most widely used strategies for EPs. UAE is considered the most preferable option, as it enables the extraction of a wide range of compounds without the need for expensive equipment. Among novel techniques, the quick, easy, cheap, effective, rugged, and safe (QuEChERS) method is especially promising, as it is applicable to multiple target compounds. This review provides up-to-date information that can support the development of routine and standardized methodologies for the characterization of EPs in SS.

## 1. Introduction

Modern society uses a wide range of chemicals for industrial or domestic uses. Among these chemicals, there is a group of organic compounds known as emerging pollutants (EPs). This group includes compounds such as flame retardants (e.g., alkylphenols [APs] and organophosphate flame retardants [OPFRs]), plasticizers (e.g., bisphenol-A [BPA] and its derivatives), and pharmaceuticals and personal care products (PPCPs) (e.g., natural and synthetic hormones, UV filters, biocides, and parabens). EPs are of concern due to their potential or suspected risks to human health, primarily through their role as endocrine-disrupting compounds (EDCs) and due to their negative impacts on ecosystems [1,2,3]. For example, PPCPs and pesticides can affect endocrine systems and alter behavior in aquatic organisms. Flame retardants and plasticizers are toxic and have been linked to neurological damage in animals, as well as hormone disruption and reduced fertility in humans [4]. EPs are defined as pollutants that are not included in routine monitoring programs at the European level but may be considered for future regulation, depending on their (eco)toxicity, health effects, public perception, and monitoring data on their occurrence in the environment [5].

Many of these compounds (such as flame retardants, antidepressants, or anti-inflammatory drugs, among others) may be bioactive and bioaccumulative, exhibiting widespread occurrence and persistence. A large proportion of these substances are eventually entering into wastewater treatment plants as a consequence of household and industrial discharges. Their concentrations in the environment vary from ng to µg per liter in water bodies, and per kg in dry matrices such as SS and soil. However, conventional wastewater treatments are not specifically designed to remove these compounds, and many are retained and accumulated in the sludge after treatment. In fact, SS constitutes the final sink for most organic contaminants present in wastewater due to its specific physicochemical properties. SS, which consists of a matrix of extracellular polymers, proteins, and polysaccharides, behaves like a sponge, grinding organic compounds and heavy metals. The occurrence of EPs can vary depending on the location, the type of sludge (primary or secondary), and the physicochemical properties of the sludge. The sludge’s composition—such as its organic matter content, porosity, and surface characteristics—together with the physicochemical properties of Eps—such as their hydrophobicity, molecular weight, acid dissociation constant (pKa), octanol–water partition coefficient (Kow), solubility, and biodegradability—affect their sorption capacity [6,7,8]. For instance, Mejías et al. [9] reported that between 55 and 100% of several pharmaceutical active compounds (PhACs) can be retained in SS through sorption processes. A similar trend has been observed for flame retardants and per- and polyfluoroalkyl substances (PFAS), which are strongly sorbed onto SS due to their hydrophobic nature, often reaching high concentrations in SS [10].

The application of SS as a soil amendment is a very common practice that is gaining importance in line with the circular economy model [11]. However, nowadays, there is no consideration of EPs in these matrices, despite their potential environmental effects and, consequently, their impact on the food chain.

Therefore, the current legislation applied in the European Union countries (Directive 86/278/EEC) should be updated to align with bioeconomy and zero-waste policies, which promote the revalorization of waste [12,13]. A similar update has already occurred with wastewater legislation, which also includes methods for the detection and determination of contaminants. A full characterization of EPs in SS may be a useful tool to prevent future soil pollution and establish safe agricultural practices according to the circular economy and the EU Soil Mission. In this context, a major challenge is to develop and improve the analytical techniques for detecting EPs in solid SS samples, making them feasible tools for a legislative framework—as has already been done for water [14]. Although modern analytical instruments are highly precise, the extraction of these compounds from solid samples remains the main bottleneck.

In recent years, many studies have investigated the detection of pollutants in SS using different methodologies and targeting various compounds. Despite the large number of publications in this field, there is still a lack of unified methodologies that cover a wide range of EPs that may be potential candidates for future legislation, while also prioritizing the use of eco-friendly resources. This review addresses the diverse methodologies used to determine EPs in SS after 2010, with special emphases on extraction and validation methods. It aims to provide substantial information for selecting methodologies that are adaptable to the available resources in order to support the development of future legislation and to ensure the safe application of SS in line with circular economy principles.

## 2. Search Strategy Methodology

Articles about EPs in SS from various relevant databases (Science Direct, PubMed, Scopus, and Google Scholar) covering the period 2010–2025 were compiled, along with some reports on legislation from different websites, such as www.eur-lex.europa.eu (accessed on 23 March 2025), www.norman-network.net (accessed on 12 December 2024), and www.epa.gov (accessed on 27 November 2024). To screen the target articles, the keywords ‘sewage sludge’, ‘emerging pollutants’, ‘emerging contaminants’, and ‘extraction techniques’ were used. Articles found by hand and from the references of the initially selected articles were included. The articles were screened in order to focus on those that determined EPs in solid SS using a described validation method. By contrast, those focusing on wastewater, compost, or soil were not used. Ultimately, 161 articles were selected for the assessment. In the selected articles, an individual and detailed analysis was conducted to collect information concerning the extraction strategy used, the target compounds analyzed, the clean-up step, the detection techniques used in each case, the recovery of the method, the range of values in which the compounds were found, and the limits of detection and quantification. In addition, details regarding sample preparation and the solvents and procedures used were compiled.

## 3. Pre-Treatment of Sewage Sludge Samples

SS comprises a variety of components, including microbial aggregates, filamentous microorganisms, organic and inorganic particles, and extracellular polymeric substances, along with large amounts of water [15]. EPs represent a group of compounds with extremely wide chemical properties. EPs may have functional groups (acidic and/or basic), so they may form anionic, cationic, neutral, or zwitterionic forms. These depend on the molecules’ pKa and log Kow. Compounds with basic and hydrophobic characteristics appear to bind preferably to suspended solids [16]. Compounds with polar functional groups (carboxylic acids, aldehydes, and amines) can interact with suspended organic matter as well, resulting in adsorption to solids, despite their low lipophilicity [17,18]. Other compounds (like steroid hormones; PCPs, like triclosan; even PhACs, such as gemfibrozil, paroxetine, and mefenamic acid with Log Kow > 4) are less polar and have a moderate-to-strong affinity with suspended particulates, which finally end up in SS [19,20]. Therefore, effort should be addressed toward the extraction and separation of target compounds, the removal of particles that may interfere in further determination, or even enrichment step (which are usually the longest step); enrichment step is followed by the clean-up step, prior to the detection and quantification of the target compounds by analytical detection techniques. All these steps occur after sample collection and pre-treatment.

The analysis of SS starts with the collection of samples, where improper sample treatment can undermine the whole analysis. SS samples are usually taken directly from wastewater treatment plants (WWTPs). To ensure that samples’ conditions are not altered, they should be transported immediately at 4 °C. Samples are conventionally collected in glass amber bottles that have been washed with methanol [21,22] or in high-density polyethylene (HDPE) bottles [23]. Once they reach the laboratory, the samples may need a centrifugation or filtration process, which is especially true in the case of primary or secondary sludge that contains up to 99% water. However, it will depend on the treatment the SS has previously undergone in the WWTP. Commonly, digested and dewatered sewage sludge is the type most commonly used for soil application and is of a semi-solid character with a water content of around 75% by mass. Therefore, the sludge contains both polar and non-polar contaminants, despite their partitioning between solid and water phases. The same is true for dried sludges with less than 2% water because those are dried by hot air, so the water evaporates, but water-soluble contaminants remain in the sludge.

**Figure 1:**
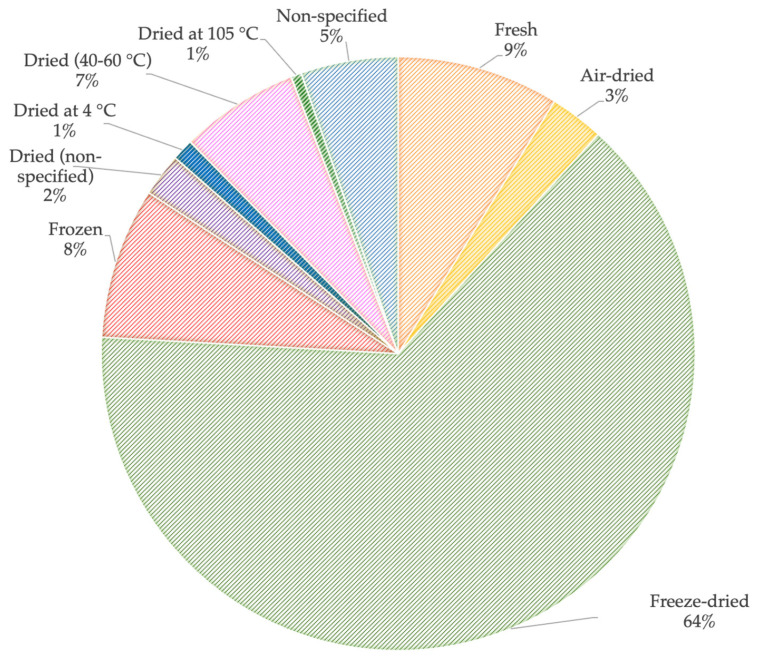
Frequency (%) of the pre-treatment used for sewage sludge samples before EP extraction, according to the articles reviewed (n = 161).

Some authors have conducted a previous treatment by adding a sodium azide solution to inhibit the biodegradation processes [24,25] or have sterilized the SS by γ-irradiation [26]. However, the most common storage technique is directly to freeze-dry the samples at a minimum temperature of −18 °C (Figure 1), followed by homogenization through grinding them with a mortar and pestle and sieving them through a 0.25 µm–2 mm mesh, based on the chosen extraction method [27,28,29,30]. Some authors, however, only froze their samples prior to analysis [31,32], while a few dried them at either 40 °C [26,33,34] or 60 °C [35,36], air-dried them at room temperature [37], or directly stored them at 4 °C before analyzing them [18,38,39]. Most previous articles have used freeze-drying, as it is a time-efficient and odor-free process. Moreover, according to some authors, it ensures zero loss of non-volatile analytes and minimizes the loss of volatile analytes [40,41]. Freeze-drying has proven to be the preferred pre-treatment and usually requires only a small amount of the sample (between 0.1–1 g). In addition, this is also the preferable pre-treatment, according to most studies, for a wide variety of compounds [42,43,44,45,46,47]. However, a good practice is to freeze-dry a larger amount of SS to ensure the representativeness of the sub-sample. Most studies have focused on optimizing the extraction; however, no comparison was found of the effect of the pre-treatment over time or of how long samples can be stored for after being freeze-dried. Therefore, research comparing the effect of pre-treatment strategies on EPs in sewage sludge is needed.

## 4. Extraction Strategies

**Figure 2:**
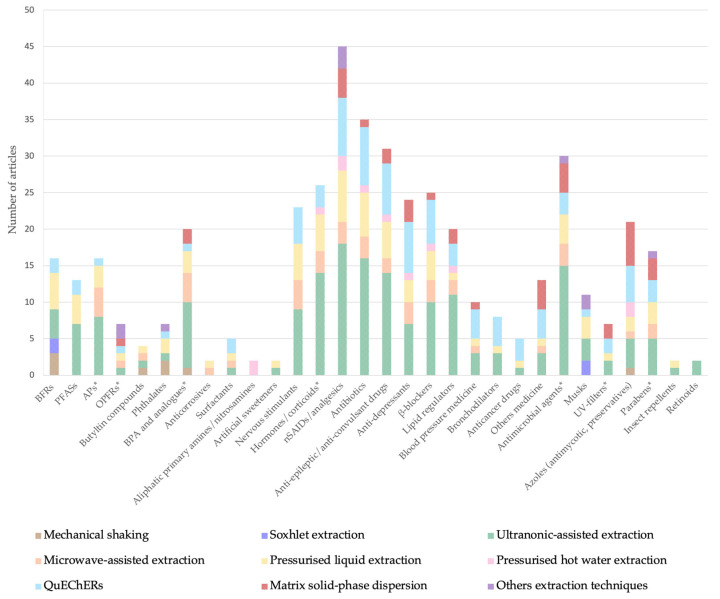
Distribution of articles included in the review according to the group of emerging compounds analyzed and the extraction strategy used. BFRs: brominated flame retardants; PFAS: per- and polyfluoroalkyl substances; APs: alkylphenol ethoxylates; OPFRs: organophosphate flame retardants; BPA: bisphenol-A; NSAIDs: nonsteroidal anti-inflammatory drugs. The “Other types of medicines” category includes histamine antagonists, anti-allergenics, anesthetics, antidiabetics, antiemetics, antiparasitics, antiarrhythmic and cardiac drugs, sympathomimetics, and sexual function agents. The “Other extraction strategies” category includes solid–liquid extraction with low-temperature purification (SLE-LTP), microwave-assisted headspace solid-phase microextraction (MA-HS-SPME), headspace solid-phase microextraction (HS-SPME), hollow-fiber liquid-phase microextraction (HF-LPME), and stir bar sorptive extraction (SBSE). * Compounds considered endocrine-disrupting compounds.

The pre-treatment and extraction of a sample can consume 70–90% of the analysis time; hence, reliable sample preparation methods, characterized by simple procedures and devices to ensure sensitive analyses, are indispensable [48]. The first step in performing determination on a solid sample is to transfer the target analytes to a liquid phase via extraction or digestion [49]. Extracting analytes from solid samples, in particular SS, is more complex than extracting from liquids, as interference between the solute and the matrix can render it difficult to completely extract pollutants. Indeed, sample extraction is a crucial step of the analytical methodology, because the presence of other compounds may cause a matrix effect (ME), such as signal enhancement or extinction, and perturb the quantification [19]. The main extraction techniques used in SS are shown in Table 1, according to the compounds targeted. Methods based on different techniques are available to extract emerging contaminants from SS. The most common approach for extracting SS samples is ultrasound-assisted extraction (UAE) (Figure 2), followed by pressurized liquid extraction (PLE); microwave-assisted extraction (MAE); matrix solid-phase dispersion (MSPD); the quick, easy, cheap, effective, rugged, and safe (QuEChERS) method; Soxhlet extraction or mechanical stirring; and solid-phase microextraction (SPME), mainly.

A clean-up step may be necessary after extraction, according to the technique used. However, this step may also be performed before extraction [50]. The solvent used can determine the efficiency of the extraction. The solvent should be chosen considering its viscosity, surface tension, and vapor pressure, and posterior filtration may be required [51]. Among the solvents frequently used, methanol is the most notable, though acetonitrile, water, hexane, dichloromethane, acetone, ethyl-acetate, and citrate buffer, as well as different mixtures of these, are all common. In the case of PPCPs, compounds’ hydrophobicity is related to their solubility in the solvent mixture and is directly linked with the relative proportion of the solvents in the mixture [31]. Some authors consider a mixture of solvents to be more effective than a single solvent for PPCPs in a solid matrix [52]. This improvement in the extraction can also owe to differences in the compounds’ polarity, as using a mixture of solvents increases the range of compounds extracted. For instance, a mixture of methanol, formic acid, and acetonitrile has been used for azole determination by MSPD [53]; furthermore, a mixture of n-hexane, dichloromethane, and acetone has been used to determine flame retardants via UAE extraction [21]. The most used solvent was methanol, which appears as a unique solvent or in mixtures with other solvents in almost half of the revised articles (63). Moreover, it has been applied for the extraction of compounds like flame retardants [54] (in combination with dichloromethane); plasticizers [55,56]; azoles and biocides (in a mixture with formic acid) [17,57]; and many PhACs, like progestogens [25], nonsteroidal anti-inflammatory drugs (NSAIDs), lipid regulators, and antibiotics [58]—mainly in UAE and MAE. However, it was not very common in mechanical shaking or Soxhlet extractions.

**Table 1:** Comparative table of the main extraction methodologies used in sewage sludge analyses.

Extraction Strategy	Cost	Solvent Consumption (mL)	Recoveries > 70%	Other General Information
Mechanical stirring	Low	10–80	UV filters, phthalates, PBDEs,	Filtration is not necessary [51,59]. Low-cost and basic equipment [51].
Soxhlet/Soxtec	Low	6	Flame retardants	Soxtec was approved by EPA as standard method [48].
UAE	Low	4–35	PFAS, PhACs, and EDCs, azoles, steroids, biocides, antibiotics, BDEs, NSAIDs, nonylphenols, PBDEs, and HBCD,	UAE using acetonitrile is valid as per EPA [59].
MAE	Low	5–30	NSAIDs, antibiotics, EDCs, PCPs, nonylphenols,	Filtration required/clean-up required [51].
PLE	High	4–50	β-blockers, estrogens, PFCs, BPA, carbamazepine, antibiotics, anticancer drugs, sedative-hypnotics, BDEs, TCS,	Required time: 30 min/15–45 min [36,51,60].
PHWE	Low	-	-	Environmentally-friendly technique [51].
QuEChERS	Low	10–25	Azoles, musks, UV filters,	It can be online-connected with SPE [60].
MSPD	High	5–20	TCS, UV filters, personal care products, azoles, cardiac drugs, NSAIDs,	No clean-up is necessary after extraction or depending on the target analyte [48,51].
SPME	High	0.5–20		Used as clean-up step [61,62].
SLE-LTP	High	4–8	PCBs and phthalates	

UAE: ultrasound-assisted extraction; MAE: microwave-assisted extraction; PLE: pressurized liquid extraction; PHWE: pressurized hot water extraction; QuEChERS: quick, easy, cheap, effective, rugged, and safe; MSPD: matrix solid-phase dispersion; SPME: solid-phase microextraction; SPE-LTP: Solid-liquid extraction with low-temperature purification; PBDEs: polybrominated diphenyl ethers; PFAS: per- and polyfluoroalkyl substances; PhACs: pharmaceutical active compounds; EDCs: endocrine-disrupting compounds; NSAIDs: nonsteroidal anti-inflammatory drugs; PBDEs: polybrominated diphenyl ethers; HBCDs, hexabromocyclododecanes; PCPs: personal care products; PFCs: perfluorinated compounds; BPA: bisphenol-A; BDEs: brominated diphenyl ethers; TCS: triclosan; PCBs: polychlorinated biphenyls.

### 4.1. Mechanical Shaking

Mechanical shaking is the most conventional and simple extraction method, which consists of an agitation procedure through the addition of a solvent and the mechanical shaking of solid samples. Agitation can be based on hand agitation or a vortex or orbital shaker, the latter being the most common, as agitation usually requires a long shaking time [60]. This method has been used for the extraction of UV filters, butyltin compounds, plasticizers, and flame retardants, which are usually treated afterwards by a clean-up column. As a traditional method, it requires a larger amount of sample and solvent compared to novel techniques in some protocols. A large amount of sample, up to 5 g (Table S2), was required in the methodology reported by Gao et al. [63] and Gani et al. [64] to determine phthalates in SS. Furthermore, it may require a higher volume of solvent, like in the methodology described by Gani et al. [64,65], where up to 80 mL (Table S2) was required to obtain results, and the method had a range of recovery results of up to 70% (Table 2). This technique has some limitations due to difficulties associated with the nature of SS; hence, in the last few years, it has been replaced by more-complex extraction techniques in order to obtain a greater range of recoveries.

### 4.2. Soxhlet Extraction

Soxhlet is another of the oldest techniques employed for solid sample preparation. It is used to separate compounds of interest from insoluble, high-molecular-weight fractions and to remove other compounds that could interfere in subsequent steps of the analysis. The most appropriate names for it are ‘leaching’ and ‘lixiviation’ [49]. In this extraction method, it is necessary to select organic solvents based on the polarities of analytes of interest; the volume of solvent will be dependent on the amount of the sample. Previous reviews on this topic have described that when Soxhlet extraction is performed on sample masses of 10–30 g, the solvent can typically go up to 300–500 mL, and extraction times range between 6 and 48 h [51,69]. For this reason, this method has been considered incompatible with the green chemistry concept due to the high solvent volume and the time and energy necessary for extraction in the past. However, in recent studies using this extraction technique (Table 3), the solvent consumption was not as high as had previously been described (Table S3). The longest extraction period was 72 h, and it was for polycyclic musks [70]. Advantages of this type of extraction include the fact that the equipment costs little, is easy to find in common laboratories, and allows parallel extractions to be performed simultaneously. In addition, some authors have noted that a filter step is not required [51,59], although others have suggested that further clean-up is necessary after Soxhlet extraction in order to remove residual water and polar impurities, fats, or sulfur [15,54] (Table S3). Nevertheless, it may be a good option for extracting polybrominated diphenyl ethers (PBDEs) due to the fact that it has the simplest procedure, requires minimal sample preparation, and recoveries are higher than 89% [54]. Moreover, in the last 15 years, some extraction methods coupled with Soxhlet have been developed, like ultrasound-assisted or microwave-assisted Soxhlet extraction [49]. An improved version, called Soxtec extraction, also exists, in which a boiling solvent is used to obtain rapid contact between the solvent and the analyte, speeding up the extraction. Articles published before 2010 have reported its application for EPs such as UV filters, polycyclic musks, and hormones, such as steroid estrogens [48]. In the last decade, the application of Soxhlet was used to determine mainly PBDEs and musks (Table 3).

### 4.3. Ultrasound-Assisted Extraction (UAE)

UAE represents an alternative to the Soxhlet method and has been broadly used for the extraction of inorganic and organic compounds. The application of ultrasound waves in a liquid medium results in a cavitation process that forms bubbles; these bubble implode when ultrasound waves continue to be applied, causing the mechanical erosion of solids and the rupturing of particles [51,60]. The selection of the solvent will depend on the polarity of the target compound. For instance, methanol has been used for a wide variety of compounds as a unique solvent or mixed with dichloromethane or acetic acid for flame retardants. In addition, methanol can be mixed with sodium hydroxide (for PFAS), formic acid (for azoles), acetone, or water (for a maximum of 16 compounds) (Table S4). Other important parameters are the ultrasound frequency, sonication time, and sample homogenization, which mainly depend on the homogeneity of the sample matrix [59]. The most popular use of ultrasound energy is an ultrasound bath, although an ultrasound probe device can be used instead; in which case, the process is alternatively known as focused ultrasound-assisted liquid extraction (FUSLE). The use of a powerful cylindrical probe that applies the ultrasound energy uniformly on the sample zone improves the cavitation effect in the liquid. The choice of one or another ultrasound extraction technique depends on the specific analysis, the time of the extraction, and the number of samples; FUSLE is considered more effective than UAE for processing large numbers [74,75]. Over the last decade, UAE has increased in importance due to the fact that it constitutes an environmentally friendly alternative to traditional techniques, can be used with any solvent, and involves less solvent consumption (2–35 mL) and a shorter extraction process (10–45 min) (Table S4) [59,76]. However, for determining hexabromocyclododecanes (HBCD) and tetrabromobisphenol (TBBPA), a longer sonication time is required [77]. In recent studies, UAE has been more commonly used than other options, like PLE and MAE (Figure 2). This is probably due to the fact that it allows the extraction of a wide variety of compounds independently of their polarity as cavitation disrupts the SS matrix, allowing the release of the analytes, and improves the efficiency of the extraction [78]. For instance, Gago-Ferrero et al. [79] determined 148 compounds (Table 4), finding that the range of recoveries was between 50 and 110% for more than 77% of the compounds. Recoveries in the range of 70–130% were found for nonsteroidal anti-inflammatory drugs (NSAIDs) [58,80] and in the case of antifungal azoles compounds or biocides [81,82] using this extraction technique. Martinez-Tena et al. [83] used FUSLE for perfluorinated compounds (PFCs), with a successful range of recovery of 69–104% [84]. In this case, the conditions of the extraction were two cycles with 65% ultrasonic irradiation power for 20 s using acetonitrile (Table S4). UAE has mostly been used to determine pharmaceutical compounds and personal care products, but it can also determine PFAS, alkylphenols, and PBDEs [21,33,85,86].

### 4.4. Microwave-Assisted Extraction (MAE)

MAE has grown in popularity as an attractive extraction technique as it allows the rapid extraction of analytes from solid matrices, heating the solvent with microwave energy. Its reduced extraction time—coupled with its improved extraction yield, better accuracy, and precision upon automation—renders MAE a preferable choice. In MAE, the sample and the extraction solvent are heated with microwave energy, so the boiling temperature is reached very quickly, thus accelerating the extraction process [15]. MAE extraction can be performed under controlled temperatures and pressures (closed system), or at atmospheric pressure (open system). The ability of the solvent used for MAE is defined by its dielectric constant, which is high for polar solvents. Alternatively, a little water may be added to a non-polar solvent, or a polar solvent may be mixed with a non-polar solvent, to increase the penetration of the microwave energy [48]. As Table 5 shows, this technique has been widely used for EDCs, mainly through the use of methanol as a solvent, thereby extracting up to 63 compounds [124,125,126,127]. Furthermore, it has been applied to the extraction of acidic drugs, linear alkylbenzene sulphonate (LAS), BPA, and phenols [18,24,128,129]. The range of the power is approximately 300–1200 W, the temperature range is between 60 and 110 °C, and the longest extraction time is 30 min (Table S5). Moreover, there may be variation when using micellar media as an extractant agent, for instance, cationic surfactants (HTAB), as used by Montesdeoca-Esponda et al. [130] for the extraction of fluoroquinolones. The final sample received after MAE is a solution, which typically needs to be cleaned up before proceeding to instrumental detection. According to the above-mentioned articles, SPE with diverse cartridges, such as Oasis HLB, C18, EnviCarb, and Florisil, tends to be used for clean-up. This extraction technique has been used for the extraction of a wide range of compounds that includes phenols, hormones, butynyl compounds, BPA, LAS, NSAIDs, antibiotics, and illicit drugs. In fact, Petrie et al. [126] determined up to 90 compounds with MAE, but some compounds showed low recoveries.

### 4.5. Pressurized Liquid Extraction (PLE)

PLE is also popularly known as high-pressure solvent extraction (HSPE), enhanced solvent extraction (ESE), or accelerated solvent extraction (ASE). This extraction technique enables the solvent to reach temperatures closer to the boiling point of the extractant, and it keeps the liquid state by increasing the pressure, thereby increasing its solubility and decreasing its viscosity and, as a result, permitting better penetration and obtaining higher extraction rates [15]. This extraction technique is the second-most-widely used after UAE for EP extraction in recent studies (Figure 2). Studies using PLE are summarized in Table 6. Several articles have presented this extraction for different EPs, especially PhACs [29,52,58,138,139,140,141,142,143,144,145,146], but also steroids, flame retardants, illicit drugs, PCPs (musk fragrances, triclosan [TCS], and derivates), EDCs (including several types of compounds from different categories), and PAHs and sweeteners [17,19,28,40,50,77,139,147,148,149,150,151]. Each extraction cycle is divided into two steps: a preheating period and static extraction or static time. The number of cycles can vary, but the maximum is four, for the determination of anticancer drugs [146]. The pressure is usually 1000 psi, and the temperature varies from 40 to 140 °C. The average volume consumption is around 15–40 mL and takes approximately 15–45 min per sample [51] (Table S6). In recent studies, the main solvent used has been a mixture of water and methanol, although other solvents, like toluene for flame retardants [149] and dichloromethane for TCS compounds [151], have also been presented. Therefore, the main factors for PLE are temperature, pressure, time of extraction, and number of cycles. The temperature can be crucial to avoid the degradation of certain compounds. The high solubilization in PLE carries undesired matrix components, along with analytes, which can interfere in the determination process; furthermore, an increase in solubility may exacerbate this issue, necessitating an additional step using SPE [59] and usually also a pre-treatment to augment the contact between the analyte and the solvent. For example, the precipitation of fatty compounds can result in interference in the extract vial. Previous studies focusing on musk fragrances have been affected by this problem, solving them via a prior clean-up step. For example, in one analysis of brominated flame retardants (BFRs), the ME was higher using PLE, and better results were obtained with the use of UAE [77]. Florisil extract, diatomaceous earth, silica, and alumina have all been used as a pre-clean-up step to remove interference, allowing good recoveries [28,40,139]. PLE requires less solvent and a shorter extraction time than Soxhlet, but the equipment required is expensive. Unlike MAE, PLE allows the use of any solvent, whereas MAE is limited to solvents that can absorb microwaves. Nonetheless, it is an automatic technology, and several extractions can be performed simultaneously, allowing many samples to be processed and reducing the extraction time required. A variation of PLE called selective pressurized liquid extraction (sPLE) also exists, in which the extraction and clean-up steps are performed as one; hence, a sorbent is added to the extraction cell [40]. The results for BPA and alkylphenols have been found to be better when using sPLE compared to FUSLE [83]. Therefore, as in PLE, in sPLE, a wide range of chemicals can be extracted, and filtration and/or derivatization are necessary before detection techniques can be applied [50]. These extraction methods have also been used for a diverse number of compounds. Compared to MAE, in this case, with PLE, flame retardants have been also extracted with high recoveries.

### 4.6. Pressurized Hot Water Extraction (PHWE)

PHWE is an environmentally friendly, organic, solvent-free technique, which may be considered a variation of PLE, wherein water is the standard extraction solvent. Elevated temperatures and pressure are used so that the water can be maintained in a liquid state. Furthermore, these conditions ensure low viscosity, surface tension, and the promotion of diffusion, thereby enhancing the extraction of the analyte. It is a green extraction technique, which is cost-effective and has the capacity to handle relatively large numbers of samples. Temperature is the main factor, although the pH of the water phase, the number of cycles, and the pressure or flush volume can also interfere in the extraction process [60]. This extraction technique was initially used for the extraction of PAHs in water [162], but has since been applied to SS for the determination of PhACs and nitrosamines (Table 7). The increase in temperature has a double effect: it can increase the extraction, but other, unwanted compounds can also be extracted, and the temperature of the water can provoke the degradation of the target analytes [48]. Therefore, it is necessary to find a balance between temperature and pressure to avoid the extraction of undesirable compounds or the degradation of the target analytes. Fewer studies have used this extraction technique relative to USE, MAE, and PLE, and they are all are focused on PhACs [29,34,163]. The solvent used is generally water, although Saleh et al. [34] obtained better results using a 0.01M NaOH solution for NSAIDs as their pKa values (4.15–4.91) render them more soluble in aqueous solvents at a basic pH by deprotonation. Two other studies have used PHWE for aliphatic primary amine and nitrosamine extraction [27,62]. Like with PLE, a pre-clean-up step can be included, such as the addition of diatomaceous earth for azoles before extraction. A post-clean-up SPE step may also take place, using a tandem of an Oasis HLB cartridge plus a lab-made Florisil cartridge [164] or by adding silicon carbide in the extraction of PhACs (Table S7). Generally, the clean-up step prior to detection techniques consists of an SPE cartridge like Oasis, the use of three-phase HF-LPME—which can reduce the ME [34]—or an SPME cleaning step [62]. Therefore, another extraction technique is commonly used in addition to complete the cleaning process of the extract and to minimize the ME. The conditions of PHWE are very similar to the those described in the Section 4.5. In recent studies, the maximum pressure used was 1500 psi, while the temperature reached between 80 and 150 °C, with a maximum number of five cycles in the case of NSAIDs [34] (Table S7). Despite the advantage of this technology being an eco-friendly method, it has been applied only for amines and a limited number of PhACs compared to UAE, MAE, or PLE.

### 4.7. The Quick, Easy, Cheap, Effective, Rugged, and Safe (QuEChERS) Method

The QuEChERS method is an extraction and clean-up technique that was originally developed to determine pesticide residues in fruits and vegetables. However, today it is considered a novel extraction technique for Eps, and its application has been extended to different environmental samples [138]. This method is based on salting-out extraction combined with a solvent (generally acetonitrile) and a dispersive SPE (dSPE). As its name suggests, it is a rapid method that consumes a low amount of solvent and uses inexpensive equipment [165]. It can also be connected with online SPE, making it more automated [60]. This method entails two extraction steps: a salting-out liquid–liquid extraction and a matrix dispersion extraction, focused on extracting the target analytes from the matrix samples and cleaning-up by dSPE, respectively [29]. To perform the first step, the addition of an organic solvent (typically acetonitrile) is required and usually combined with magnesium sulphate or another salt. At first, this extraction technique was used to analyze pesticides in SS [166,167], but later it was used for PPCPs [29,165,168,169,170], including musks, UV filters, and surfactants [30,171] as well, as Table 8 shows. In some cases, acetonitrile has been combined with a buffer (EDTA, acetate, or citrate buffer), including for PhACs, hormones, and LAS surfactants [30,165] (Table S8). Acetonitrile has been proposed as the most efficient solvent for obtaining the maximum information from SS with QuEChERS, due to the fact that the best results have been found for this solvent in the determination of LAS surfactants [30]. The addition of a buffer can improve the extraction and avoid the degradation of the analytes. In most cases, acetonitrile is acidified or mixed with water. After the addition of the organic solvent, the samples are usually shaken, either manually or by a vortex. Subsequently, different chemicals are added to facilitate the partition, combined with shaking. In the detection of PCPs performed by Ramos et al. [171], an extra ultrasonication step was included for 15 min. Finally, magnesium sulphate with sodium chloride was added, and the mixture was centrifuged, ready for the clean-up step. The QuEChERS method routinely involves dispersive SPE as a clean-up step after extraction. The preferable sorbents for dSPE, according to recent studies, are PSA (pressure swing adsorption) and C18, which are usually used to remove polar and non-polar interferences, respectively [171]. Furthermore, the SPE approach may drastically reduce the total analysis time, because SPE can be online-coupled with LC-MS/MS analysis, allowing the automated solid-phase preconcentration and clean-up to occur as a single step [170]. Cerqueira et al. [168] have highlighted the versatility and power of this extraction method, showing good recoveries even when working with a multi-residue analysis and including compounds with different polarities (Kow: −0.02–6.2). However, Marvar et al. [121] showed higher recoveries with UAE than QuEChERS in determining the metabolites of parabens and pharmaceuticals in sludge. In this case, Ramos et al. [171] combined QuEChERS with an ultrasonication step and showed that it may be a good alternative to improve recoveries in multi-residue analysis. The QuEChERS method has been applied to a broad number of compounds, such as PhACs, PCPs, and flame retardants. In fact, the methods described by Peysson and Vulliet [165] allowed them to determine 136 compounds with variable recoveries.

### 4.8. Matrix Solid-Phase Dispersion (MSPD)

MSPD was first reported in 1989 [177] and has increased in popularity in recent years due to its suitability for complex matrices, ensuring competent analyte extraction. This technique, introduced by Barker et al. [177], represents an alternative strategy for the extraction of organic environmental pollutants from solid, semi-solid, or viscous matrices. The main difference from SPE is that disruption and dispersal onto particles (including very small particles) are simultaneous in MSPD. It is characterized by a first complete fractionation of the sample matrix components, which are ground and dispersed with the sorbent material using a dispersing agent, transferring the target analytes to a cartridge and eluting them using an appropriate solvent [15,178]. The most important variables are the type of support and the combination with the appropriate solvent. The solid support plays a key role in the extraction, with commercial supports, such as C18, silica, alumina, and Florisil, among the most widely used [32]. Therefore, the selection of the dispersant and the extracting solvent resolves the method’s selectivity and extraction efficiencies. Moreover, it permits a notable reduction in the solvent used; the required equipment is not very expensive and extraction and clean-up are performed simultaneously [179]. Given that it avoids potential interferences and obtains similar efficiencies as UAE and PLE, this is an appropriate technique for complex matrices. According to the recent literature shown in Table 9, in most cases, recoveries reached above 70%. Some authors have highlighted the advantages of this technique, which allows simultaneous extraction and clean-up through a simple procedure, reducing the solvent used, the time needed for extraction, and, consequently, the cost involved [180]. In the early 2010s, this extraction technique was employed for TCS and methyl triclosan (MTCS) determination using dichloromethane and acetonitrile, respectively [180,181] (Table S9). In general, the most-analyzed compounds using this technique were PPCPs. A few studies focused on the determination of a UV stabilizer [182] and antimycotic compounds [183], while several others performed a simultaneous analysis of different compounds belonging to this category [53,179,184,185,186], especially methanol. Others compounds, like organophosphate compounds (OPs) and pesticides, were determined using this extraction technique as well [32,187]. Most studies used the dispersant C18, although others opted for Florisil and chitin [32,179], followed by a clean-up SPE step. Even though it has reached high recovery levels, it has been applied only for few compounds in SS.

### 4.9. Other Extraction Strategies

SPME is considered a sorptive-based extraction method that removes or reduces the use of organic solvents [190,191]. Two steps can be distinguished in SPME: first, analytes from the sample are extracted into the coated fiber by direct contact; and second, the fiber with concentrated analytes is transferred to an instrument for desorption. Therefore, the material in which the extraction is performed is crucial in this technique as extraction, separation, and concentration are conducted in the same step. SPME uses a fiber coated with a polymer or a solid sorbent, which extracts the analytes from the sample by absorption or adsorption. In the extraction, the fiber coating can be immersed in the sample matrix (direct-immersion, SPME), which is suitable for the extraction of low-to-medium volatility and high-to-medium polarity. However, for analytes with high-to-medium volatility and low-to-medium polarity, the fiber coating is placed in the headspace above the sampling matrix (HS-SPME); this extraction is more appropriate for such compounds [192,193]. Due to this limitation, this technique has been reported only a few times in recent studies and mostly as a complementary step in the extraction for clean-up. Probably, due to the complexity of SS, it is necessary to follow an additional step earlier to avoid direct contact between the fiber and the SS. For instance, DI-SPME after UAE for the determination of PPCPs [61] or in the case of HS-SPME in the determination of nitrosamines [62]. Furthermore, HF-LPME has been reported in the determination of NSAIDs. López-Serna et al. [61], as previously mentioned in the UAE Section (Section 4.3), used DI-SPME to determine PCPPs in water, but in SS, these authors performed the main extraction by UAE followed by online DI-SPME on-fiber to obtain absolute recoveries of 54–105% [61]. Only three studies were found using HS-SPME for musk extraction in SS samples [28,194,195]. In general terms, this method has been used after a previous extraction for samples like SS. There are several variants, like the single drop exposed to the headspace (HS-SDME) [28] and SMPE with a polypropylene hollow fiber (PP-HF characterized by the hollow fiber’s small pore size), which is used to improve liquid-phase microextraction [196]. HF-LPME can be two- or three-phase. In three-phase HF-LPME, an organic solvent is used to immobilize the porous hollow-fiber wall [39]. HF-LPME has been found in four studies for the extraction of PhACs in SS. These authors proposed this method to avoid time-consuming steps, like lyophilization and clean-up. However, they highlighted the necessity to remove the sludge prior to the extraction and afterwards perform suspension and dilution in ultrapure water to protect the hollow fiber. This extraction strategy has also been used as a complementary step to a preconcentrated or clean-up step after PHWE with HF-LPME for the determination of industrial surfactants and flame retardants [34]. These techniques are considered environmentally friendly because they consume a low amount of solvent. Additionally, only a small volume of the sample is required, and compounds with very different physical–chemical properties can be analyzed simultaneously. Despite most of them being considered liquid extraction techniques, they can be used as an additional step in SS extraction, with the advantage that SPME can be directly automated and online-coupled with analytical instrumentation [192,197]. It presents some disadvantages, like the cost of the fiber employed, and its lifespan depends on the sample and the extraction conditions, which may increase the total cost of the process.

Stir bar sorptive extraction (SBSE) was introduced by Baltussen et al. [198] and is solvent-free. It can be considered an extraction technique deriving from the application of SPME. The use of different materials for the coated fiber, such as PDMS for SPME for non-polar analytes (log Know > 5), facilitates observation of an adsorption phenomenon produced on a conventional Teflon-coated magnetic stirring rod, which is used for sample agitation. In the extraction, a bar is introduced while the sample is stirred. The organic compounds are absorbed onto the bar. Subsequently, the bar is rinsed with deionized water and dried. After sorption, the compounds are chemically desorbed and analyzed via liquid or gas chromatography [199]. SBSE has been commercialized, for instance under the trade name Twister® (Gerstel GmbH, Mulheim a/d Ruhr, Germany) [200]. The analytes from the environmental samples are transferred by sorption onto PDMS-coated stir bars suitable for non-polar analytes. At this point, the target analytes can be recovered thermally and analyzed online. Only one study has used this extraction technique to analyze parabens, TCS, and MTCS in SS [201]. In this study, the detection limits ranged from 80 ng/kg to 1.06 µg/kg, and the recoveries ranged from 91 to 110%. Microextraction by packed sorbents (MEPS) is an advanced, online, miniaturized version of SPE that can be connected directly to GC or liquid chromatography (LC) [202]. MEPS combines several steps—sample extraction, preconcentration or enrichment, and clean-up—all performed in a single device, encompassing a syringe and an MEPS barrel insert and needle (BIN). The BIN is very similar to an SPE cartridge filled with a thermos-packed sorbent. The loaded analytes are passed through the BIN, which retains and elutes in consecutive steps [202]. The sample volume ranges from 10 to 250 µL, and the sorbent bed is integrated into a liquid handling syringe, unlike SPE columns [203]. MEPS is rapid and simple and consumes much less solvent than other extraction methods. It is suitable for small samples and can be easily online-coupled with analytical instruments without modification. Only one recent article appears to exist in which MEPS was applied as a complementary step after UAE for the extraction of brominated diphenyl ethers (BDEs), improving detection [40]. The authors obtained recoveries of 88–91% with the use of a C18 cartridge combined with a mixture of solvent acetone–water (25:75, *v*/*v*).

Supercritical fluid extraction (SFE) is a single-extraction method that has been used since 1980. It entails the use of supercritical solvent conditions, enabling the solubilization of analytes via penetration into the solid matrix due to its gas-like diffusion properties and absence of surface tension [15,164]. Carbon dioxide, which is widely accepted as a friendly and completely recyclable solvent, is typically used for this purpose because of its rational critical temperatures and pressures. Between 40–150 °C and 150–450 °C, co-solvents like methanol are habitually used to enhance the solvation process of polar compounds. It is necessary to have approximately 1–5 g of solvent with a 5–20 mL volume and a process time of 10–60 min [59]. Compared to Soxtec, there is a reduction in the time needed for solvent consumption and extraction, although to date, few studies have used this technique to determine EPs in SS samples [48].

Solid–liquid extraction with low-temperature purification (SLE-LTP) is based on adding to the sample a homogeneous mixture composed of water and an organic solvent, after which the mixture is homogenized and cooled to −18 or −20 °C to freeze the matrix components and the aqueous phase [204]. This process can take 1–1.5 h (Table S10). At these temperatures, the organic phase—typically acetonitrile but alternatively a mixture of acetonitrile, ethyl acetate, and isopropanol or isopropanol and ethyl acetate—is used. The solvent remains liquid and extracts the analyte compounds of interest [205]. It has been used in the determination of flame retardants and phthalates (Table 10). Maia et al. [204] used sonication for 15 min before freezing their sample. All three of these studies showed a good range of recoveries: 66–119% (Table 10) [204,205,206]. The main advantage of this technique is that it is fast as the sample and the analysis can be performed within 2 h; moreover, the recoveries obtained show a good range according to that established (70–100%) by the United States Environmental Protection Agency (US EPA) [207].

## 5. Clean-Up

The clean-up step is focused on the removal of certain compounds that can interfere in the determination, cleaning, and concentration of the samples. Some examples of the clean-up step have been commented on in the previous section at the same time each extraction method has been described. However, the importance of this step in the extraction procedure is critical. Most extraction techniques from solid samples are unselective and require a clean-up step to select the target analytes. This step is focused on the reduction of interferences, for instance sulfuric acid and acid-activated copper granules are used to remove fat compounds and sulfur compounds, respectively, followed by passage through an SPE column in Soxhlet extraction [209]. The most common method used for the clean-up step is SPE (Table 2, Table 3, Table 4, Table 5, Table 6, Table 7, Table 8, Table 9 and Table 10). Despite SPE being an extraction method, it is also conventionally used as a clean-up procedure to complement the extraction step for environmental samples [124]. This extraction technique has been used in chemistry since 1970 and involves dispersing the analytes from a liquid to a solid state, which in this case is an adsorbent. The absorbent material can be silica-based, carbon-based, clay-based, or resins. The extraction technique allows the adsorption and purification of the analyte in the adsorbent [210]. There are three classes of sorbents available, commercialized in either cartridge or column format: adsorption (normal or reversed), ion exchange (cation or anion), and mixed-mode (combining both). Among them, adsorption materials are widely used and, in particular, the reversed-phase mode is commonly used for solid samples. The interaction of non-polar groups with non-polar functional groups on the sorbent allows the retention of the target analytes, facilitated by highly polar solvents [124]. Reversed-phase sorbents include silica-based C18, Oasis HLB (polymeric phases), Oasis MAX, and Oasis MCX. Oasis® HLB is one of the most widely used sorbents for the SS extraction of PPCPs following different extraction strategies, such as mechanical stirring [66], UAE [20,57,81,97,100,105,108,109,120], MAE [124,129], PLE [19,52,140], and PHWE [163,164]. SPE Oasis HLB® and C18 are the most used sorbents for cleaning up in UAE (Table 4). They contain a resin made of a divinylbenzene and N-vinylpyrrolidone copolymer, which enhances the retention of polar analytes [60]. Oasis MCX is another type or cartridge that consists of a cation exchanger and reversed-phase absorption, enabling it to adsorb neutral, polar, non-polar, and cationic compounds [59]. It has been used in the determination of PPCPs after MAE [126] and PLE [145,146]. Different types of columns, like reversed-phase C18 cartridges, Florisil, silica, and alumina, have been used in previous studies (Table 2, Table 3, Table 4, Table 5, Table 6, Table 7, Table 8, Table 9 and Table 10) for the determination of EDCs [17], musks [28], and flame retardants [40,85], among others. Several studies have used SPE after another extraction technique to remove interferences, such as lipids and lipophilic compounds, from organic solvents [51]. Moreover, SPE is usually coupled with the analytical instrument, usually LC-MS/MS. This strategy allows the time needed for the sample process to be reduced.

Other extraction methods have also been used as a clean-up step, including QuEChERS [31], 1,1,1-Tris(4-hydroxyphenyl)ethane, molecularly imprinted solid-phase extraction (THPE-DMISPE) [117], and salt-assisted liquid–liquid extraction (SALLE) [211]. On the other hand, some authors prefer to avoid a clean-up step, owing to its tendency to either over-estimate the target compounds due to matrix co-extracted compounds, or to underestimate them due to ionization suppression, which can affect the concentration using GC or LC with or without mass-spectrometric detection [86].

## 6. Separation and Detection Techniques

To achieve a sensitive and selective determination, a competent analytical and separation technique is crucial after the extraction and the enrichment of the target analytes. The selection of the technique is based on the physicochemical properties of the analytes to be detected. The prevailing analytical methods for the separation and detection of EPs in sludge are GC and LC. Today, these are typically applied to detect and quantify EPs with different mass analyzers, coupled with MS or MS/MS. In general, polar and less-volatile analytes are separated by LC, whereas GC is often applied for volatile and thermally stable analytes. The most common techniques engaged in the determination and quantification of EPs are presented in Table 2, Table 3, Table 4, Table 5, Table 6, Table 7, Table 8, Table 9 and Table 10.

### 6.1. Gas Chromatography

For the detection of volatiles and semi-volatiles in SS, GC is a sensible and recurrently used technique. GC in combination with an electron impact ionization (EI) source and an MS detector is applicable for volatile compounds with possible no-MEs. Although some EPs can be analyzed directly by GC-MS, the majority require a derivatization reaction to modify the structure, which improves the volatility. This is the case of EDCs, whose polar nature necessitates a derivatization step to prevent the thermal decomposition of the analyte in the injector port, along with diminishing the adsorption of the analyte onto the chromatographic column [124,188]. Despite this improvement, derivatization increases the complexity of the process, the probabilities of error, and the analysis time. Nevertheless, GC-MS has been prominently used in the determination of flame retardants [21,40,50,54,71,72,85,204,206] or EPs derived from industrial applications, such as phthalates, alkylphenols, and butynyl compounds [18,26,27,62,63,64,65,86,205], as well as PCPs and PhACs [28,37,38,61,70,76,80,101,124,129,151,171,180,181,183,194,195], like musks, UV filters, acidic drugs, TCS, and MTCS (Table 2, Table 3, Table 4, Table 5, Table 6, Table 7, Table 8, Table 9 and Table 10). For some ubiquitous contaminants, such as phthalates, caffeine, or DEET, analysis using GC may be advantageous compared to the LC due to minimization of instrumental contamination. Furthermore, some pharmaceutical compounds have a low volatility, while others contain various polar groups; therefore, these all require conversion into volatile derivatives prior to GC determination [136]. Such pre-treatment also takes place in the case of nonylphenol ethoxylates (NPEOs) and phenolic endocrines, which are compounds with a semi-volatile nature [35,116]. Some authors have used different modes from the instrumental analysis. For instance, Dobor et al. [129] and Chokwe et al. [94] performed derivatization in selected ion storage (SIS) mode for acidic drugs, while Cristal and Lacorte [85] used GC-EI-MS with selected reaction monitoring (SRM) to minimize the ME for brominated flame retardants. Moreover, EI with selected ion monitoring (SIM) mode [37] was used in the detection of PPCPs, including TCS and MTCS [151,180,181], parabens [188], and hormones [136].

Silylation and acylation are the most extensively used derivatization procedures. Silylation is chiefly used to derivatize compounds with functional groups like alcohols, carboxylic acids, and amines. Diverse silylation agents have been employed for this purpose, for example N-methyl-N-tert-butyldimethylsilyltrifluoacetamid (MTBSTFA) for PPCPs among other TCS and MTCS, or parabens [37,61,151,180,181,188]. The derivatization agent Bis(trimethylsilyl)trifluoroacetamide (BSTFA) has been preferred in the determination of phenolic compounds, and its use in combination with trimethylchlorosilane (TMCS) as a catalyst in derivatized solutions may improve the sensitivity of the method [35], even when compared with MTBSTFA [116]. The same combination was used in the determination of PPCPs by Azzouz and Ballesteros [136], who found the highest derivatization yields with BSTFA and 1% TMCS. Other derivatization agents have been used with less frequency in very specific types of determination, like hexamethyldisilazane, trifluoroacetic acid, and hydroxylamine-HCl in pyridine for acidic drugs [129]; triethylamine (TEA) and heptafluorobutyric anhydride (HFBA) for alkylphenol ethoxylates and brominated flame retardants; pentafluoro benzaldehyde (PFBAY) for aliphatic primary amines; and sodium tetraethyloborate (NaBEt4) for butyltin compounds. The most prevalent ionization mode in the analysis of EPs in SS by GC is EI [63,85]. Negative chemical ionization (NCI) has been observed in analyses of alkyl, aryl, halogenated phenols [18], and flame retardants [72], among others. Meanwhile, though EI provides strong fragmentation of the analyte molecule, NCI is soft ionization and leads to less fragmentation, especially for compounds with a positive electron affinity.

Regarding sample injection, the splitless inlet mode has been more frequently used than split-mode injection. A quadrupole analyzer operating in both full scan and SIM has regularly been used to monitor target analytes at microgram- to nanogram-level concentrations [35,70,94,151,180,181,194,206]. Linear ion trap (LIT), time-of-flight (TOF), and triple-quadrupole (QqQ) mass analyzers have been applied in some studies to obtain good sensitivity, high resolution, and mass accuracy [93,182,188]. In many recent articles, an MS/MS configuration has been used to identify and quantify the desired analytes present at very low concentrations [28,40,62,171,188]. Moreover, SIS has been implemented by Dobor et al. [129] for acidic drugs. SIS mode is known to improve the sensitivity [212]. Two-dimensional GC with high-resolution mass spectrometry (2D-GC-HRMS) has been employed in the detection of PPCPs, polychlorinated biphenyls (PCBs), and phenolic compounds, resulting in quantitation limits as low as 0.02 ng/g (Table 6) [50]. Similarly, inductively coupled plasma (GC-ICP) has been applied in the detection of PDBEs and has been reported to achieve low detection limits with high precision [54].

### 6.2. Liquid Chromatography

LC-MS permits the separation and detection of a diverse number of non-volatile organic compounds, as well as those that possess extreme polarities, like acidic pharmaceuticals, thermolabile-like steroid hormones, and high-molecular-weight compounds (like antibiotics, NSAIDs, and quinolones) with relatively high sensitivity [213]. In recent studies, LC is the most prevalent separation technique for organic compounds present in SS, accounting for around 70% of the publications. Except for strong volatiles, almost all varieties of contaminants can be determined using LC. Indeed, the LC separation technique is currently unrivalled as a method for determining trace analytes in environmental matrices, such as SS. LC allows the determination of a wide number of analytes in a reduced analytical time, with no derivatization step required to provide good sensitivity and specificity. Chromatography separation has evolved from conventional LC columns to high-performance liquid chromatography (HPLC) and ultra-high-performance liquid chromatography (UHPLC). The main difference between them is the particle diameter, which is reduced from 5 to 3 µm in common HPLC columns to 2 µm or less in UHPLC [214]. Consequently, UHPLC improves the efficiency related to the peak capacity. For instance, UHPLC has been used in the determination of PhACs, such as hormones and corticoids [125]; anticancer drugs [146]; progestogens [25]; azoles [57]; PCPs, such as biocides [17] and UV filters [66]; illicit drugs [126,159]; and flame retardants [84,149] (Table 2, Table 3, Table 4, Table 5, Table 6, Table 7, Table 8, Table 9 and Table 10). HPLC is based on the reverse phase (RP-LC) and is the preferred method that uses a non-polar stationary phase (usually C18 columns). However, there are different methods available for separating polar compounds, such as hydrophilic-interaction liquid chromatography (HILIC) using a multi-model partition method and rapid-resolution liquid chromatography (RR-LC). Both have been reported less in recent studies, despite being employed. For instance, HILIC has been used to determine 27 PhACs [184], while RR-LC has been used to determine steroids and antibiotics [22,108].

Electrospray ionization (ESI) and atmospheric pressure chemical ionization (APCI) are the most commonly used ionization sources in the determination of EPs. While ESI is susceptible to background interference and signal suppression, APCI provides better ionization of non-polar analytes and limits matrix interferences [215]. APCI, rather than ESI and atmospheric pressure photo ionization (APPI), has been widely used in the detection of flame retardants, PFPAs, and perfluorooctanesulfonic acids (PFOs) [147,149]. APCI has also been employed to determine carbamazepine by laser diode thermal desorption after PLE [141], as well as PCPs and steroids after UAE [41]. Zacs and Bartkevics [95] employed a heated-ESI (HESI) interface in negative mode for the quantitation of PFAS and PFOS. Even though ESI seems to face more problems, it has been widely used for the determination of a wide spectrum of PCPs, illicit drugs, sweeteners, flame retardants, and plasticizers [34,36,39,53,79,139,144,150,169,170,179,183,184,186,187].

Similarly, GC, MS, and MS/MS are highly preferred detectors in the quantitation of EPs with LC (Table 2, Table 3, Table 4, Table 5, Table 6, Table 7, Table 8, Table 9 and Table 10). Few works using diode array detectors (DADs) and fluorescence detectors (FDs) were found in the detection of Eps, and those were mainly focused on the detection of PPCPs, including antibiotics, anti-epileptic drugs, beta-blockers, lipid regulators, hormones, parabens, and LAS [41,105,128,130,141]. MS preferences regarding the determination of EPs include the quadrupole and linear ion trap (qLIT) mode, which offers virtuous sensitivity but limited resolving power compared to higher-mass-resolution instruments like TOF and Orbitrap. Various mass analyzers arranged in a tandem configuration, such as QqQ and hybrid mass spectrometers, like quadrupole TOF (qTOF), are expected to yield accurate mass measurements that can be applied in the determination of a wide variety of EPs in complex environmental samples [214]. Recent studies include some examples of hybrid instruments, such as Orbitrap UHPLC coupled with an Orbitrap Exactive mass analyzer, which was used after PHWE, and the QuEChERS method for azoles and benzenesulfonamide derivates, obtaining recoveries above 80% in the majority of compounds [29,95,164]. Furthermore, mass analyzers arranged in a tandem configuration, such as QqQ, as well as hybrid mass spectrometers, like qQTOF, have been effectually applied in the determination of PhACs and PCPs [165,179,183,186,187]. Liquid chromatography–quadrupole linear ion trap mass spectrometry (LC-QqLIT-MS) has also been used in the determination of EDCs, PFCs, and illicit drugs [77,150,154].

MS/MS has tended to be preferred, owing to its sensitivity and selectivity, in addition to its ability to provide information regarding the target analytes (Table 2, Table 3, Table 4, Table 5, Table 6, Table 7, Table 8, Table 9 and Table 10). Moreover, the two transition ions obtained in multiple-reaction monitoring (MRM) mode are required to ensure the selectivity of the analysis. The transition fragment with the finest signal intensity is selected for quantification, and the next-best fragment is used for confirmation, making compound identification possible, even at very low concentrations [168,215]. qTOF provides high mass accuracy and resolution [165,179,183,186,187,215,216], allowing for more-sensitive limits of detection (LODs) and quantification (LOQs). For instance, Triñanes et al. [179] obtained a range of 0.005–0.05 ng/g for five different NSAIDs (Table 9).

## 7. Validation Method

SS is a challenging matrix because of its composition; moreover, the concentration of the pollutants present can vary according to the WWTP’s inputs. Although some methods can be validated by official bodies, such as the International Organization for Standardization (ISO) in Europe [154] and the US EPA [21,24,100], the majority of methods are validated ‘in-house’. Given that most guidelines are focused on food and agriculture, some samples’ characteristics will differ. Nonetheless, the International Union of Pure and Applied Chemistry (IUPAC) guidelines are suitable for environmental samples. A method’s development must be validated in compliance with the ‘in-house’ recommendations. According to the IUPAC’s guidelines, the main requirements in ‘in-house’ method validation are applicability, selectivity, calibration, accuracy, precision, range, limit of detection, quantification, and sensitivity [217]. In this respect, extraction methods focus on removing elements that may interfere with the determination of the target analytes. Due to the high complexity of this matrix, the parameters required to perform a comprehensive and feasible validation method must be consistent and coherent. Matrix interferences may pose problems in LC-MS/MS analyses, generally caused by either ion suppression or enhancement. ME can be described as the phenomenon whereby the efficiency of the ionization process in the MS source is altered, possibly affecting sensitivity and accuracy [57,218]. As a result, it can seriously affect the parameter validation method. In GC-MS, ME is generally ignored because of the highly refined sample preparation and clean-up involved, but it is not always negligible. Precision and accuracy are crucial parameters for avoiding problems deriving from ME in environmental samples like SS. ME can vary according to the composition of the particular SS. Furthermore, the complexity of a sample like SS can entail low reproducibility in the analysis [77]. In recent studies, the basic parameters in the determination of EPs in SS are selectivity, accuracy (bias), linearity (calibration model), precision, and sensitivity [219,220]; these are summarized in Table 11.

### 7.1. Selectivity

Selectivity is the degree to which a method can quantify the analyte accurately in the presence of an interference [220]. To measure the selectivity, the ME is the main parameter. There are three main techniques available for evaluating the ME—(1) the post-column infusion system, (2) ‘slope ratio analysis’, and (3) the post-extraction spike method [221]—the third of which is the most commonly used method in recent studies. The ME can be evaluated by comparing a standard prepared in pure solvent with another prepared using sample extracts. The most cited equation to calculate the ME has been described by Matuszewski et al. [222], although other authors have used different equations [57,127,218], albeit with the same principle in mind, as in the following equation [146]:

(1) $$ME\left(\%\right)=\left(1-\frac{A_{postextract}-A_{back}}{A_{spike}}-1\right)\times 100$$

where A_postextract_ is the peak area of the analyte in the extract spike just before the analysis, A_back_ is the peak area of the analyte in the extract of the native non-spiked sample (background area), and A_spike_ corresponds to the peak area of the analyte in pure solvent. The absolute and relative ME can be calculated without correcting the area of the analyte, usually based on an isotopically labelled analogue area [146]. This way to calculate the ME is based on the peak ratio. Another way to calculate ME is based on the calibration graph methods (slopes). The ME is given by comparing the slopes of the external calibration curve and the matrix-matched calibration curve [121]. In this case, two curves are prepared, one in solvent and one in the matrix-matched calibration graph. Both slopes are compared according to the same formula. The ME based on the slope allows us to evaluate ME in a wide range of concentrations and for samples that already contain analytes but assume that ME is independent of analyte concentrations. It must be stressed that each SS sample, especially SSs of different WWTP origins, is different in terms of its elemental composition, quality, and quantity of organic matter. Therefore, the quantification of ME should be done for each SS sample independently. To perform ME determination by comparing the slopes of matrix-matched and external calibrations for each SS sample independently is clearly not feasible due to enormous consumption of labor, instrumental time, and chemicals. Moreover, for most of the EP analytes, a blank matrix that is free of analytes is not available. Therefore, when analyzing the bigger set of different SS samples, the post-extraction spike method seems to be the method of choice in situations when information on the absolute ME is necessary.

To compensate the matrix effect, Cortese et al. [221] suggest two strategies, depending on whether a blank matrix is available or not. If the blank matrix is available, (1) calibrate by using the matrix-matched calibration standard; otherwise, use the (2) isotope-labelled internal standard. The use of stable isotope-labelled internal standard (SIL-IS) compounds, so called isotope dilution methods, can be used to assess recovery and to compensate for analyte losses during the sample preparation and for ME in one step [144]. When these compounds are commercially available, they may represent the most effective approach for compensating for ME. However, it is often ignored that in some cases, the retention time of SIL-IL and target compounds is not exactly the same during LC analysis, so the ME may vary slightly. SIL-IL compounds have been extensively used in many studies [22,35,98,100,106], but not all analytes have an SIL-IS readily available, or they can be very expensive [218].

Furthermore, optimization of mass spectrometry parameters and chromatographic conditions are necessary. If, despite these strategies, the ME still persists, dilution and a more exhaustive clean-up step are recommended. Clean-up optimization can be focused on protein, phospholipid, lipid, and sugar removal. ME in sludge samples tends to be more significant because of the presence of larger quantities of endogenous components, like lipids, peptides, carbohydrates, highly polar compounds, non-volatile solutes, and metabolites of other organic compounds, along with inorganic salts, like sulphates and phosphates [148]. Components of humic and fulvic acids have also been associated with significant ME [144]. In addition to considering the lipophilicity of the compounds, Triñanes et al. [179] have highlighted factors such as solubility, vapor pressure, and temperature. A remarkable ME has been described in the determination of quinolones due to the strong interaction between quinolones and organic matter, the main component of SS. Both are able to form stable complexes and can also be affected by acid–base properties, with the pH and the extractor solvent being the most inoperant variables in their quantification [36]. A clean-up step is intended to reduce the ME [22]. However, in some cases, this step can reduce the recovery of the target analyte. For instance, Benedetti et al. [173] determined lower recovery after a clean-up step in the determination of ciprofloxacin and erythromycin than when the process was performed without one (likely caused by adsorption in the clean-up phase due to affinity to the PSA sorbent), with dilution here being the best option to avoid signal suppression.

### 7.2. Accuracy and Recovery

To assess the efficiency of the extraction conditions, the samples are spiked with standard additions. Spiking allows the recovery of the target analyte to be calculated. Accuracy can be determined by comparing the concentration with the spiked samples at different concentration levels using the following equation [121]:

(2) $$Accuracy\left(\%\right)=\frac{C_{obtained}-C_{blank}}{C_{spike}}\times 100$$

where C_obtained_ is the obtained concentration, C_blank_ is the blank sample concentration, and C_spiked_ the spiked concentration. In the same study, recovery (%R) was calculated by comparing the peak areas of the analytes obtained from the spiked samples with those in matrix-matched calibration standards, as follows:

(3) $$Absolute\;recovery\left(\%\right)=\frac{A_{spiked\;sample}}{A_{matrix}-A_{matched\;calibration\;standard}}\times 100$$

Other authors have directly calculated the accuracy by recovering the target analytes; this is possible after applying a known concentration and detecting them after the method is performed, based on the concentration in question:

(4) $$Absolute\;recovery\left(\%\right)=\frac{C_{spiked\;sample}-C_{unspiked\;sample}}{C_{spike}}\times 100$$

where C_spiked_ samples and C_unspiked_ samples are the concentrations of spiked and unspiked samples, respectively, and C_spike_ is the spiking concentration [119]. Spiked and unspiked samples refer to the samples after and before spiking, respectively. When the target analyte cannot be detected, it is necessary to spike before and after, to be within the LODs. The extraction strategy’s optimization must be organized according to the target analytes, including the recovery. For example, in a case where the target compound is present in a very low amount, it is necessary to spike before and after the extraction to check the recovery. Different concentrations are commonly used and highly recommended to spike the samples in order to compare the recoveries, usually fortified with three concentration levels [136,180]. In general, compounds whose log Kow values are lower than four can be retained in the solvent phase, in contrast to non-polar organic compounds whose log Kow values are higher than four [17]. According to the US EPA, the general acceptable range of recoveries in the determination of organic contaminants is 70–130% [207]. High values of recovery have also been reported as the result of an ME in the determination of pharmaceuticals by GC-MS [80].

Many authors have determined these parameters using a spiking procedure. As has been abovementioned, this requires spiking samples, and it involves the addition of an analyte or a group of analytes in a given concentration, enabling one to determine possible interferences in the signal of the target analyte. Thorough mixing the standard solution and the SS to promote good interaction between the analyte and the matrix is a critical step of spiking [58,119] as it enables a potential partition equilibrium. Some authors stablished a period of between 2 h [79], overnight [148], and even 24 h [58] to allow a good interaction between the analyte and the sample and promote the evaporation of solvent. In other cases, samples were extracted right after analyte additions [119]. However, performing extraction immediately after spiking will likely lead to the overestimation of the method recovery. Contrarily, a long contact time may cause a degradation of the analytes and thus underestimate the recovery. To our knowledge, there is no available study comparing the contact time of the spiking solution and the matrix on the recovery of EPs from sewage sludge. Considering the factors mentioned above, we recommend keeping the samples after spiking in the fridge at 4 °C overnight for the minimization of potential microbial activity and to provide enough contact time for equilibrium establishment.

### 7.3. Linearity

To validate the linearity, an appropriate calibration set with six or more calibration standards should be performed to determine the linear equations and the coefficients of determination (R^2^). Furthermore, the matrix-matched calibration curves should show good linearity to ensure that the method is applicable across the chosen concentration range and no secondary reactions or solubility issues are present for the given analyte. However, five calibration points have also been accepted to validate the method [64,133]. For instance, Llorca et al. [154] included seven points for each analyte in their determination of PFO compounds. Malvar et al. [121] performed 12 calibration points in their validation method for parabens and pharmaceutical compounds, and Albero et al. [188] analyzed six points for parabens. Replicates in the injection of each standard solution and each stability test of the derivatized samples have also been included to correct linearity [80].

### 7.4. Precision

Precision can be determined in terms of the repeatability of the method, calculated as the relative standard deviation (%RSD). Intra- and inter-day precision can be calculated to check the repeatability of the method through comparing the same samples on the same or different days. A value lower than 10% is optimal for inter-day precision [61,77] in SS, while a slope value of <3–4% has been suggested as a guide for method applicability in biological samples [218]. For instance, Ömeroğlu et al. [116] used the method provided by the EPA [223] to calculate nonylphenols after UAE. Junior et al. [133] calculated intra-day and intermediate precision (for three days) using a coefficient of variation (CV, or repeatability, %RSD) at three different concentration levels in order to determine antidepressants and caffeine, according to the following equation:

(5) $$CV=\frac{sd}{mean}\times 100$$

High values of %RSD can be related to the pre-treatment of the samples. Indeed, Ramos et al. [171] found higher values of %RSD (>50%) in the determination of musks and UV filters by GC-MS/MS. This was probably due to the water content and incomplete sample homogenization, but when the samples were centrifuged, lyophilized, and homogenized by mortar and pestle, they obtained lower values of %RSD (<5%).

### 7.5. Sensitivity

LODs and LOQs, which correspond to the lowest concentration at which each compound can be detected and quantified, respectively, are required to determine a method’s sensitivity. LOQs are defined as the concentration of each compound that provides a signal 10 times higher than the average baseline noise [181] and are determined using the standard error of the intercept and the slope of the calibration curve, as proposed by the International Conference on Harmonization of Technical Requirements for Registration of Pharmaceuticals for Human Use [41] and the ISO [224]. LODs and LOQs are typically estimated based on a signal-to-noise ratio of 3:1 and 10:1, respectively [119,121]:

(6) $$LOD=\frac{{3S}_{a}}{b}$$

(7) $$LOQ=\frac{{10S}_{a}}{b}$$

where S_a_ is the standard deviation of the intercept of the calibration curve in the presence of the matrix, and b is the angular coefficient. Gorga et al. [148] calculated the method limits of quantification (MLOQs) as the lowest concentration fulfilling all four of the following criteria: (1) a calibration curve with a bias of <1.5%, (2) %RSS < 19%, (3) acceptable peak shapes, and (4) a signal-to-noise ratio of >10. Other authors have reported a different way of estimating the LOQs—specifically, based on the instrumental quantification limit (IQL)—according to the following equation [119]:

(8) $$LOQ=\frac{IQL\times V\times 100}{Recovery\times W}$$

where IQL is defined as the lowest concentration in pure water with a signal-to-noise ratio (S/N) of 10 for each compound, V is the sample volume, and W is the sample weight. Huang et al. [57] have also used the IQL to calculate the method quantification limit (MQL), as follows:

(9) $$MQL\left(µg/{kg}_{DM}\right)=\left(\frac{IL-V_{extract}}{MEff_{abs}-m}\right)\times 100$$

where IL is the instrumental limit, V_extract_ is the volume of the final extract, MEff_abs_ is the efficiency of the method, and m is the weight of the dry samples [146]. For instance, Scheurer et al. [145] calculated the LOQ by spiking eight samples of sludge with the target analyte and subtracting the background concentration. The standard deviation and the mean measure were calculated; furthermore, the LOD was computed as three times the SD and the LOQ as 10 times the standard deviation [145].

## 8. Conclusions and Future Perspectives

SSs exhibit significant variability in composition, concentration, and the range of organic pollutants present. The inherent complexity and heterogeneity of SS requires careful sample preparation—including extraction and clean-up steps—to isolate and identify EPs accurately.

Among sample pre-treatment strategies, freeze-drying remains the most effective approach. The extraction step is the bottleneck of the procedure. There has been a noteworthy rise in the number of publications pertaining to the determination of EPs in SS, wherein UAE, PLE, and MAE are the most commonly used techniques for SS extraction. In addition, new techniques with low levels of solvent (or none at all) have been reported in appreciable numbers, including QuEChERS, PHWE, and MSPD. Among these, the QuEChERS method appears to be a promising and adaptable strategy for SS, though it remains expensive and laborious. However, UAE seems to be an optimal option since it allows for the determination of a wide range of compounds in a single extraction step. It also provides good recoveries and requires only low-cost equipment, with moderate solvent use and a small sample amount. The clean-up step is usually performed by SPE, but it raises the price and time of the procedure. However, recent improvements in the sensitivity of mass detectors now allow omitting the clean-up, which can be replaced by simple dilution of the sample matrix.

In terms of distribution, the main groups of Eps, like flame retardants, PPCPs, and plasticizers, among others, have been widely studied and detected in SS. Future research should focus on the transformation products formed in the partial degradation of the EPs, as well as enantiomeric distribution, which can affect the pollutants’ persistence in the environment. The enantiomeric composition of chiral contaminants may provide relevant insights into the potential toxic effects of these compounds, yet very few studies have addressed this in the context of SS.

Currently, the general trend is the target screening of the EPs. However, the same extraction techniques can be adapted for the identification of compounds using non-target screening approaches. Non-target screening is currently being applied to less-complex matrices—especially water—and is expected to become a conventional practice in the coming years, even for more complex samples, such as soils or SS. This approach will identify new compounds and transformation products, but it will also require significant optimization of the extraction and clean-up strategies. This will probably be one of the major challenges in the field over the next decade, especially with the implementation of the most novel detection instruments.

Scientific efforts should focus on improving the extraction step to allow detection of a larger number of compounds in a single extraction and on standardizing a protocol for SS, including the development of precise and affordable methods suitable for incorporation into legislation.

## Figures and Tables

**Table 2:** Determination of EPs in sewage sludge, based on mechanical shaking.

Analytes	Extraction Solvent	Clean-Up	Detection Technique	R (%)	Range of Concentration	LOD	LOQ	Ref.
Flame retardants (6 PBDEs)	0.1 M HCl in MeOH, Tris-citrate buffer pH: 6 (50 mL) and iso-octane (2 mL) were used as co-extractors		GC-ICP-MS	95–104	<0.209–66.6 ng/g	0.302–0.182 ng/g	0.649–1.01 ng/g	[54]
PCPs (5 benzophenones—type UV; 2 benzotriazoles)	Ethyl acetate-DCM (1:1, *v*/*v*)	SPE (Oasis HLB^®^)	LC-MS/MS, GC-MS	70–116	nd, 0.730–198 ng/g	<LOQ–5920 ng/g	0.1–1.65 ng/g	[66]
Plasticizers (2 butyltin compounds)	Acetic acid		Derivatization with NaBEt_4_+ GC-ICP-MS	-	534–1569 ng Sn/g	-	-	[26] ^a^
Plasticizers (6 phthalates)	n-hexane	Clean up column	GC-MS	86–114	nd, 126.18–9408.49 ng/g	0.051–0.13 ng/g	-	[63]
Plasticizers (4 phthalates)	n-hexane	Clean up column	GC-MS	80–95	0.1–38 mg/kg	0.071–0.216 μg/L	0.182–0.342 μg/L	[64]
Plasticizers (DEHP)	*n*-hexane-DCM (3:1, *v*/*v*)	Clean up column	GC-MS	-	0–10,000 ng/g	-	-	[65]
Plasticizers (4 phthalates)	*n*-hexane-DCM (3:1, *v*/*v*)	10 g of alumina	GC-MS	80.01–95.20	nd, 0.13–10.21 mg/kg	0.071–0.216 μg/L	0.182–0.342 μg/L	[67]
Plasticizers (BPA and analogues	Methyl tertiary butyl ether		UPLC-MS	>82.0	<LOD. 0.1–378.5 ng/g	10.0–6453.3 ng/g	10.0–6453.3 ng/g	[68]

R: recovery; LOD: limit of detection; LOQ: limit of quantification; Ref: reference; PBDEs: polybrominated diphenyl ethers; DEHP: Bis(2-ethylhexyl) phthalate; DCM: dichloromethane; SPE: solid-phase extraction; HLB: hydrophilic–lipophilic balance.; GC-MS-: gas chromatography; LC-MS/MS: liquid chromatography–tandem mass spectrometry; GC-ICP-MS: gas chromatography–inductively coupled plasma–mass spectrometry. ^a^ Different extraction methods were compared, and there were no significant differences between them.

**Table 3:** Determination of EPs in sewage sludge, based on Soxhlet extraction.

Analytes	Extraction Solvent	Clean-Up	Detection Technique	R (%)	Range of Concentration	LOD	LOQ	Ref.
Flame retardants (4 PBDEs)	DCM		GC-MS-NCI	89–105	1.1–400.3 ng/g		0.1–1.2 ng/g	[71]
Flame retardants (13 PBDEs)	Acetone-hexane (4:1, *v*/*v*)	Silica gel column	GC-MS-NCI	-	0.2–9410 ng/g	0.017–370 ppb	-	[72]
PCPs (6 musks)	DCM	Silica gel and alumina (2:1)	GC/MS	50.90–97.19	270.0–8421.2 ng/g	-	-	[73]
PCPs (4 polycyclic musks)	DCM	Silica gel and alumina (2:1)	GC-MS	-	enantiomeric fraction provided	0.010–0.045 μg/L	-	[70]

R: recovery; LOD: limit of detection; LOQ: limit of quantification; Ref: reference; nd: non-detected; PBDEs: polybrominated diphenyl ethers; PCPs: personal care products; DCM: dichloromethane; GC/MS-: gas chromatography–mass spectrometry; GC/MS-NCI: GC-MS–negative chemical ionization.

**Table 4:** Determination of EPs in sewage sludge, based on ultrasound-assisted extraction (UAE).

Analytes	Extraction Solvent	Clean-Up	Detection Technique	R (%)	Range of Concentration	LOD	LOQ	Ref.
EDCs (3) NSAIDs (4)			GC-MS		<LOD-6297 ng/g			[87]
EPs (119)	ACN–water (1:1, *v*/*v* 0.1% formic acid)		UPLC-MS/MS		5–17,000 ng/g ^b^	0.14–20 ng/		[88]
EPs (43)	Extractant solvent (10.5 g of citric acid and 10.2 g of magnesium chloride in 1 L of ultrapure water (pH: 4)-ACN (1:1, *v*/*v*)	SPE (Oasis HLB^®^)	UPLC-TQD	48.69–114.21	nd, 1.9–229 ng/g		MQL: 0.94–8 ng/L	[89]
EPs (178)	Hexane–DCM (1:1, *v*/*v*)		GC-MS/UPLC-ESI-MS/MS	86–119	>20–112.1 ng/g		4–20 μg/kg	[90]
EPs (68)	2% NH_4_OH in MeOH, 2% formic acid in MeOH and MeOH	SPE (Oasis HLB^®^)	UHPLC-MS/MS	0–122	nd-170 20 μg/kg	MDL: 0.025–7.4 mg/kg	MQL: 0.080–49 mg/kg,	[45]
EPs (41 illicit drugs)	McIlvain buffer–methanol (1:1, *v*/*v*)	SPE (Strata-X cartridges)	LC–MS/MS	52–197	1–171 ng/g	0.12–1.32 ng/g	0.15–3.36 ng/g	[91]
Flame retardants (HBCD and TBBPA)	DCM-MeOH (1:9, *v*/*v*)	SPE (C18)	LC-QqLIT-MS	39–120	nd-1849 ng/g		4.64–220 ng/g	[77]
Flame retardants (13 PFAS)	MeOH	EnviCarb cartridges	HPLC-MS/MS	69–141	nd, <0.01–286.81 ng/g	0.01–0.21 ng/g	0.02–0.71 ng/g	[33]
Flame retardants (OPFRs, PBDEs and NBFRs)	Ethyl acetate–cyclohexane (5:2; *v*/*v*)	Florisil^®^ cartridges	GC-EI-MS/MS	64–140	Not found	MDLs: 6.2–575 ng/g	-	[85]
Flame retardants, plasticizers, and PCPs (23 compounds)	MeOH–acetic acid (90:10 *v*/*v*)	SPE (C18)	LC-MS/MS	69–120	<MDL-365 ng/g	MDL: 0.01–6.17 ng/g	MQL: 0.04–20.6 ng/g	[55]
Flame retardants (PBDEs and HBCD)	*n*-hexane–DCM–acetone (7:7:1, *v*/*v*)	Alumina clean-up	GC coupled with micro-cell electro capture detector	80.6–100.4	<LOD-2.46–107 ng/g	0.09–3.94 ng/g	0.19–12.53 ng/g	[21] ^2^
Flame retardants (7 BDEs)	can		UPLC-MS/MS	69–104	nd, <0.18–3.03 ng/g	0.06–0.20 ng/g	0.18–0.60 ng/g	[84]
Flame retardants (7 PCBs)	Sodium acetate buffer (pH: 3.4) and hexane	SPE (Strata SI—silica)	GC-MS/MS QqQ		10.5–588 ng/g			[92]
Industrial and domestic products (11 PFAS)	THF–acetic acid (1:1, *v*/*v*) or ACN-THF (1:1, *v*/*v*)	SPE (WAX cartridge and EnviCarb cartridges)	HPLC-MS/MS	24–107	nd, <MQLs-10.7 ng/g	MQL: 0.6–5.1 ng/g		[93]
Industrial surfactants and flame retardants (APEs and BDEs)	Hexane–acetone (4:1; *v*/*v*)	Acidic silica column and Cu powder	derivatization with HFBA GC-MS	44.93–100.88	<LOD-664.46 ng/g	0.12–5 ng/g	0.72–16.40 ng/g	[94]
Industrial and domestic products (PFAS)	MeOH and 0.2 M NaOH solution	SPE (Strata-X-AW) clean-up with graphitized	HPLC-Orbitrap-MS	88–95	<RL—1.31	RL: 0.04–0.12 ng/g		[95]
Industrial and domestic products (46 PFAS)	0.1 % (*v*/*v*) ammonia in MeOH	SPE (Oasis WAX)	LC-MS/MS	76–102	nd-883 ng/g	MDL: <0.01–0.12 ng/g		[42]
PCPs (6 azoles)	MeOH–formic acid (100:0.1 *v*/*v*)	SPE (Oasis HLB^®^)	UHPLC-MS/MS	52–110	<MQL-1442 ng/g		3–9 ng/g	[57]
PCPs (musks)	Sodium acetate buffer (pH: 3.4) and n-hexane	Aluminum oxide column	GC-MS	80–105	23–20,000 ng/g	5–25 µg/L	10–50 µg/L	[96]
PCPs and steroids (14)	ACN–ethyl acetate (5:1; *v*/*v*)	SPE (silica cartridge)	LDTD-APCI-MS/MS	80–109	nd, ≤LMD-106 ng/g	2.8–16.8 ng/g (MDL)		[41]
PCPs (19 biocides)	MeOH and MeOH-0.1%/*v*/*v*) formic acid in Milli-Q water (5:5, *v*/*v*)	SPE (Oasis HLB^®^)	UHPLC-MS/MS	70–120	ND-887 ng/g	-	0.01–6.37 ng/g	[81]
PCPs (TCB and TCC)	ACN	SPE (HLB)	LC-MS/MS	33.1–117.4	~0.04–6.5 μg/g ^b^	0.0024–0.006 μg/g		[97] ^1^
PCPs and EDCs (6 retinoids 7 EDCs)	Ethyl acetate	Silica gel column/anhydrous sodium phosphate, silica gel, and glass wool)	HPLC-MS/MS	63–182	nd, 9–22,900 ng/g	0.17–4.3 ng/g	0.56–31 ng/g	[98]
PCPs and EDC (5 retinoids and 7 EDCs 7)	Ethyl acetate	Silica gel column/anhydrous sodium phosphate, silica gel, and glass wool)	HPLC-MS/MS	Zhou et al. [98]	0.34–1800 ng/g	Zhou et al. [98]	Zhou et al. [98]	[99]
PCPs (TCC and transformation products)	Phosphate buffer (pH 2), ACN	SPE (HLB)	UHPLC-MS/MS	105.18–317.64	1700–12,790 ng/g	0.09–1.44 ppb	0.25–5.22 ppb	[100]
PCPs (5 musks)	*n*-hexane–acetone (3:1, *v*/*v*)	EnviCarb cartridges 120/400	GC-MS	63.20->100	<LOD-21,294 ng/g	0.1–210 ng/g	1–526 ng/g	[101]
PCPs (TCB)	MeOH	Filtrate 0.22 μm organic phase membrane	HPLC		13.5–23.4 μg/g			[102]
PCPs (TCS and metabolites)	ACN + phosphate buffer	SPE (Oasis HLB^®^)	UPLC	1–19.3–145.8	15–4532 ng/g	0.1–0.6 ppb	0–1 ppb	[103]
PCPs (musks)	Sodium acetate buffer (pH: 3.4) and n-hexane	SPE (Strata SI—silica)	GC/MS/MS	49.7–112.2	<MLOD-8399 ng/g	MLOD: 0.4–2 ng/g	MLOD: 0.4–2 ng/g	[104]
PhACs (16)	MeOH–acetone (7:2, *v*/*v*)	SPE (Oasis HLB^®^)	HPLC	41.1–115			1.39–360	[105]
PhACs (18 estrogens)	Ethyl acetate	Silica gel cartridge and diluted in ethyl acetate/methanol (90:10, *v*/*v*)	RRLC-MS/MS	62.6–138	nd, <LOQ-372 ng/g	0.08–2.06 ng/g	0.34–6.86 ng/g	[25]
PhACs and EDCs (9)	MeOH–water (2.5:1.5, *v*/*v*)	SPE (C18)	Derivatization + GC/MS	84.6–107	<LOD-6560 ng/g	15–33 ng/g	59–108 ng/g	[35]
PhACs and EDCs (7)								
PhACs (15 antidepressants)	MeOH–0.1 acetic buffer solution pH 4.0 (1:1, *v*/*v*)	SPE (Strata-X-C)	LC-qQMS	44–101	nd, 3.3–3735 ng/g	0.04–0.5 ng/g	0.1–1.7 ng/g	[106]
PhACs (4 antibiotic and 2 estrogens)	1 M citrate buffer (pH 4.7) + MeOH–water (60:40)	SPE (SAX + HLB for antibiotics; Carboprep/NAX for estrogens)	LC–MS/MS.	17–59	<LOD, 5600–7600. ng/g	0.6–8.5 ng/g	1.1–17.1 ng/g	[107]
PhACs (22 antibiotics)	Citric acid buffer (pH 3)	SPE (Oasis HLB^®^)	LC-MS/MS	50–150	nd, 1.45–5800 ng/g	MDLs: 0.45–8.57 ng/g	MQLs: 1.50–28.6 ng/g	[108]
PhACs (13 quinolones antibiotics)	MeOH–McIlvaine (50:50; *v*/*v*) pH:3		LC–MS/MS.	96.1–103.6	12–834 ng/g	2–5 ng/g	6–18 ng/g	[36] ^a^
PhACs (NSAIDs, lipid regulators and antibiotics)	MeOH–water (1:1; *v*/*v*)		LC-MS/MS	76–131	<LOD-1125 ng/g	<LOD-1125 ng/g	-	[58]
PhACs (21 progestogens)	Ethyl acetate–MeOH (8:2, *v*/*v*)	Silica gel cartridge	UHPLC-MS/MS with ESI (under positive ionization mode)	35–129	nd, 1.2–1952 ng/g	0.01–3.68 ng/g	0.01–12.30 ng/g	[25]
PhACs (26)	MeOH–0.2 M citric acid buffer, pH: 4.4, (1:1 *v*/*v*)	SPE (Oasis HLB^®^)	UHPLC-MS-MS	54–130	<LOD-8546.21 ng/g	0.01–0.50 ng/g	0.02–1 ng/g	[109]
PhACs (13)	MeOH–water (50:50) 0.5% HCOOH		LC-MS/MS QqQ	20–117	30–7500 ng/g	-	1.2–46 ng/g	[110,111]
PhACs (8)	McIlvaine buffer (0.12 M EDTA, pH 3.5) + ACN	QuEChERS	SPE–UHPLC–MS-MS	58–118	nd, <MLOQ-4784 ng/g	1–180 ng/g	9.1–1230 ng/g	[31]
PhACs (11 acidic drugs and estrogenic hormones)	Phosphate buffer (pH 2) solution and ACN (15:10, *v*/*v*)	SPE (ENVI-18)	GC-MS	70.6–133	29.6–1796 ng/g	0.7–5.2 ng/g	2–15.6 ng/g	[80]
PhACs (5)	MeOH–acetone (7:2, *v*/*v*)	SPE (Oasis HLB^®^)	UPLC		13–76 ng/g ^b^			[112]
PhACs (5 NSAIDs)	Water–hexane–acetone	Not specified	LC-MS/MS		0.5–250 ng/g	Linked to a previous study		[23]
PhACs (4)	ACN + phosphate buffer	SPE (C18)	UPLC	81.8–98.1	252–655 ng/g	0.1–1 ng/mL	0–5.0 ng/mL	[113]
PhACs (5)	MeOH–acetone (7:2, *v*/*v*)	SPE (Oasis HLB^®^)	UPLC	76.2–86.7	33.6–2206 ng/g	MDL: 30–710 ng/L	MQL: 70–1890 ng/L	[114]
PhACs (69)	MeOH–water solution (pH: 2.5, 0.5% HCOOH and 0.1% disodium-EDTA (50/50, *v*/*v*)	Filtered by RC 0.22 μm syringe filter	LC-MS/MS	53–162	MDL, 2.19–3808 ng/g	0.3–9.1 ng/g	1–28 ng/g	[115]
PhACs and illicit drugs (148)	MeOH–Milli-Q water (pH 2.5), FA 0.5% and 0.1% EDTA, (50:50 *v*/*v*)	Filtered through a 0.2 μm RC syringe filter	HPLC-MS/MS	20–119	<LOD-267 ng/g	0.9–19.9 ng/g	20–66.3 ng/g	[79]
PhACs (7 antibiotics)	MeOH–formic acid (0.5% *v*/*v*=	SPE (C18)	LC-MS/MS	9–94	27–191 ng/g	MDL: 0.002–12.5 ng/g	MQL: 0.003–25 ng/g	[9]
Plasticizers/PCPs (3 alkylphenols)	Hexane–DCM (1:1, *v*/*v*) + DCM–acetone (1:1, *v*/*v*)	Florisil^®^ cartridges	GC-MS	-	Study of stability	Lower warming limit: 0–50 mg/kg	Upper warming limit: 16–110 mg/kg	[86]
Plasticizers (2 butyltin compounds)	Acetic acid		Derivatization with NaBEt4+ GC-ICP-MS		534–1569 ng SN/g			[26] ^a^
Plasticizers (7)	MeOH–acetone (50:50, *v*/*v*)	SPE (HLB and MAX cartridge)	LC-MS/MS	57.1–101.9	Method does not apply in SS samples	0.03–0.86 ng/g	0.09–0.03 ng/g	[56]
Plasticizers/PCPs (4 nonylphenols ethoxylates)	ACN		GC/MS	93.5–137.8	5.5–19.5 ng/g	0.03–12 ng/g		[116]
Plasticizers (9)	MeOH–water pH 12 (5:3, *v*/*v*)	THPE-DMIP column	HPLC–MS/MS	43.6–96.7	nd, 0.26–63.6 ng/g	-	MLOQ: 0.0004–8.28 ng/L	[117]
PPCPs (17 azoles)	MeOH	SPE (C18)	LC-MS/MS	71.9–115.8	n.d., <LOQ-4448.9 ng/g	0.5–5 ng/g	2.0–16.5 ng/g	[118]
PPCPs and industrial products (10)	ACN–water (5:3, *v*/*v*)	SPE (Oasis HLB^®^)	UHPLC-MS/MS	65.3–125.3	1.7–5088.2 ng/g		0.1–3 ng/g (MQLs)	[20]
PPCPs (5 azoles)	MeOH–formic acid (100:0.1 *v*/*v*)	SPE (Oasis HLB^®^)	1200 HPLC system coupled to an Agilent 6410 triple-quadrupole mass spectrometer with electrospray ionization used in positive mode	71.2–94.9		4.9–616 ng/g	MQL: 3–29 ng/g	[82]
PPCPs (7 antibiotics and antibacterial agents)	50 % ACN in 1mM EDTA solution (pH 2.0 with HCl)	SPE (Oasis HLB^®^)	HPLC-MS/MS	41–123	nd, <LOQ, 4–17,740 ng/g	-	10–500 ng/g	[119]
PPCPs (14)	MeOH–formic acid (100:1, *v*/*v*)	EnviCarb cartridges	Derivatization + GC/MS	57.9–103.1	<LOD-1965 ng/g	1.6–11 ng/g	4.7–39 ng/g	[37]
PPCPs (10)	MeOH–water (9: 1, *v*/*v*, pH 11) + acetone + water with 0.1% formic acid (pH: 2.65)	SPE (Oasis HLB^®^)	UHPLC-APCI-SRM/MS	81.1–156	nd, <LOD-297.04 ng/g	0.01–14.79 ng/g		[120]
PPCPs (22)	Milli-Q water (pH 9)	Online DI-SPME-	On-fiber derivatization. GC-MS	53.98–105.15	It was not applied in solid samples	0.64–253.30 ng/g	7.03–844.33 ng/g	[61]
PPCPs and metabolites (19)	MeOH (0.5% *v*/*v*, formic acid)	dSPE (PSA + C18)	LC-MS/MS	22–99	Validation method	MDL: 0.1–5.3 ng/g	MQL: 0.4–18 ng/g	[121]

R: recovery; LOD: limit of detection; LOQ: limit of quantification; Ref: reference; HBCD: hexabromocyclododecane; TBBPA: tetrabromobisphenol A; PCPs: personal care products; PhACs: pharmaceutical compounds; RC: regenerated cellulose; NSAIDs: nonsteroidal ant-inflammatory drugs; APEs: alkylphenol ethoxylates; BDEs: brominated diphenyl ethers; OPFRs: organophosphate flame retardants; PBDEs: polybrominated diphenyl ethers; NBFRs: novel brominated flame retardants; PFAS: per- and polyfluoroalkyl substances; EDCs: endocrine-disrupting compounds; PPCPs: pharmaceutical and personal care products; TCS: triclosan; TCC: triclocarban; UAE: ultrasound-assisted extraction; SPE: solid-phase extraction; HLB: hydrophilic–lipophilic; DI-SPME: direct-immersion solid-phase microextraction; DI-SPME: direct-immersion solid-phase microextraction; DCM: dichloromethane; MeOH: methanol; ACN: acetonitrile; THF: tetrahydrofuran; EDTA: ethylenediaminetetraacetic; THPE: 1;1;1-Tris(4-hydroxyphenyl) ethane; DMIP: dummy molecularly imprinted polymer; LC-QqLIT-MS: liquid chromatography–quadrupole linear ion trap mass spectrometry; GC-MS-: gas chromatography–mass spectrometry; GC/MS-NCI: GC-MS–negative chemical ionization; LC-MS/MS: liquid chromatography–tandem mass spectrometry; GC-ICP-MS: gas chromatography–inductively coupled plasma–mass spectrometry; LC-qQMS: triple-quadrupole mass spectrometer; RRLC-MS/MS: rapid-resolution liquid chromatography–tandem mass spectrometry; LC-MS/MS QqQ: liquid chromatography–tandem mass spectrometry infused in the triple-quadrupole. RL: reporting levels; UHPLC-MS/MS: ultra-high-performance liquid chromatography–tandem mass spectrometry; HFBA: heptafluorobutyric anhydride; QuEChERS: quick; easy; cheap; effective; rugged; and safe; nd: non-detected; MQL: method quantification limit. ^1^ According to method described by EPA [122]. ^2^ According to method described by EPA [123]. ^a^ Different extraction methods were compared, and there were no significant differences between them. ^b^ Data obtained from a plot.

**Table 5:** Determination of EPs in sewage sludge, based on microwave-assisted extraction (MAE).

Analytes	Extraction Solvent	Clean-Up	Detection Technique	R (%)	Range of Concentration	LOD	LOQ	Ref.
EDCs (12)	MeOH	SPE (C18)	LC-MS/MS	71.7–103.1	<LOD-710.2 ng/g	0.6–3.5 ng/g	2.0–11.6 ng/g	[127]
EDCs (2 butynyl compounds)	Acetic acid		Derivatization with NaBEt4+ GC-MS; GC-ICP-MS		534–1569 ng SN/g			[26] ^a^
EDCs (13)	MeOH	SPE	Derivatization GC/MS	92–102	nd, 36–164 ng/kg	0.5–4.5 ng/kg	2–15 ng/kg	[124]
EDCs (hormones and corticoids)	MeOH	None required	UHLPC-MS/MS	60–130	nd, <LOQ-1440 ng/g	2.1–192.8 ng/g		[125]
EDCs (14 phenols)	30 mL of 1:1 (*v*/*v*) acetone–hexane, and 500 μL of glacial acetic acid	Silica column + washed with DMC/hexane	GC-MS	71–105	<4–337,200 ng/g	MDL: 2.7–204 ng/g		[18]
EDCs (BPA)	Acetone–hexane (1:1, *v*/*v*)	SPE (LC-18)	HPLC–UV	53–90	125–180 ng/g	100 ng/g	330 ng/g	[24] ^1^
EPs (61)	For illicit drugs: MeOH-DCM (50:50, *v*/*v*)// For PhACs: ACN–water (70:30, *v*/*v*, pH: 2)	SPE (Oasis MCX^®^)	LC/MS	90–104	< LOD-2426.9 ng/g	0–27 ng/g		[131]
PCPs (4 LAS)	MeOH		LC-FLD	35–98	0.70–13.39 mg/kg	3.3–5.4 ng/g	11.0–18.0 ng/g	[128]
PhACs (4 NSAIDs)	Water	DME + SPE (Oasis HLB^®^)	GC-MS(SIS)	80–105	10–140 ng/g		15–29 ng/g	[129]
PhACs (5 fluoroquinolone antibiotics)	HTAB (non-ionic surfactant used as an extractant) and 5% (*v*/*v*) surfactant concentration in Milli-Q water		LC-MS/MS	73.2–95.6	nd, 9.57–206.1 ng/g	0.15–0.55 ng/g	0.49–1.85 ng/g	[130]
PhACs (13 quinolones antibiotics)	MeOH–McIlvaine (50:50, *v*/*v*, pH:3)		LC-MS/MS	96.1–103.6	12–834 ng/g	2–5 ng/g	6–18 ng/g	[36] ^a^
PhACs and Illicit drugs (18)	MeOH–water (1:1, *v*/*v*)	SPE (Oasis HLB^®^)	LC-MS/MS	46.9–187.3	0.4–275.2 ng/g	MDL:0.02–32.73 ng/L	MQL: 0.07–109.08 ng/L	[132]
PhACs and stimulants (5 antidepressants and caffeine)	MeOH-ACN (43:57) pH 3		HPLC-PDA	60–99	24–1980 ng/g	15–50 ng/g	100–200 ng/g	[133,134]
Plasticizers (phthalates)	Hexane–acetone (1:1, *v*/*v*)	Silicone–alumina column packed	GC-MS	nd	nd	nd	nd	[135]
PPCPs (22)	MeOH–water (3:2, *v*/*v*)	SPE (Oasis HLB^®^)	GC-MS	91–100	nd-3100 ng/kg	0.8–5.1 ng/kg		[136]
PPCPs and illicit drugs (90)	MeOH–water (50:50, *v*/*v*, pH:2)	SPE (Oasis MCX^®^)	UPLC-MS/MS	40–152	<MQL-5800 ng/g	0.03–4.81 ng/g	0.14–24.05 ng/g	[126]

R: recovery; LOD: limit of detection; LOQ: limit of quantification; Ref: reference; PhACs: pharmaceutical compounds; NSAIDs: nonsteroidal anti-inflammatory drugs; PCPs: personal care products; EDCs: endocrine-disrupting compounds; PPCPs: pharmaceutical and personal care products; BPA: bisphenol A; MAE: microwave extraction; MAME: microwave-assisted micellar extraction; DME: dispersive matrix extraction; SPE: solid-phase extraction; HLB: hydrophilic–lipophilic balance; MXC: mixed-mode cation exchange; MeOH: methanol; ACN: acetonitrile; HTAB: hexadecyltrimethylammonium bromide; PET: polyester; GC-MS(SIS): gas chromatography–mass spectrometry in selected ion storage mode; LC-MS/MS: liquid chromatography–tandem mass spectrometry; LC-FLD: liquid chromatography with fluorescence detection; GC-MS: gas chromatography—mass spectrometry; GC-ICP-MS: gas chromatography—inductively coupled plasma—mass spectrometry; UHPLC-MS/MS: ultra-high-performance liquid chromatography–tandem mass spectrometry; UPLC-MS/MS: ultra-high-performance liquid chromatography–tandem mass spectrometry; HPLC-UV: high-performance liquid chromatography–ultraviolet detection; HPLC-PDA: HPLC coupled to a photodiode array ultraviolet detector; nd: non-detected. ^1^ According to method described by EPA [137]. ^a^ Different extraction methods were compared, and there were no significant differences between them.

**Table 6:** Determination of EPs in sewage sludge, based on pressurized liquid extraction (PLE).

Analytes	Extraction Solvent	Clean-Up	Detection Technique	R (%)	Range of Concentration	LOD	LOQ	Ref.
EDCs (estrogens and BPA)	Acetone–MeOH (1:1, *v*/*v*)	SPE (Oasis HLB^®^)	LC-MS/MS	88–97 (absolute)	0.7–92.9 ng/g	0.05–0.20 ng/g	0.1–0.5 ng/g	[81]
EDCs and caffeine (22)	Water–MeOH-acetone (1:2:1, *v*/*v*)	Column clean-up	TFC-LC-MS/MS	53–115	nd, 2.6–29,416 ng/g	0.031–321 ng/g	0.10–1071 ng/g	[148]
EPs (pharmaceuticals, PFOA, PFOS, 44)	Milli-Q water–MeOH (1:1, *v*/*v*)	2 SPE (OASIS MCX and OASIS HLB^®^)	HPLC-MS/MS	>70	nd-5 µg/g	0.06–14.38 ng/g	0.12–47.92 ng/g	[152]
Domestic products (8 sweeteners)	MeOH	SPE (Oasis HLB^®^)	LC-ESI-MS/MS	29–87	nd, <LOQ-481 ng/g	-	5–10 ng/g	[139]
Flame retardants (15)	Toluene	Diatomaceous earth	UPC-MS/MS	65–112	<0.005–1208 ng/g	0.020–6 pg/g	0.005–1.3 ng/g	[149]
Flame retardants (DBDPE)	Hexane–DCM (1:1, *v*/*v*)	Purification Power Prep™ (acidic silica gel, basic alumina, and carbon columns)	HRGC-TQMS/MS	63	3.25–125 ng/g	0.3 pg/g		[153]
Flame retardants (7 BDEs)	Hexane–DCM (50:50, *v*/*v*)	C18	GC-MS/MS	92–102	nd, 0.21–19.6 ng/g	0.01–0.04 ng/g	-	[40]
Flame retardants and PCPs (99 PCBs, musk, etc.)	20% DCM *n*-hexane	GPC (for PLE), Silica (for SPLE)	GC-MS	28–219/4–287	Not quantified	0.02–129.43 ng/g	0.12–392.2 ng/g	[50]
Flame retardants (9)	Acetone–DCM (1:1, *v*/*v*)	Bond Elut-NH2 SPE	HPLC-MS/MS	-	0.02–349.20 g/day	-	-	[47]
Industrial/domestic products (PFPAs/PFOS)	Tetrahydrofuran–water (25:75, *v*/*v*)	SPE (Oasis WAX)	LC-MS/MS	75–85	0.07–48 ng/g	0.01–0.25 ng/g	-	[147]
Industrial/domestic products (18 PFCs)	MeOH	SPE (Oasis WAX 3cc)	LC–QLiT-MS/MS.	76–111	<MLOD-121.1 ng/g	15–837 ng/kg	50–2772 ng/kg	[154]
Illicit drugs (20)	MeOH–water (9:1, *v*/*v*)		LC-ESI-(QqLIT)MS/MS)	55–129	nd, 0.7–579.0 ng/g	0.1–6.4 ng/g	0.3–22.5 ng/g	[150]
PCPs (10 musks)	MeOH–water (1:1, *v*/*v*)	Florisil^®^ cartridges	GC–MS/MS	63–100	nd, <LOQ-530.5 ng/g	0.5–1.5 ng/	2.5–5 ng/g	[28]
PCPs (TCS and transformation products)	DCM	GPC (gel permeation chromatography) + multilayer silica column	GC/MS	88–99	nd, 51–2505.9 ng/g	1–10 ng/g	3–30 ng/g	[151]
PhACs (10 β-blockers)	MeOH–water-acetic acid (49/19/2, *v*/*v*)	SPE (Oasis MCX^®^)	LC-MS/MS	76–149	2–95 ng/g	-	0.5–15 ng/L	[145]
PhACs (5 estrogens)	Water–MeOH (80:20, *v*/*v*)	SPE (Oasis HLB^®^)	LC-MS/MS	86–126	4.2–63 ng/g	-	1–5 ng/g	[19]
PhACs (carbamazepine)	MeOH	SPE (C18)	LDTD-APCI-MS/MS	96.9–107	13–94 ng/g	3.4 ng/g	-	[141] ^a^
PhACs (14)	MeOH–McIlvaine buffer (1:1, *v*/*v*)	SPE (Oasis HLB^®^)	HPLC-MS/MS	66.6–118.5	97.6–268.0 ng/g	0.2–5.8 ng/g	0.6–19.4 ng/g	[52]
PhACs (22 sulfonamide antibiotics)	ACN–water (25:75, *v*/*v*)	SPE (Oasis HLB^®^)	LC-MS/MS	19–130	0.22–175 ng/g	0.03–17.40 ng/g	0.10–58.00 ng/g	[140]
PhACs (13 quinolones antibiotics)	MeOH–McIlvaine (50:50; *v*/*v*) pH:3		LC–MS/MS.	96.1–103.6	12–834 ng/g	2–5 ng/g	6–18 ng/g	[36] ^b^
PhACs (9 glucocorticoids)	MeOH–acetone (80:20, *v*/*v*)	SPE (Bond Elut Plexa cartridges)	UHPLC-MS-MS	8–73	nd, <LOQ-6.1 μg/kg	0.5–1 ng/g	1–5 ng/g	[155]
PhACs (2 anticancer drugs)	MeOH–ultrapure water (65:35, *v*/*v*)	SPE (MAX/MCX cartridges)	UHPLC-MS/MS	92–106	<MQL-42.5 μg/kg	2.5–74 ng/g	6.1–186 ng/g	[146]
PhACs (8 sedative hypnotics)	MeOH–water (1:1, *v*/*v*)		LC-MS/MS	88–112	nd, <LOQ-18.9 μg/kg	0.2–12 ng/g		[156]
PhACs (5 antibiotics)	MeOH-ACN-0.2 M citric acid (pH: 4.5) (40:40:20)	SPE (StrataX cartridges)	LC-MS	33.5–91.4	nd, 55–8492 ng/g	1.5–3.8 ng/g	5–20 ng/g	[144]
PhAcS (12)	MeOH	SPE	HPLC-MS/MS	33–125	<LOQ430 ng/g	0.4–20 ng/g	1.2–68 ng/g	[157]
PhACs (diclofenac)	MeOH	SPE (C18)	LDTD-APCI-MS/MS	95.6	530–650 ng/g	270 ng/L	1000 ng/L	[158]
PPCPs and illicit drugs (14)	MeOH or MeOH–formic acid (100:0.1, *v*/*v*)		UHPLC	56–128	<LOD-5940 ng/g	1–50 ng/g	-	[159]
PPCPs and flame retardants (71)	Acetone, MeOH, heptane, acetate buffer, citric acid, etc., depend on the compound	SPE (StrataX, Oasis HLB, multilayer silica) or without clean-up	HPLC-MS or GC-MS	20–103	<LQ, 2.7–105,536 ng/g		1–12,710 ng/g	[160] ^c^
PPCPs and metabolites (19)	MeOH (0.5% *v*/*v*, formic acid)	C18+PSA	LC-MS/MS	42–111	Validation method	MDL: 0.1–3.5 ng/g	MQL: 4.9–12 ng/g	[121]
PPCPs (17)	MeOH–water (1:1, *v*/*v*)	-	LC-QqQ-MS	-	nd-5176.6 ng/L	-	-	[161]

R: recovery; LOD: limit of detection; LOQ: limit of quantification; Ref: reference; PhACs: pharmaceutical compounds; PFAS: per- and polyfluoroalkyl substances; PFOs: perfluorooctanesulfonic acids; PFCs: perfluorinated compounds; BDEs: brominated diphenyl ethers PPCPs: pharmaceutical and personal care products; EDCs: endocrine-disrupting compounds; BPA: bisphenol-A; PCBs: polychlorinated biphenyls; PLE: pressure liquid extraction; MeOH: methanol; ACN: acetonitrile; SPE: solid-phase extraction; MXC: mixed-mode cation exchange; HLB: hydrophilic–lipophilic balance; WAX: weak anon exchange; LLE: liquid–liquid extraction; AAE: aqueous alkali extraction; GPC: gel permeation chromatography; LC-MS/MS: liquid chromatography–tandem mass spectrometry; UPLC-MS/MS: ultra-high pressure liquid chromatography–tandem mass spectrometry; UHPLC-MS: ultra-high pressure liquid chromatography–mass spectrometry; LC-QLiT-MS/MS: liquid chromatography and analysis in a hybrid-quadrupole–linear ion trap mass spectrometer; UHPCL-MS/MS: ultra-high pressure liquid chromatography–tandem mass spectrometry; LDTD-APCIT-MS/MS: laser diode thermal desorption–atmospheric pressure chemical ionization coupled with tandem mass spectrometry; HRGC-TQMS/MS: high-resolution gas chromatography–triple-quadrupole mass spectrometer; GC-MS/MS: gas chromatography–mass spectrometry; HPLC-MS/MS: high-performance liquid chromatography and tandem spectrometry; LC-ESI-(QqLIT)MS/MS: liquid chromatography–(electrospray) operating in positive ionization mode connected in series with hybrid-triple-quadrupole–linear ion trap tandem mass spectrometry; TFC-LC-MS/MS: turbulent flow chromatography followed by liquid chromatography coupled with tandem mass spectrometry; LC-ESI-MS/MS: liquid chromatography–(electrospray) tandem mass spectrometry; LDTD-APCI-MS/MS: diode thermal desorption/atmospheric pressure chemical ionization coupled with tandem mass spectrometry; nd: non-detected; LOD: limit of detection (<LOD: below LOD); LOQ: limit of quantification (<LOQ: below LOQ); <MLOD: below method’s limit of quantification; <LOQ: below method’s quantification limits. ^a^ More extraction techniques were used, and this showed the best efficiency. ^b^ Different extraction methods were compared, and there were no significant differences between them. ^c^ In this study, many different compounds were determined and, according to the compounds’ different clean-up steps, were applied.

**Table 7:** Determination of EPs in sewage sludge, based on pressurized hot water extraction (PHWE).

Analytes	Extraction Solvent	Clean-Up	Detection Technique	R (%)	Range of Concentration	LOD	LOD	Ref.
Industrial application (aliphatic primary amines)	Water (pH 4	Diatomaceous earth	GC–MS		0.17–543 m/kg	nd–543 ng/kg	nd–543 ng/kg	[27]
Industrial application (nitrosamines)	Milli-Q water pH 7.5	HS-SPME	GC-CI-MS-MS		nd, <LOD-371 ng/g	0.03–0.14 ng/g	0.03–0.14 ng/g	[62]
PhACs (NSAIDs)	NaOH (0.01 M)	HF-LPME	LC-ESI-MS	38.9–90.3	7.7–588 ng/g	0.4–3.7 ng/g	0.4–3.7 ng/g	[34]
PhACs (10 azoles)	Ultrapure water	SPE (Oasis HLB^®^)	LC-Orbitrap-HRMS	25–107	<LOQ-255.4 ng/g	0.25–25 ng/g	0.25–25 ng/g	[29]
PhACs (23)	Water pH 7	SPE (Oasis HLB^®^)	UPLC-MS/MS	16–37 (absolute)		nd, 0.4–3009 ng/g	nd, 0.4–3009 ng/g	[163]

R: recovery; LOD: limit of detection; LOQ: limit of quantification; Ref: reference; PhACs: pharmaceutical compounds; HF-LPME: hollow-fiber liquid-phase microextraction; HS-SPME: headspace solid-phase microextraction; HLB: hydrophilic–lipophilic balance; GC-MS: gas chromatography–mass spectrometry; LC-ESI-MS: liquid chromatography–(electrospray) mass spectrometry; GC-CI-MS/MS: gas chromatography–ion trap tandem mass spectrometry; LC-Orbitrap-HRMS: liquid chromatography–high-resolution mass spectrometry; UPLC-MS/MS: ultra-performance liquid chromatography–tandem mass spectrometry; nd: non-detected.

**Table 8:** Determination of EPs in sewage sludge, based on QuEChERS.

Analytes	Extraction Solvent	Clean-Up	Detection Technique	R (%)	Range of Concentration	LOD	LOQ	Ref.
EPs (42)	ACN	d-SPE	LC-PDA-FLD	50–126	<LOQ-2.05 µg/g	0.007–0.271 µg/g	0.0240–0.821 µg/g	[172]
PCPs (13 azoles and benzenesulfonamide derivates)	Cool water + ACN	d-SPE (Z-sep+)	LC-(Orbitrap)HRMS	80–90	nd, <LOQ-181.2 ng/g	0.5–10 ng/g	1–25 ng/g	[29]
PCPs (19 musks and UV filters)	ACN	dSPE (MgSO_4_ + C18 + PSA)	GC-MS/MS	74–122	nd, <MQL-115,486 ng/g	MDL:0.5–1394 ng/g	MDL: 2–4648 ng/g	[171]
PhACs (136)	S1: 0.1 M EDTA + ACN + acetic acid 1% (*v*/*v*), S2: heptane, S3: acetate buffer		LC-TOF-MS	21–135	nd, <MLQ-5957 ng/g	1–2500 ng/g for the majority of the compounds	15–6250 ng/g for the majority of the compounds	[165]
PhACs (13 NSAIDs)	Water–ACN (1:2, *v*/*v*)	Online-SPE	LC-MS/MS	36–101	<0.39–57.1 ng/g	MDL: 0.065–6.7 ng/g	-	[170]
PhACs (12)	ACN-H_2_0 (50:50) + 0.5% formic acid + 0.2% Na_2_EDTA	SPE (PSA) not used to ERY and CIPRO	HPLC-MS/MS	68–104		0.3–21.4 ng/g	1.7–71.4 ng/g	[173]
PhACs (33)	EDTA 0.1 M	d-SPE	UHPLC-Orbitrap MS	60–98	<LOQ-506.5 ng/f	0.3–8.1 ng/g	1.1–25 ng/g	[46]
PhACs (72)	McIlvaine–EDTA	Oasis HLB PRiME	LC-HRMS	>80% ^a^				[43]
PhACs (32)	McIlvaine–EDTA	Oasis HLB PRiME	LC-HRMS	>80% ^a^				[174]
PhACs (17 antibiotics)	0.2M Na_2_EDTA (in water), CAN, and MeOH	d-sSPE (MgSO_4_ + PSA)	LC-MS/MS	20–147	<MLOQ-2894 ng/g	0.003–120.39 ng/g	0.05–364.81 ng/g	[175]
PPCPs (12)	ACN acidified with acetic acid	d-SPE (chitin)	LC-ESI-MS/MS	50–120	nr	0.8–15 ng/g	5–50 ng/g	[168]
PPCPs (21)	ACN acidified with acetic acid	dSPE (PSA)	UPLC-MS/MS	50–96	nd above LOQ	0.15–1.5 ng/g	0.5–10 ng/g	[169]
PPCPs (more than 100)	ACN	dSPE (PSA)	LC-HRMS	-	Method validation			[30]
PPCPs and flame retardants (71)	Different solvents (acetone, MeOH, heptane, acetate buffer, citric acid, etc.)	dSPE	HPLC	20–103	<LQ, 2.7–105,536 ng/g		1–12,710 ng/g	[160]
PPCPs and metabolites (19)	MeOH (0.5% *v*/*v*, formic acid)	dSPE	LC-MS/MS	27–86	Validation method	MDL:0.2–16 ng/g	MQL: 0.6–54 ng/g	[121]

R: recovery; LOD: limit of detection; LOQ: limit of quantification; Ref: reference; PhACs: pharmaceutical compounds; PCPs: personal care products; PPCPs: pharmaceutical and personal care products; NSAIDs: nonsteroidal anti-inflammatory drugs; ACN: acetonitrile; d-SPE: dispersive solid-phase extraction; PSA: primary secondary amine; Z-sep+: zirconium-based sorbent; SPE: solid phase extraction; LC-TOF-MS: liquid chromatography–time-of-flight–mass spectrometry; LC-ESI-MS/MS: liquid chromatography–electrospray ionization–tandem mass spectrometry; UPLC-MS/MS: ultra-high liquid chromatography–tandem mass spectrometry; LC-(Orbitrap)-HRMS: liquid chromatography–Orbitrap–high-resolution mass spectrometry; LC-MS/MS: liquid chromatography–tandem mass spectrometry; LC-HRMS: liquid chromatography–high-resolution mass spectrometry; GC-MS/MS: gas chromatography–tandem mass spectrometry; HPLC–MS/MS: high-performance liquid chromatography–tandem mass spectrometry; nd: non-detected; MLQ: method limit quantification. ^a^ Based on a previous validation method focused on earthworms extraction [176].

**Table 9:** Determination of EPs in sewage sludge, based on matrix solid-phase dispersion (MSPD).

Analytes	Extraction Solvent	Clean-Up	Detection Technique	R (%)	Range of Concentration	LOD	LOQ	Ref.
Flame retardants (8 organophosphates compounds)	ACN	PSA sorbent	LC-QTOF-MS	69–123	nd, 2.2–1786 ng/g		2–50 ng/g	[187]
PCPs (TCS and MTCS)	DCM	Diatomaceous earth and silica with H_2_SO_4_	GC-MS	86–113	15–2640 ng/g	-	6–7 ng/g	[181]
PCPs (TCS and MTCS)	ACN		GC-MS	98.4–101.0	4–2987 ng/g	0.10–0.12 ng/g	0.3–0.4 ng/g	[180]
PCPs (9 parabens)	Ethyl acetate-MeOH (90:10, *v*/*v*).		GC–MS/MS	80.4–124.9	<LOQ-44.1 ng/g	MDLs: 0.1–1.7 ng/g	0.3–5.1 ng/g	[188]
PCPs (10 UV stabilizers)	Ethyl acetate	SPE (PSA sorbent)	GC-QTOF-MS	70–93	nd, 6.4–292 ng/g		2–10 ng/g	[182]
PhACs (8 azoles)	MeOH to recover	SPE (SCX)	LC-ESI-QTOF-MS	70–118	nd, 8–800 ng/g	-	5–8 ng/g	[183]
PhACs (2 cardiac drug)	MeOH to recover	SPE (SCX)	LC-ESI-QTOF-MS	95–11	22–362 ng/g	-	5–8 ng/g	[186]
PhACs (5 NSAIDs)	Hexane-acetone (1:2, *v*/*v*) to recover	Florisil^®^ cartridges and Silica	LC–ESI-QTOF-MS	84.0–104.5	nd, 1.8–14.7 ng/g	-	0.005–0.05 ng/g	[179]
PhACs (5 azoles)	MeOH-ACN-Formic acid (30:69:1)	SPE cartridges online	UPLC-MS/MS	82–124	nd, <LOQ-1219 ng/g	-	2–10 ng/g	[53]
PPCPs (45)	MeOH and acetonitrile/5 % oxalic acid (8/2, *v*/*v*) to do the elution	SPE (C18)	LC-QqQ-MS	50.3–107	nd, >100–2770 μg/kg		MQL: 0.117–5.55 μg/kg	[185]
PPCPs (27)	MeOH	Not specified	HILIC-MS/MS	50–120	nd, <1.25–5466 ng/g		MQL: 1.25–1250 μg/kg	[184]
PPCPs (68)		PSA sorbent	UPLC-QTOF-MS	49.7–112.2	200–8000 ng/g ^b^	MLOD: 0.4–2 ng/g		[189]
ECs (60)	MeOH	SPE(C18)	UPLC-QTOF-MS	79–113	Máx. 100 ng/g		LOQs: 0.3 ng/g–45 ng/g	[189]

R: recovery; LOD: limit of detection; LOQ: limit of quantification; Ref: reference; PCPs: personal care products; TCS: triclosan; MTCS: methyl triclosan; PhACs: pharmaceutical compounds; NSAIDs: nonsteroidal anti-inflammatory drugs; DCM: dichloromethane; ACN: acetonitrile; MeOH: methanol; SPE: solid-phase extraction; PSA: primary secondary amine; SCX: strong cation exchange; GC-MS: gas chromatography–mass spectrometry; GC-QTOF-MS: gas chromatography combined with hybrid-quadrupole time-of-flight mass analyzer. LC-QTOF-MS: liquid chromatography–quadrupole time-of-flight–mass spectrometry; LC-ESI-QTOF-MS: liquid chromatography using a hybrid-quadrupole time-of-flight (QTOF)–mass spectrometer furnished with an electrospray ionization (ESI) source; LC-ESI-QqQ-MS: liquid chromatography–triple-quadrupole mass spectrometry; UPLC-MS/MS: ultra-high-performance liquid chromatography–tandem mass spectrometry; HILIC-MS/MS: hydrophilic-interaction liquid chromatography–mass spectrometry; GC-MS/MS: gas chromatography–tandem mass spectrometry; nd: non-detected; MQL: method quantification limit. ^b^ Data obtained from a plot.

**Table 10:** Determination of EPs in sewage sludge, based on others different novel extraction techniques.

Analytes	Extraction Solvent	Clean-Up	Detection Technique	R (%)	Range of Concentration	LOD	LOQ	Ref.
Flame retardants (2 PCBs) by SLE-LTP	Water-extraction mixture (composed by ACN, ethyl acetate tetrahydrofuran, isopropanol)		GC-MS-SIM	78–109	Not quantified	16–32 ng/kg	-	[206]
Flame retardants (6 PCBs 6) by SLE-LTP	Isopropanol–ethyl acetate (13:3, *v*/*v*)	Silica–sodium sulphate cartridge	GC-MS	66–94	50–70 ng/g	3.3 ng/g	10.0 ng/g	[204]
PCPs (6 musks) by MA-HS-SPME	Deionized water + 3 g NaCl + pH 1 (with HCl)		GC-MS	68–87	nd-2.8 ng/g (fresh weight)	0.04–0.1 ng/g	0.1–0.3 ng/g	[194]
PCPs (8 macrocyclic musks) by HS-SPME	Ultrapure water		GC-MS	-	nd, <MQL–1.45 ng/g	10–25 pg/g	25–50 pg/g	[195]
Plasticizers (5 phthalates) by SLE-LTP	Acetonitrile/ethyl acetate	-	GC-MS	76–119	nd, 0.06–10.24 mg/kg	-	40–80 μg/L	[205]
PhACs (4 NSAIDs) by HF-LPME	0.1M (NH_4_)_2_CO_3_ pH:9		LC-ESI-MS	not reported	29–138 ng/g	-	-	[39]
PhACs (4 NSAIDs) by HF-LPME	Acceptor buffer solution (0.1 M (NH_4_)_2_CO_3_) pH:9	C-18 column	LC-MS/MS	-	nd, 30–1480 ng/L	MDL: 0.8–14.3 ng/L		[208]
PhACs (3 metabolites from NSAIDs) by HF-LPME	Reagent water (Milli-Q water)		GC-MS	-	nr-183 ng/g	1.6–5.6 ng/g	5.3–18.6 ng/g	[38]
PPCPs (6) by SBSE	NaHCO_3_ and acetic acid anhydride		TD-GC-MS	91–110	30–280 ng/g	0.08–1.06 ng/g	0.24–3.22 ng/g	[201]

LOD: limit of detection; LOQ: limit of quantification. Ref: reference; PCPs: personal care products; PCBs: polychlorinated biphenyls; PhACs: pharmaceutical compounds; NSAIDs: nonsteroidal anti-inflammatory drugs; MA-HS-SPME: microwave-assisted headspace solid-phase microextraction; HS-SPME: headspace solid-phase microextraction; SLE-LTP: solid–liquid extraction with low-temperature purification; HF-LPME: hollow-fiber-based liquid-phase microextraction; SBSE: stir bar sorptive extraction; GC-MS: gas chromatography–mass spectrometry; GC-MS-SIM: GS-MS–selective ion mode; LC-ESI-MS: liquid chromatography–electrospray ionization–mass spectrometry; LC-MS/MS: liquid chromatography–tandem mass spectrometry; TD-GC-MS: thermal desorption–gas chromatography–mass spectrometry; nd: non-detected; <MQL: below method quantification limit.

**Table 11:** Summary table of the main parameters for validation.

Validation Parameter	Objective	Measures
Selectivity	Asses the method’s ability to accurately identify and quantify the target analyte in heterogeneous samples.	Matrix effect (ME) or interference studies
Accuracy	Evaluate the closeness of the measured value to the true value.	Recovery (acceptable range: 70–120%)
Linearity	Test the method’s ability to obtain results proportional to the analyte concentration over a specified range.	Linearity (R^2^)
Precision	Assess the repeatability (intra-day) and intermediate precision (inter-day) of the method.	%RSD (generally < 10%)
Sensitivity	Estimate the method’s ability to detect and quantify low concentrations of the analytes.	LODs and LOQs

## Data Availability

The data revised in this study are included in the references list; further inquiries can be directed to the corresponding authors.

## Supplementary Materials

The following supporting information can be downloaded at https://www.mdpi.com/article/10.3390/toxics13080661/s1. Table S1. Classification of emerging pollutants. Table S2. Details of the procedure of mechanical shaking extraction of EPs in sewage sludge. Table S3. Details of the procedure of Soxhlet extraction of EPs in sewage sludge. Table S4. Details of the procedure of UAE of EPs in sewage sludge. Table S5. Details of the procedure of MAE of EPs in sewage sludge. Table S6. Details of the procedure of PLE of EPs in sewage sludge. Table S7. Details of the procedure of PHWE of EPs in sewage sludge. Table S8. Details of the procedure of QuEChERS of EPs in sewage sludge. Table S9. Details of the procedure of MSPD of EPs in sewage sludge. Table S10. Details of the procedure of different extraction techniques in sewage sludge.

## Abbreviations

2D-GC-HRMS, two-dimensional gas chromatography with high-resolution mass spectrometry; APCI, atmospheric pressure chemical ionization; APPI, atmospheric pressure photo ionization; APs, alkylphenols; ASE, accelerated solvent extraction; BDEs, brominated diphenyl ethers; BFRs, brominated flame retardants; BIN, barrel insert and needle; BPA, bisphenol-A; BSTFA, bis(trimethylsilyl)trifluoroacetamide; CV, coefficient of variation; DAD, diode array detector; DEHP, bis(2-ethylhexyl) phthalate; dSPE, dispersive solid-phase extraction; EDCs, endocrine-disrupting compounds; EDTA, ethylenediaminetetraacetic acid; EI, electron ionization; EPs, emerging pollutants, ESE, enhanced solvent extraction; ESI, electrospray ionization; FD, fluorescence detector; FUSLE, focused ultrasound-assisted liquid extraction; GC-ICP, gas chromatography–inductively coupled plasma; GC-MS, gas chromatography–mass spectrometry; HBCDs, hexabromocyclododecanes; HDPE, high-density polyethylene; HESI, heated electrospray ionization; HF-LPME, hollow-fiber solid-phase microextraction; HFBA, heptafluorobutyric anhydride; HILIC, hydrophilic-interaction liquid chromatography; HPLC, high-performance liquid chromatography; HS-SDME, headspace single-drop microextraction; HS-SPME, headspace solid-phase microextraction; HSPE, high-pressure solvent extraction; HTAB, cationic surfactants; IQL, instrumental quantification limit; ISO, Organization for Standardization; IUPAC, International Union of Pure and Applied Chemistry; Kow, octanol–water partition coefficient; LAS, linear alkylbenzene; LC, liquid chromatography; LC-MS, liquid chromatography–mass spectrometry; LC-QqLIT-MS, liquid chromatography–quadrupole linear ion trap mass spectrometry; LIT, linear ion trap; LODs, limits of detection; LOQ, limit of quantification; MAE, microwave-assisted extraction; MA-HS-SPME, microwave-assisted headspace solid-phase microextraction; ME, matrix effect; MEPS, microextraction by packed sorbents; MLOQs, method limits of quantification; MRM, multiple-reaction monitoring; MSPD, matrix solid-phase dispersion; MTBSTFA, N-methyl-N-tert-butyldimethylsilyltrifluoacetamid; MTCS, methyl triclosan; NaBEt4, sodium tetraethyloborate; NCI, negative chemical ionization; NPEOs, nonylphenol ethoxylates; NSAIDs, nonsteroidal anti-inflammatory drugs; OPFRs, organophosphate flame-retardant plasticizers; OPs, organophosphate compounds; PBDEs, polybrominated diphenyl ethers; PCBs, polychlorinated biphenyls; PFAS, per- and polyfluoroalkyl substances; PFBAY, pentafluoro benzaldehyde; PFCs, perfluorinated compounds; PFOs, perfluorooctanesulfonic acids; PhACs, pharmaceutical active compounds; pKa, molecular weight, acid dissociation constant; PLE, pressurized liquid extraction; PP-HF, polypropylene hollow fiber; PPCPs, pharmaceuticals and personal care products; qLIT, quadrupole and linear ion trap; QqQ, triple-quadrupole; qTOF, quadrupole time-of-flight; QuEChERS, quick, easy, cheap, effective, rugged, and safe; RP-LC, reverse-phase liquid chromatography; RR-LC, rapid-resolution liquid chromatography; RSD, relative standard deviation; SALLE, salt-assisted liquid–liquid extraction; SFE, supercritical fluid extraction; SIM, selected ion monitoring; SIS, selected ion storage; SLE-LTP, solid–liquid extraction with low-temperature purification; sPLE, selective pressurized liquid extraction; SPME, solid-phase microextraction; SRM, selected reaction monitoring; SS, sewage sludge; SBSE, stir bar sorptive extraction; TBBPA, tetrabromobisphenol; TCS, triclosan; TEA, triethylamine; THPE-DMISPE, 1,1,1-Tris(4-hydroxyphenyl)ethane, molecularly imprinted solid-phase extraction; TMCS, trimethylchlorosilane; TOF, time-of-flight; UAE, ultrasound-assisted extraction; UHPLC, ultra-high-performance liquid chromatography; US EPA, United States Environmental Protection Agency; WWTPs, wastewater treatment plants.

## References

[1] Tijani J.O., Fatoba O.O., Babajide O.O., Petrik L.F. (2016). Pharmaceuticals, endocrine disruptors, personal care products, nanomaterials and perfluorinated pollutants: A review. Environ. Chem. Lett..

[2] Caliman F.A., Gavrilescu M. (2009). Pharmaceuticals, personal care products and endocrine disrupting agents in the environment–a review. CLEAN–Soil Air Water.

[3] Giulivo M., Lopez de Alda M., Capri E., Barceló D. (2016). Human exposure to endocrine disrupting compounds: Their role in reproductive systems, metabolic syndrome and breast cancer. A review. Environ. Res..

[4] Sharma T., Singh A., Kumar N., Chauhan G., Singh D.P., Singh A., Rana B.B., George N., Dwibedi V., Rath S.K., Chauhan P.S. (2023). Emerging Pollutants in the Environment and Ecological Risks. Management and Mitigation of Emerging Pollutants.

[5] (2009). Emerging Substances | NORMAN. https://www.norman-network.net/?q=node/19.

[6] Dubey M., Mohapatra S., Tyagi V.K., Suthar S., Kazmi A.A. (2021). Occurrence, fate, and persistence of emerging micropollutants in sewage sludge treatment. Environ. Pollut..

[7] Fijalkowski K., Rorat A., Grobelak A., Kacprzak M.J. (2017). The presence of contaminations in sewage sludge—The current situation. J. Environ. Manag..

[8] Govind R., Shrestha A., Govind R., Shrestha A. (2022). Sorption of Pollutants in Wastewater Solids. Sorption—From Fundamentals to Applications.

[9] Mejías C., Santos J.L., Martín J., Aparicio I., Alonso E. (2023). Multiresidue Method for the Determination of Critically and Highly Important Classes of Antibiotics and Their Metabolites in Agricultural Soils and Sewage Sludge. Anal. Bioanal. Chem..

[10] Zhou T., Li X., Liu H., Dong S., Zhang Z., Wang Z., Li J., Nghiem L.D., Khan S.J., Wang Q. (2024). Occurrence, fate, and remediation for per-and polyfluoroalkyl substances (PFAS) in sewage sludge: A comprehensive review. J. Hazard. Mater..

[11] Kacprzak M., Neczaj E., Fijałkowski K., Grobelak A., Grosser A., Worwag M., Rorat A., Brattebo H., Almås Å., Singh B.R. (2017). Sewage sludge disposal strategies for sustainable development. Environ. Res..

[12] Hudcová H., Vymazal J., Rozkošný M. (2019). Present restrictions of sewage sludge application in agriculture within the European Union. Soil Water Res..

[13] Buta M., Hubeny J., Zieliński W., Harnisz M., Korzeniewska E. (2021). Sewage sludge in agriculture—The effects of selected chemical pollutants and emerging genetic resistance determinants on the quality of soil and crops—A review. Ecotoxicol. Environ. Saf..

[14] Decision (EU) 2018/840 Decision (EU) 2018/840 of 5 June 2018 Establishing a Watch List of Substances for Union-Wide Monitoring in the Field of Water Policy Pursuant to Directive 2008/105/EC of the European Parliament and of the Council and Repealing Commission Implementing Decision (EU) 2015/495 (Notified Under Document C(2018) 3362). https://eur-lex.europa.eu/eli/dec_impl/2018/840/oj.

[15] Lindholm-Lehto P.C., Ahkola H.S.J., Knuutinen J.S. (2017). Procedures of determining organic trace compounds in municipal sewage sludge—A review. Environ. Sci. Pollut. Res..

[16] Wilkinson J.L., Hooda P.S., Swinden J., Barker J., Barton S. (2017). Spatial distribution of organic contaminants in three rivers of Southern England bound to suspended particulate material and dissolved in water. Sci. Total Environ..

[17] Chen Q., Shi J., Wu W., Liu X., Zhang H. (2012). A new pretreatment and improved method for determination of selected estrogens in high matrix solid sewage samples by liquid chromatography mass spectrometry. Microchem. J..

[18] Lee H.-B., Lewina Svoboda M., Peart T.E., Smyth S.A. (2016). Optimization of a microwave-assisted extraction procedure for the determination of selected alkyl, aryl, and halogenated phenols in sewage sludge and biosolids. Water Qual. Res. J..

[19] Gabet-Giraud V., Miege C., Herbreteau B., Hernandez-Raquet G., Coquery M. (2010). Development and validation of an analytical method by LC-MS/MS for the quantification of estrogens in sewage sludge. Anal. Bioanal. Chem..

[20] Yu Y., Huang Q., Cui J., Zhang K., Tang C., Peng X. (2011). Determination of pharmaceuticals, steroid hormones, and endocrine-disrupting personal care products in sewage sludge by ultra-high-performance liquid chromatography-tandem mass spectrometry. Anal. Bioanal. Chem..

[21] Demirtepe H., Imamoglu I. (2019). Levels of polybrominated diphenyl ethers and hexabromocyclododecane in treatment plant sludge: Implications on sludge management. Chemosphere.

[22] Liu S., Ying G.-G., Zhao J.-L., Chen F., Yang B., Zhou L.-J., Lai H. (2011). Trace analysis of 28 steroids in surface water, wastewater and sludge samples by rapid resolution liquid chromatography–electrospray ionization tandem mass spectrometry. J. Chromatogr. A.

[23] Lindholm-Lehto P.C., Ahkola H.S.J., Knuutinen J.S. (2018). Pharmaceuticals in processing of municipal sewage sludge studied by grab and passive sampling. Water Qual. Res. J..

[24] Banihashemi B., Droste R.L. (2013). Trace level determination of bisphenol-A in wastewater and sewage sludge by high-performance liquid chromatography and UV detection. Water Qual. Res. J..

[25] Liu S.-S., Ying G.-G., Liu S., Lai H.-J., Chen Z.-F., Pan C.-G., Zhao J.-L., Chen J. (2014). Analysis of 21 progestagens in various matrices by ultra-high-performance liquid chromatography tandem mass spectrometry (UHPLC-MS/MS) with diverse sample pretreatment. Anal. Bioanal. Chem..

[26] Zuliani T., Milačič R., Ščančar J. (2012). Preparation of a sewage sludge laboratory quality control material for butyltin compounds and their determination by isotope-dilution mass spectrometry. Anal. Bioanal. Chem..

[27] Llop A., Borrull F., Pocurull E. (2010). Pressurised hot water extraction followed by simultaneous derivatization and headspace solid-phase microextraction and gas chromatography-tandem mass spectrometry for the determination of aliphatic primary amines in sewage sludge. Anal. Chim. Acta.

[28] Vallecillos L., Borrull F., Pocurull E. (2012). Determination of musk fragrances in sewage sludge by pressurized liquid extraction coupled to automated ionic liquid-based headspace single-drop microextraction followed by GC-MS/MS. J. Sep. Sci..

[29] Herrero P., Borrull F., Pocurull E., Marcé R.M. (2014). A quick, easy, cheap, effective, rugged and safe extraction method followed by liquid chromatography-(Orbitrap) high resolution mass spectrometry to determine benzotriazole, benzothiazole and benzenesulfonamide derivates in sewage sludge. J. Chromatogr. A.

[30] Bergé A., Buleté A., Fildier A., Vulliet E. (2017). High-Resolution Mass Spectrometry as a Tool To Evaluate the Sample Preparation of Sludge. Anal. Chem..

[31] Ferhi S., Bourdat-Deschamps M., Daudin J.-J., Houot S., Nélieu S. (2016). Factors influencing the extraction of pharmaceuticals from sewage sludge and soil: An experimental design approach. Anal. Bioanal. Chem..

[32] Soares K.L., Cerqueira M.B.R., Caldas S.S., Primel E.G. (2017). Evaluation of alternative environmentally friendly matrix solid phase dispersion solid supports for the simultaneous extraction of 15 pesticides of different chemical classes from drinking water treatment sludge. Chemosphere.

[33] Navarro I., Sanz P., Martínez M.Á. (2011). Analysis of perfluorinated alkyl substances in Spanish sewage sludge by liquid chromatography–tandem mass spectrometry. Anal. Bioanal. Chem..

[34] Saleh A., Larsson E., Yamini Y., Jönsson J.Å. (2011). Hollow fiber liquid phase microextraction as a preconcentration and clean-up step after pressurized hot water extraction for the determination of non-steroidal anti-inflammatory drugs in sewage sludge. J. Chromatogr. A.

[35] Samaras V.G., Thomaidis N.S., Stasinakis A.S., Lekkas T.D. (2011). An analytical method for the simultaneous trace determination of acidic pharmaceuticals and phenolic endocrine disrupting chemicals in wastewater and sewage sludge by gas chromatography-mass spectrometry. Anal. Bioanal. Chem..

[36] Dorival-García N., Zafra-Gómez A., Camino-Sánchez F.J., Navalón A., Vílchez J.L. (2013). Analysis of quinolone antibiotic derivatives in sewage sludge samples by liquid chromatography–tandem mass spectrometry: Comparison of the efficiency of three extraction techniques. Talanta.

[37] Yu Y., Wu L. (2012). Analysis of endocrine disrupting compounds, pharmaceuticals and personal care products in sewage sludge by gas chromatography–mass spectrometry. Talanta.

[38] Manso J., Larsson E., Jönsson J.Å. (2014). Determination of 4′-isobutylacetophenone and other transformation products of anti-inflammatory drugs in water and sludge from five wastewater treatment plants in Sweden by hollow fiber liquid phase microextraction and gas chromatography–mass spectrometry. Talanta.

[39] Sagristà E., Larsson E., Ezoddin M., Hidalgo M., Salvadó V., Jönsson J.Å. (2010). Determination of non-steroidal anti-inflammatory drugs in sewage sludge by direct hollow fiber supported liquid membrane extraction and liquid chromatography–mass spectrometry. J. Chromatogr. A.

[40] Martínez-Moral M.P., Tena M.T. (2014). Use of microextraction by packed sorbents following selective pressurised liquid extraction for the determination of brominated diphenyl ethers in sewage sludge by gas chromatography–mass spectrometry. J. Chromatogr. A.

[41] Viglino L., Prévost M., Sauvé S. (2011). High throughput analysis of solid-bound endocrine disruptors by LDTD-APCI-MS/MS. J. Environ. Monit..

[42] Zhao M., Yao Y., Dong X., Fang B., Wang Z., Chen H., Sun H. (2025). Identification of emerging PFAS in industrial sludge from North China: Release risk assessment by the TOP assay. Water Res..

[43] Angeles-de Paz G., León-Morcillo R., Guzmán S., Robledo-Mahón T., Pozo C., Calvo C., Aranda E. (2023). Pharmaceutical active compounds in sewage sludge: Degradation improvement and conversion into an organic amendment by bioaugmentation-composting processes. Waste Manag..

[44] Angeles-De Paz G., Cubero-Cardoso J., Pozo C., Calvo C., Aranda E., Robledo-Mahón T. (2025). Optimizing Bioaugmentation for Pharmaceutical Stabilization of Sewage Sludge: A Study on Short-Term Composting Under Real Conditions. J. Fungi.

[45] Wilschnack K., Homer B., Cartmell E., Yates K., Petrie B. (2024). Targeted multi-analyte UHPLC-MS/MS methodology for emerging contaminants in septic tank wastewater, sludge and receiving surface water. Anal. Methods.

[46] Miserli K., Kosma C., Konstantinou I. (2023). Determination of pharmaceuticals and metabolites in sludge and hydrochar after hydrothermal carbonization using sonication—QuEChERS extraction method and UHPLC LTQ/Orbitrap MS. Environ. Sci. Pollut. Res..

[47] Zhang Y., Zhao B., Chen Q., Zhu F., Wang J., Fu X., Zhou T. (2023). Fate of organophosphate flame retardants (OPFRs) in the “Cambi® TH + AAD” of sludge in a WWTP in Beijing, China. Waste Manag..

[48] Zuloaga O., Navarro P., Bizkarguenaga E., Iparraguirre A., Vallejo A., Olivares M., Prieto A. (2012). Overview of extraction, clean-up and detection techniques for the determination of organic pollutants in sewage sludge: A review. Anal. Chim. Acta.

[49] Luque de Castro M.D., Priego-Capote F. (2010). Soxhlet extraction: Past and present panacea. J. Chromatogr. A.

[50] Veenaas C., Haglund P. (2017). Methodology for non-target screening of sewage sludge using comprehensive two-dimensional gas chromatography coupled to high-resolution mass spectrometry. Anal. Bioanal. Chem..

[51] Pérez-Lemus N., López-Serna R., Pérez-Elvira S.I., Barrado E. (2019). Analytical methodologies for the determination of pharmaceuticals and personal care products (PPCPs) in sewage sludge: A critical review. Anal. Chim. Acta.

[52] Chen Y., Cao Q., Deng S., Huang J., Wang B., Yu G. (2013). Determination of pharmaceuticals from various therapeutic classes in dewatered sludge by pressurized liquid extraction and high performance liquid chromatography and tandem mass spectrometry (HPLC-MS/MS). Int. J. Environ. Anal. Chem..

[53] Castro G., Carpinteiro I., Rodríguez I., Cela R. (2018). Determination of cardiovascular drugs in sewage sludge by matrix solid-phase dispersion and ultra-performance liquid chromatography tandem mass spectrometry. Anal. Bioanal. Chem..

[54] Novak P., Zuliani T., Milačič R., Ščančar J. (2016). Development of an analytical method for the determination of polybrominated diphenyl ethers in sewage sludge by the use of gas chromatography coupled to inductively coupled plasma mass spectrometry. Anal. Chim. Acta.

[55] Abril C., Santos J.L., Malvar J.L., Martín J., Aparicio I., Alonso E. (2018). Determination of perfluorinated compounds, bisphenol A, anionic surfactants and personal care products in digested sludge, compost and soil by liquid-chromatography-tandem mass spectrometry. J. Chromatogr. A.

[56] Yang Y., Lu L., Zhang J., Yang Y., Wu Y., Shao B. (2014). Simultaneous determination of seven bisphenols in environmental water and solid samples by liquid chromatography–electrospray tandem mass spectrometry. J. Chromatogr. A.

[57] Huang Q., Yu Y., Tang C., Peng X. (2010). Determination of commonly used azole antifungals in various waters and sewage sludge using ultra-high performance liquid chromatography-tandem mass spectrometry. J. Chromatogr. A.

[58] Garcia-Rodríguez A., Sagristà E., Matamoros V., Fontàs C., Hidalgo M., Salvadó V. (2014). Determination of pharmaceutical compounds in sewage sludge using a standard addition method approach. Int. J. Environ. Anal. Chem..

[59] Ohoro C.R., Adeniji A.O., Okoh A.I., Okoh A.O.O. (2019). Distribution and Chemical Analysis of Pharmaceuticals and Personal Care Products (PPCPs) in the Environmental Systems: A Review. Int J Environ. Res Public Health.

[60] Martín-Pozo L., de Alarcón-Gómez B., Rodríguez-Gómez R., García-Córcoles M.T., Çipa M., Zafra-Gómez A. (2019). Analytical methods for the determination of emerging contaminants in sewage sludge samples. A review. Talanta.

[61] López-Serna R., Marín-de-Jesús D., Irusta-Mata R., García-Encina P.A., Lebrero R., Fdez-Polanco M., Muñoz R. (2018). Multiresidue analytical method for pharmaceuticals and personal care products in sewage and sewage sludge by online direct immersion SPME on-fiber derivatization–GCMS. Talanta.

[62] Llop A., Borrull F., Pocurull E. (2012). Pressurised hot water extraction followed by headspace solid-phase microextraction and gas chromatography-tandem mass spectrometry for the determination of N-nitrosamines in sewage sludge. Talanta.

[63] Gao D., Li Z., Wen Z., Ren N. (2014). Occurrence and fate of phthalate esters in full-scale domestic wastewater treatment plants and their impact on receiving waters along the Songhua River in China. Chemosphere.

[64] Gani K.M., Kazmi A.A. (2016). Comparative assessment of phthalate removal and risk in biological wastewater treatment systems of developing countries and small communities. Sci. Total Environ..

[65] Gani K.M., Bux F., Kazmi A.A. (2019). Diethylhexyl phthalate removal in full scale activated sludge plants: Effect of operational parameters. Chemosphere.

[66] Zhang Z., Ren N., Li Y.-F., Kunisue T., Gao D., Kannan K. (2011). Determination of benzotriazole and benzophenone UV filters in sediment and sewage sludge. Environ. Sci. Technol..

[67] Gani K.M., Kazmi A.A. (2020). Ecotoxicological risk evaluation and regulatory compliance of endocrine disruptor phthalates in a sustainable wastewater treatment scheme. Enviorn. Sci. Pollut. Res..

[68] Qian Y., Jia X., Ding T., Yang M., Yang B., Li J. (2021). Occurrence and removal of bisphenol analogues in wastewater treatment plants and activated sludge bioreactor. Sci. Total Environ..

[69] Eljarrat E., Barceló D. (2003). Priority lists for persistent organic pollutants and emerging contaminants based on their relative toxic potency in environmental samples. TrAC Trends Anal. Chem..

[70] Gao S., Tian B., Zeng X., Yu Z. (2019). Enantiomeric analysis of polycyclic musks AHTN and HHCB and HHCB-lactone in sewage sludge by gas chromatography/tandem mass spectrometry. Rapid Commun. Mass Spectrom..

[71] Vrkoslavová J., Demnerová K., Macková M., Zemanová T., Macek T., Hajšlová J., Pulkrabová J., Hrádková P., Stiborová H. (2010). Absorption and translocation of polybrominated diphenyl ethers (PBDEs) by plants from contaminated sewage sludge. Chemosphere.

[72] Cincinelli A., Martellini T., Misuri L., Lanciotti E., Sweetman A., Laschi S., Palchetti I. (2012). PBDEs in Italian sewage sludge and environmental risk of using sewage sludge for land application. Environ. Pollut..

[73] Zeng X.-Y., Cao S.-X., Zhang D.-L., Gao S.-T., Yu Z.-Q., Li H.-R., Sheng G.-Y., Fu J.-M. (2012). Levels and distribution of synthetic musks and polycyclic aromatic hydrocarbons in sludge collected from Guangdong Province. J. Environ. Sci Health A.

[74] Luque-García J.L., Luque de Castro M.D. (2003). Ultrasound: A powerful tool for leaching. TrAC Trends Anal. Chem..

[75] Tadeo J.L., Sánchez-Brunete C., Albero B., García-Valcárcel A.I. (2010). Application of ultrasound-assisted extraction to the determination of contaminants in food and soil samples. J. Chromatogr. A.

[76] Albero B., Sánchez-Brunete C., García-Valcárcel A.I., Pérez R.A., Tadeo J.L. (2015). Ultrasound-assisted extraction of emerging contaminants from environmental samples. TrAC Trends Anal. Chem..

[77] Guerra P., Eljarrat E., Barceló D. (2010). Simultaneous determination of hexabromocyclododecane, tetrabromobisphenol A, and related compounds in sewage sludge and sediment samples from Ebro River basin (Spain). Anal. Bioanal. Chem..

[78] Cheriyan B.V., Karunakar K.K., Anandakumar R., Murugathirumal A., Kumar A.S. (2025). Eco-friendly extraction technologies: A comprehensive review of modern green analytical methods. Sustain. Chem. Clim. Action.

[79] Gago-Ferrero P., Borova V., Dasenaki M.E., Τhomaidis Ν.S. (2015). Simultaneous determination of 148 pharmaceuticals and illicit drugs in sewage sludge based on ultrasound-assisted extraction and liquid chromatography–tandem mass spectrometry. Anal. Bioanal. Chem..

[80] Zhang M., Mao Q., Feng J., Yuan S., Wang Q., Huang D., Zhang J. (2016). Validation and application of an analytical method for the determination of selected acidic pharmaceuticals and estrogenic hormones in wastewater and sludge. J. Environ. Sci. Health Part A.

[81] Chen Z.-F., Ying G.-G., Lai H.-J., Chen F., Su H.-C., Liu Y.-S., Peng F.-Q., Zhao J.-L. (2012). Determination of biocides in different environmental matrices by use of ultra-high-performance liquid chromatography–tandem mass spectrometry. Anal. Bioanal. Chem..

[82] Huang Q., Zhang K., Wang Z., Wang C., Peng X. (2012). Enantiomeric determination of azole antifungals in wastewater and sludge by liquid chromatography–tandem mass spectrometry. Anal. Bioanal. Chem..

[83] Martínez-Moral M.P., Tena M.T. (2011). Focused ultrasound solid–liquid extraction and selective pressurised liquid extraction to determine bisphenol A and alkylphenols in sewage sludge by gas chromatography–mass spectrometry. J. Sep. Sci..

[84] Martínez-Moral M.P., Tena M.T. (2013). Focused ultrasound solid–liquid extraction of perfluorinated compounds from sewage sludge. Talanta.

[85] Cristale J., Lacorte S. (2013). Development and validation of a multiresidue method for the analysis of polybrominated diphenyl ethers, new brominated and organophosphorus flame retardants in sediment, sludge and dust. J. Chromatogr. A.

[86] Fernández-Sanjuan M., Lacorte S., Rigol A., Sahuquillo A. (2012). New quality-control materials for the determination of alkylphenols and alkylphenol ethoxylates in sewage sludge. Anal. Bioanal. Chem..

[87] Koumaki E., Noutsopoulos C., Mamais D., Fragkiskatos G., Andreadakis A. (2021). Fate of Emerging Contaminants in High-Rate Activated Sludge Systems. Int. J. Environ. Res. Public Health.

[88] Golovko O., Örn S., Sörengård M., Frieberg K., Nassazzi W., Lai F.Y., Ahrens L. (2021). Occurrence and removal of chemicals of emerging concern in wastewater treatment plants and their impact on receiving water systems. Sci. Total Environ..

[89] Meng Y., Liu W., Fiedler H., Zhang J., Wei X., Liu X., Peng M., Zhang T. (2021). Fate and risk assessment of emerging contaminants in reclaimed water production processes. Front. Environ. Sci. Eng..

[90] Santana J.M., Fraga S.V.B., Zanatta M.C.K., Martins M.R., Pires M.S.G. (2021). Characterization of organic compounds and drugs in sewage sludge aiming for agricultural recycling. Heliyon.

[91] Álvarez-Ruiz R., Andrés-Costa M.J., Andreu V., Picó Y. (2015). Simultaneous determination of traditional and emerging illicit drugs in sediments, sludges and particulate matter. J. Chromatogr. A.

[92] Košnář Z., Mercl F., Pierdonà L., Chane A.D., Míchal P., Tlustoš P. (2023). Concentration of the main persistent organic pollutants in sewage sludge in relation to wastewater treatment plant parameters and sludge stabilisation. Environ. Pollut..

[93] Liu R., Ruan T., Wang T., Song S., Yu M., Gao Y., Shao J., Jiang G. (2013). Trace analysis of mono-, di-, tri-substituted polyfluoroalkyl phosphates and perfluorinated phosphonic acids in sewage sludge by high performance liquid chromatography tandem mass spectrometry. Talanta.

[94] Chokwe T.B., Okonkwo J.O., Sibali L.L., Ncube E.J. (2015). An integrated method for the simultaneous determination of alkylphenol ethoxylates and brominated flame retardants in sewage sludge samples by ultrasonic-assisted extraction, solid phase clean-up, and GC-MS analysis. Microchem. J..

[95] Zacs D., Bartkevics V. (2016). Trace determination of perfluorooctane sulfonate and perfluorooctanoic acid in environmental samples (surface water, wastewater, biota, sediments, and sewage sludge) using liquid chromatography–Orbitrap mass spectrometry. J. Chromatogr. A.

[96] Clara M., Gans O., Windhofer G., Krenn U., Hartl W., Braun K., Scharf S., Scheffknecht C. (2011). Occurrence of polycyclic musks in wastewater and receiving water bodies and fate during wastewater treatment. Chemosphere.

[97] Healy M.G., Fenton O., Cormican M., Peyton D.P., Ordsmith N., Kimber K., Morrison L. (2017). Antimicrobial compounds (triclosan and triclocarban) in sewage sludges, and their presence in runoff following land application. Ecotoxicol. Environ. Saf..

[98] Zhou G.-J., Li X.-Y., Leung K.M.Y. (2019). Retinoids and oestrogenic endocrine disrupting chemicals in saline sewage treatment plants: Removal efficiencies and ecological risks to marine organisms. Environ. Int..

[99] Zhou G.-J., Lin L., Li X.-Y., Leung K.M.Y. (2020). Removal of emerging contaminants from wastewater during chemically enhanced primary sedimentation and acidogenic sludge fermentation. Water Res..

[100] Kor-Bicakci G., Abbott T., Ubay-Cokgor E., Eskicioglu C. (2020). Occurrence of the Persistent Antimicrobial Triclosan in Microwave Pretreated and Anaerobically Digested Municipal Sludges under Various Process Conditions. Molecules.

[101] Tasselli S., Guzzella L. (2020). Polycyclic musk fragrances (PMFs) in wastewater and activated sludge: Analytical protocol and application to a real case study. Environ. Sci. Pollut. Res..

[102] Wang Y., Teng Y., Wang D., Han K., Wang H., Kang L. (2020). The fate of triclocarban in activated sludge and its influence on biological wastewater treatment system. J. Environ. Manag..

[103] Abbott T., Eskicioglu C. (2020). Comparison of anaerobic, cycling aerobic/anoxic, and sequential anaerobic/aerobic/anoxic digestion to remove triclosan and triclosan metabolites from municipal biosolids. Sci. Total Environ..

[104] Košnář Z., Mercl F., Chane A.D., Pierdonà L., Míchal P., Tlustoš P. (2021). Occurrence of synthetic polycyclic and nitro musk compounds in sewage sludge from municipal wastewater treatment plants. Sci. Total Environ..

[105] Martín J., Santos J.L., Aparicio I., Alonso E. (2010). Multi-residue method for the analysis of pharmaceutical compounds in sewage sludge, compost and sediments by sonication-assisted extraction and LC determination. J. Sep. Sci..

[106] Lajeunesse A., Smyth S.A., Barclay K., Sauvé S., Gagnon C. (2012). Distribution of antidepressant residues in wastewater and biosolids following different treatment processes by municipal wastewater treatment plants in Canada. Water Res..

[107] Shafrir M., Avisar D. (2012). Development Method for Extracting and Analyzing Antibiotic and Hormone Residues from Treated Wastewater Sludge and Composted Biosolids. Water Air Soil Pollut..

[108] Zhou L.-J., Ying G.-G., Liu S., Zhao J.-L., Chen F., Zhang R.-Q., Peng F.-Q., Zhang Q.-Q. (2012). Simultaneous determination of human and veterinary antibiotics in various environmental matrices by rapid resolution liquid chromatography–electrospray ionization tandem mass spectrometry. J. Chromatogr. A.

[109] Yuan X., Qiang Z., Ben W., Zhu B., Liu J. (2014). Rapid detection of multiple class pharmaceuticals in both municipal wastewater and sludge with ultra high performance liquid chromatography tandem mass spectrometry. J. Environ. Sci..

[110] Boix C., Ibáñez M., Fabregat-Safont D., Morales E., Pastor L., Sancho J.V., Sánchez-Ramírez J.E., Hernández F. (2016). Behaviour of emerging contaminants in sewage sludge after anaerobic digestion. Chemosphere.

[111] Boix C., Ibáñez M., Fabregat-Safont D., Morales E., Pastor L., Sancho J.V., Sánchez-Ramírez J.E., Hernández F. (2016). Analytical methodologies based on LC–MS/MS for monitoring selected emerging compounds in liquid and solid phases of the sewage sludge. MethodsX.

[112] Martínez-Alcalá I., Guillén-Navarro J.M., Fernández-López C. (2017). Pharmaceutical biological degradation, sorption and mass balance determination in a conventional activated-sludge wastewater treatment plant from Murcia, Spain. Chem. Eng. J..

[113] Abbott T., Kor-Bicakci G., Eskicioglu C. (2021). Examination of single-stage anaerobic and anoxic/aerobic and dual-stage anaerobic-anoxic/aerobic digestion to remove pharmaceuticals from municipal biosolids. Sci. Total Environ..

[114] Martínez-Alcalá I., Guillén-Navarro J.M., Lahora A. (2021). Occurrence and fate of pharmaceuticals in a wastewater treatment plant from southeast of Spain and risk assessment. J. Environ. Manag..

[115] Mercl F., Košnář Z., Maršík P., Vojtíšek M., Dušek J., Száková J., Tlustoš P. (2021). Pyrolysis of biosolids as an effective tool to reduce the uptake of pharmaceuticals by plants. J. Hazard. Mater..

[116] Ömeroğlu S., Kara Murdoch F., Dilek Sanin F. (2015). Investigation of nonylphenol and nonylphenol ethoxylates in sewage sludge samples from a metropolitan wastewater treatment plant in Turkey. Talanta.

[117] Sun X., Peng J., Wang M., Wang J., Tang C., Yang L., Lei H., Li F., Wang X., Chen J. (2018). Determination of nine bisphenols in sewage and sludge using dummy molecularly imprinted solid-phase extraction coupled with liquid chromatography tandem mass spectrometry. J. Chromatogr. A.

[118] García-Valcárcel A.I., Tadeo J.L. (2011). Determination of azoles in sewage sludge from Spanish wastewater treatment plants by liquid chromatography-tandem mass spectrometry. J. Sep. Sci..

[119] Tang C., Yu Y., Huang Q., Peng X. (2012). Simultaneous determination of fluoroquinolone and tetracycline antibacterials in sewage sludge using ultrasonic-assisted extraction and HPLC-MS/MS. Int. J. Environ. Anal. Chem..

[120] Hajj-Mohamad M., Aboulfadl K., Darwano H., Madoux-Humery A.-S., Guérineau H., Sauvé S., Prévost M., Dorner S. (2014). Wastewater micropollutants as tracers of sewage contamination: Analysis of combined sewer overflow and stream sediments. Environ. Sci. Process. Impacts.

[121] Malvar J.L., Santos J.L., Martín J., Aparicio I., Alonso E. (2020). Comparison of ultrasound-assisted extraction, QuEChERS and selective pressurized liquid extraction for the determination of metabolites of parabens and pharmaceuticals in sludge. Microchem. J..

[122] Method 1694: Pharmaceuticals and Personal Care Products in Water, Soil, Sediment, and Biosolids by HPLC/MS/MS. 2007, 77. https://www.epa.gov/sites/default/files/2015-10/documents/method_1694_2007.pdf.

[123] US EPA (2000). Method 3550C–Ultrasonic Extraction.

[124] Azzouz A., Ballesteros E. (2016). Determination of 13 endocrine disrupting chemicals in environmental solid samples using microwave-assisted solvent extraction and continuous solid-phase extraction followed by gas chromatography–mass spectrometry. Anal. Bioanal. Chem..

[125] Guedes-Alonso R., Santana-Viera S., Montesdeoca-Esponda S., Afonso-Olivares C., Sosa-Ferrera Z., Santana-Rodríguez J.J. (2016). Application of microwave-assisted extraction and ultra-high performance liquid chromatography–tandem mass spectrometry for the analysis of sex hormones and corticosteroids in sewage sludge samples. Anal. Bioanal. Chem..

[126] Petrie B., Youdan J., Barden R., Kasprzyk-Hordern B. (2016). Multi-residue analysis of 90 emerging contaminants in liquid and solid environmental matrices by ultra-high-performance liquid chromatography tandem mass spectrometry. J. Chromatogr. A.

[127] Vega-Morales T., Sosa-Ferrera Z., Santana-Rodríguez J.J. (2011). Determination of various estradiol mimicking-compounds in sewage sludge by the combination of microwave-assisted extraction and LC–MS/MS. Talanta.

[128] Cantarero S., Zafra-Gómez A., Ballesteros O., Navalón A., Vílchez J.L., Verge C., De Ferrer J.A. (2011). Matrix effect study in the determination of linear alkylbenzene sulfonates in sewage sludge samples. Environ. Toxicol. Chem..

[129] Dobor J., Varga M., Yao J., Chen H., Palkó G., Záray G. (2010). A new sample preparation method for determination of acidic drugs in sewage sludge applying microwave assisted solvent extraction followed by gas chromatography–mass spectrometry. Microchem. J..

[130] Montesdeoca-Esponda S., Sosa-Ferrera Z., Santana-Rodríguez J.J. (2012). Combination of microwave-assisted micellar extraction with liquid chromatography tandem mass spectrometry for the determination of fluoroquinolone antibiotics in coastal marine sediments and sewage sludges samples. Biomed. Chromatogr..

[131] Devault D.A., Amalric L., Bristeau S., Cruz J., Tapie N., Karolak S., Budzinski H., Lévi Y. (2021). Removal efficiency of emerging micropollutants in biofilter wastewater treatment plants in tropical areas. Environ. Sci. Pollut. Res. Int..

[132] Evans S.E., Davies P., Lubben A., Kasprzyk-Hordern B. (2015). Determination of chiral pharmaceuticals and illicit drugs in wastewater and sludge using microwave assisted extraction, solid-phase extraction and chiral liquid chromatography coupled with tandem mass spectrometry. Anal. Chim. Acta.

[133] Junior I.L.C., Machado C.S., Pletsch A.L., Torres Y.R. (2020). Simultaneous HPLC-PDA determination of commonly prescribed antidepressants and caffeine in sludge from sewage treatment plants and river sediments in the Itaipu reservoir region, Paraná, Brazil. Int. J. Environ. Anal. Chem..

[134] Junior I.L.C., Machado C.S., Ramalho A.N., Pletsch A.L., Torres Y.R. (2017). Optimisation of caffeine and antidepressants extraction from sediments and sewage sludge using experimental designs. Int. J. Environ. Anal. Chem..

[135] Zhou S., Peng S., Li Z., Zhang D., Zhu Y., Li X., Hong M., Li W., Lu P. (2022). Risk assessment of pollutants in flowback and produced waters and sludge in impoundments. Sci. Total Environ..

[136] Azzouz A., Ballesteros E. (2012). Combined microwave-assisted extraction and continuous solid-phase extraction prior to gas chromatography–mass spectrometry determination of pharmaceuticals, personal care products and hormones in soils, sediments and sludge. Sci. Total Environ..

[137] Rhodes L. (2002). Microwave-assisted extraction using US EPA method 3546. LC GC N. Am..

[138] Anastassiades M., Lehotay S.J., Stajnbaher D., Schenck F.J. (2003). Fast and easy multiresidue method employing acetonitrile extraction/partitioning and “dispersive solid-phase extraction” for the determination of pesticide residues in produce. J. AOAC Int..

[139] Arbeláez P., Borrull F., Maria Marcé R., Pocurull E. (2015). Trace-level determination of sweeteners in sewage sludge using selective pressurized liquid extraction and liquid chromatography–tandem mass spectrometry. J. Chromatogr. A.

[140] García-Galán M.J., Díaz-Cruz S., Barceló D. (2013). Multiresidue trace analysis of sulfonamide antibiotics and their metabolites in soils and sewage sludge by pressurized liquid extraction followed by liquid chromatography–electrospray-quadrupole linear ion trap mass spectrometry. J. Chromatogr. A.

[141] Mohapatra D.P., Brar S.K., Tyagi R.D., Picard P., Surampalli R.Y. (2012). Carbamazepine in municipal wastewater and wastewater sludge: Ultrafast quantification by laser diode thermal desorption-atmospheric pressure chemical ionization coupled with tandem mass spectrometry. Talanta.

[142] Radjenović J., Petrović M., Barceló D. (2009). Fate and distribution of pharmaceuticals in wastewater and sewage sludge of the conventional activated sludge (CAS) and advanced membrane bioreactor (MBR) treatment. Water Res..

[143] Radjenović J., Jelić A., Petrović M., Barceló D. (2009). Determination of pharmaceuticals in sewage sludge by pressurized liquid extraction (PLE) coupled to liquid chromatography-tandem mass spectrometry (LC-MS/MS). Anal. Bioanal. Chem..

[144] Salvia M.-V., Fieu M., Vulliet E. (2015). Determination of Tetracycline and Fluoroquinolone Antibiotics at Trace Levels in Sludge and Soil. Appl. Environ. Soil Sci..

[145] Scheurer M., Ramil M., Metcalfe C.D., Groh S., Ternes T.A. (2010). The challenge of analyzing beta-blocker drugs in sludge and wastewater. Anal. Bioanal. Chem..

[146] Seira J., Claparols C., Joannis-Cassan C., Albasi C., Montréjaud-Vignoles M., Sablayrolles C. (2013). Optimization of pressurized liquid extraction using a multivariate chemometric approach for the determination of anticancer drugs in sludge by ultra high performance liquid chromatography–tandem mass spectrometry. J. Chromatogr. A.

[147] Esparza X., Moyano E., de Boer J., Galceran M.T., van Leeuwen S.P.J. (2011). Analysis of perfluorinated phosponic acids and perfluorooctane sulfonic acid in water, sludge and sediment by LC–MS/MS. Talanta.

[148] Gorga M., Insa S., Petrovic M., Barceló D. (2014). Analysis of endocrine disrupters and related compounds in sediments and sewage sludge using on-line turbulent flow chromatography–liquid chromatography–tandem mass spectrometry. J. Chromatogr. A.

[149] Mascolo G., Locaputo V., Mininni G. (2010). New perspective on the determination of flame retardants in sewage sludge by using ultrahigh pressure liquid chromatography-tandem mass spectrometry with different ion sources. J Chromatogr A.

[150] Mastroianni N., Postigo C., de Alda M.L., Barcelo D. (2013). Illicit and abused drugs in sewage sludge: Method optimization and occurrence. J. Chromatogr. A.

[151] Tohidi F., Cai Z. (2015). GC/MS analysis of triclosan and its degradation by-products in wastewater and sludge samples from different treatments. Environ. Sci. Pollut. Res..

[152] Riva F., Zuccato E., Pacciani C., Colombo A., Castiglioni S. (2021). A multi-residue analytical method for extraction and analysis of pharmaceuticals and other selected emerging contaminants in sewage sludge. Anal. Methods.

[153] De la Torre A., Concejero M.A., Martínez M.A. (2012). Concentrations and sources of an emerging pollutant, decabromodiphenylethane (DBDPE), in sewage sludge for land application. J. Environ. Sci..

[154] Llorca M., Farré M., Picó Y., Barceló D. (2011). Analysis of perfluorinated compounds in sewage sludge by pressurized solvent extraction followed by liquid chromatography–mass spectrometry. J. Chromatogr. A.

[155] Herrero P., Borrull F., Marcé R.M., Pocurull E. (2013). Pressurised liquid extraction and ultra-high performance liquid chromatography-tandem mass spectrometry to determine endogenous and synthetic glucocorticoids in sewage sludge. Talanta.

[156] Arbeláez P., Granados J., Borrull F., Marcé R.M., Pocurull E. (2014). Determination of sedative hypnotics in sewage sludge by pressurized liquid extraction with high-performance liquid chromatography and tandem mass spectrometry. J. Sep. Sci..

[157] vom Eyser C., Palmu K., Otterpohl R., Schmidt T.C., Tuerk J. (2015). Determination of pharmaceuticals in sewage sludge and biochar from hydrothermal carbonization using different quantification approaches and matrix effect studies. Anal. Bioanal. Chem..

[158] Lonappan L., Pulicharla R., Rouissi T., Brar S.K., Verma M., Surampalli R.Y., Valero J.R. (2016). Diclofenac in municipal wastewater treatment plant: Quantification using laser diode thermal desorption--atmospheric pressure chemical ionization--tandem mass spectrometry approach in comparison with an established liquid chromatography-electrospray ionization-tandem mass spectrometry method. J. Chromatogr. A.

[159] Langford K.H., Reid M., Thomas K.V. (2011). Multi-residue screening of prioritised human pharmaceuticals, illicit drugs and bactericides in sediments and sludge. J. Environ. Monit..

[160] Mailler R., Gasperi J., Patureau D., Vulliet E., Delgenes N., Danel A., Deshayes S., Eudes V., Guerin S., Moilleron R. (2017). Fate of emerging and priority micropollutants during the sewage sludge treatment: Case study of Paris conurbation. Part 1: Contamination of the different types of sewage sludge. Waste Manag..

[161] Shukla R., Ahammad S.Z. (2023). Performance assessment of a modified trickling filter and conventional activated sludge process along with tertiary treatment in removing emerging pollutants from urban sewage. Sci. Total Environ..

[162] Hawthorne S.B., Yang Y., Miller D.J. (1994). Extraction of Organic Pollutants from Environmental Solids with Sub- and Supercritical Water. Anal. Chem..

[163] Svahn O., Björklund E. (2019). Extraction Efficiency of a Commercial Espresso Machine Compared to a Stainless-Steel Column Pressurized Hot Water Extraction (PHWE) System for the Determination of 23 Pharmaceuticals, Antibiotics and Hormones in Sewage Sludge. Appl. Sci..

[164] Herrero P., Borrull F., Marcé R.M., Pocurull E. (2014). A pressurised hot water extraction and liquid chromatography–high resolution mass spectrometry method to determine polar benzotriazole, benzothiazole and benzenesulfonamide derivates in sewage sludge. J. Chromatogr. A.

[165] Peysson W., Vulliet E. (2013). Determination of 136 pharmaceuticals and hormones in sewage sludge using quick, easy, cheap, effective, rugged and safe extraction followed by analysis with liquid chromatography–time-of-flight-mass spectrometry. J. Chromatogr. A.

[166] Masiá A., Vásquez K., Campo J., Picó Y. (2015). Assessment of two extraction methods to determine pesticides in soils, sediments and sludges. Application to the Túria River Basin. J. Chromatogr. A.

[167] Ponce-Robles L., Rivas G., Esteban B., Oller I., Malato S., Agüera A. (2017). Determination of pesticides in sewage sludge from an agro-food industry using QuEChERS extraction followed by analysis with liquid chromatography-tandem mass spectrometry. Anal. Bioanal. Chem..

[168] Cerqueira M.B.R., Caldas S.S., Primel E.G. (2014). New sorbent in the dispersive solid phase extraction step of quick, easy, cheap, effective, rugged, and safe for the extraction of organic contaminants in drinking water treatment sludge. J. Chromatogr. A.

[169] Cerqueira M.B.R., Guilherme J.R., Caldas S.S., Martins M.L., Zanella R., Primel E.G. (2014). Evaluation of the QuEChERS method for the extraction of pharmaceuticals and personal care products from drinking-water treatment sludge with determination by UPLC-ESI-MS/MS. Chemosphere.

[170] Rossini D., Ciofi L., Ancillotti C., Checchini L., Bruzzoniti M.C., Rivoira L., Fibbi D., Orlandini S., Del Bubba M. (2016). Innovative combination of QuEChERS extraction with on-line solid-phase extract purification and pre-concentration, followed by liquid chromatography-tandem mass spectrometry for the determination of non-steroidal anti-inflammatory drugs and their metabolites in sewage sludge. Anal. Chim. Acta.

[171] Ramos S., Homem V., Santos L. (2019). Development and optimization of a QuEChERS-GC–MS/MS methodology to analyse ultraviolet-filters and synthetic musks in sewage sludge. Sci. Total Environ..

[172] Rede D., Teixeira I., Delerue-Matos C., Fernandes V.C. (2024). Assessing emerging and priority micropollutants in sewage sludge: Environmental insights and analytical approaches. Environ. Sci. Pollut. Res..

[173] Benedetti B., Majone M., Cavaliere C., Montone C.M., Fatone F., Frison N., Laganà A., Capriotti A.L. (2020). Determination of multi-class emerging contaminants in sludge and recovery materials from waste water treatment plants: Development of a modified QuEChERS method coupled to LC–MS/MS. Microchem. J..

[174] Angeles-de Paz G., Ledezma-Villanueva A., Robledo-Mahón T., Pozo C., Calvo C., Aranda E., Purswani J. (2023). Assembled mixed co-cultures for emerging pollutant removal using native microorganisms from sewage sludge. Chemosphere.

[175] Ajibola A.S., Tisler S., Zwiener C. (2020). Simultaneous determination of multiclass antibiotics in sewage sludge based on QuEChERS extraction and liquid chromatography-tandem mass spectrometry. Anal. Methods.

[176] Montemurro N., Joedicke J., Pérez S. (2021). Development and application of a QuEChERS method with liquid chromatography-quadrupole time of flight-mass spectrometry for the determination of 50 wastewater-borne pollutants in earthworms exposed through treated wastewater. Chemosphere.

[177] Barker S.A., Long A.R., Short C.R. (1989). Isolation of drug residues from tissues by solid phase dispersion. J. Chromatogr. A.

[178] Barker S.A. (2000). Matrix solid-phase dispersion. J. Chromatogr. A.

[179] Triñanes S., Casais M.C., Mejuto M.C., Cela R. (2016). Matrix solid-phase dispersion followed by liquid chromatography tandem mass spectrometry for the determination of selective ciclooxygenase-2 inhibitors in sewage sludge samples. J. Chromatogr. A.

[180] Sánchez-Brunete C., Miguel E., Albero B., Tadeo J.L. (2010). Determination of triclosan and methyl triclosan in environmental solid samples by matrix solid-phase dispersion and gas chromatography-mass spectrometry. J. Sep. Sci..

[181] González-Mariño I., Rodríguez I., Quintana J.B., Cela R. (2010). Matrix solid-phase dispersion followed by gas chromatography-mass spectrometry for the determination of triclosan and methyl triclosan in sludge and sediments. Anal. Bioanal. Chem..

[182] Casado J., Rodríguez I., Carpinteiro I., Ramil M., Cela R. (2013). Gas chromatography quadrupole time-of-flight mass spectrometry determination of benzotriazole ultraviolet stabilizers in sludge samples. J. Chromatogr. A.

[183] Casado J., Castro G., Rodríguez I., Ramil M., Cela R. (2015). Selective extraction of antimycotic drugs from sludge samples using matrix solid-phase dispersion followed by on-line clean-up. Anal. Bioanal. Chem..

[184] Cerqueira M.B.R., Soares K.L., Caldas S.S., Primel E.G. (2018). Sample as solid support in MSPD: A new possibility for determination of pharmaceuticals, personal care and degradation products in sewage sludge. Chemosphere.

[185] Li M., Sun Q., Li Y., Lv M., Lin L., Wu Y., Ashfaq M., Yu C. (2016). Simultaneous analysis of 45 pharmaceuticals and personal care products in sludge by matrix solid-phase dispersion and liquid chromatography tandem mass spectrometry. Anal. Bioanal. Chem..

[186] Montes R., Rodríguez I., Casado J., López-Sabater M.C., Cela R. (2015). Determination of the cardiac drug amiodarone and its N-desethyl metabolite in sludge samples. J. Chromatogr. A.

[187] Celano R., Rodríguez I., Cela R., Rastrelli L., Piccinelli A.L. (2014). Liquid chromatography quadrupole time-of-flight mass spectrometry quantification and screening of organophosphate compounds in sludge. Talanta.

[188] Albero B., Pérez R.A., Sánchez-Brunete C., Tadeo J.L. (2012). Occurrence and analysis of parabens in municipal sewage sludge from wastewater treatment plants in Madrid (Spain). J. Hazard. Mater..

[189] Castro G., Ramil M., Cela R., Rodríguez I. (2021). Identification and determination of emerging pollutants in sewage sludge driven by UPLC-QTOF-MS data mining. Sci. Total Environ..

[190] Arthur C.L., Pawliszyn J. (1990). Solid phase microextraction with thermal desorption using fused silica optical fibers. Anal. Chem..

[191] Zhang Z., Yang M.J., Pawliszyn J. (1994). Solid-Phase Microextraction. A Solvent-Free Alternative for Sample Preparation. Anal. Chem..

[192] Li J., Wang Y.-B., Li K.-Y., Cao Y.-Q., Wu S., Wu L. (2015). Advances in different configurations of solid-phase microextraction and their applications in food and environmental analysis. TrAC Trends Anal. Chem..

[193] Risticevic S., Vuckovic D., Pawliszyn J. (2010). Solid-Phase Microextraction.

[194] Wu S.-F., Ding W.-H. (2010). Fast determination of synthetic polycyclic musks in sewage sludge and sediments by microwave-assisted headspace solid-phase microextraction and gas chromatography-mass spectrometry. J. Chromatogr. A.

[195] Vallecillos L., Pocurull E., Borrull F. (2013). A simple and automated method to determine macrocyclic musk fragrances in sewage sludge samples by headspace solid-phase microextraction and gas chromatography–mass spectrometry. J. Chromatogr. A.

[196] Pedersen-Bjergaard S., Rasmussen K.E. (1999). Liquid-liquid-liquid microextraction for sample preparation of biological fluids prior to capillary electrophoresis. Anal. Chem..

[197] Rezaee M., Yamini Y., Faraji M. (2010). Evolution of dispersive liquid–liquid microextraction method. J. Chromatogr. A.

[198] Baltussen E., Sandra P., David F., Cramers C. (1999). Stir bar sorptive extraction (SBSE), a novel extraction technique for aqueous samples: Theory and principles. J. Microcolumn Sep..

[199] Camino-Sánchez F.J., Rodríguez-Gómez R., Zafra-Gómez A., Santos-Fandila A., Vílchez J.L. (2014). Stir bar sorptive extraction: Recent applications, limitations and future trends. Talanta.

[200] David F., Ochiai N., Sandra P. (2019). Two decades of stir bar sorptive extraction: A retrospective and future outlook. TrAC Trends Anal. Chem..

[201] Ferreira A.M.C., Möder M., Laespada M.E.F. (2011). Stir bar sorptive extraction of parabens, triclosan and methyl triclosan from soil, sediment and sludge with in situ derivatization and determination by gas chromatography–mass spectrometry. J. Chromatogr. A.

[202] Moein M.M., Abdel-Rehim A., Abdel-Rehim M. (2015). Microextraction by packed sorbent (MEPS). TrAC Trends Anal. Chem..

[203] Abdel-Rehim M. (2011). Microextraction by packed sorbent (MEPS): A tutorial. Anal. Chim. Acta.

[204] Maia M.R., Arcanjo A.L.P., Pinho G.P., Silvério F.O., Maia M.R., Arcanjo A.L.P., Pinho G.P., Silvério F.O. (2017). Solid-Liquid Extraction with Low Temperature Purification Coupled with Gas Chromatography and Mass Spectrometry for Determination of Polychlorinated Biphenyls in Sewage Sludge. J. Braz. Chem. Soc..

[205] Pereira N.G.F., Silvério F.O., Pinho G.P. (2020). Optimisation, validation and application of the solid-liquid extraction with low-temperature purification followed by gas chromatography-mass spectrometry for determination of phthalates in sewage sludge. Int. J. Environ. Anal. Chem..

[206] Andrade V.F., Durães A.F.S., Cassimiro D.L., de Pinho G.P., Silvério F.O. (2017). Fast extraction of polychlorinated dibenzo-p-dioxin and polychlorinated dibenzofuran in sewage sludge and soil samples. J. Environ. Sci. Health Part B.

[207] Shoemaker J.A. Determination of Selected Organic Contaminants in Drinking Water by Direct Aqueous Injection—Liquid Chromatography/Tandem Mass Spectrometry (DAI-LC/MS/MS). 2009, p. 40. https://www.epa.gov/sites/default/files/2015-06/documents/epa-538.pdf.

[208] Larsson E., Rabayah A. (2013). Sludge removal of nonsteroidal anti-inflammatory drugs during wastewater treatment studied by direct hollow fiber liquid phase microextraction. J. Environ. Prot..

[209] Grześkowiak T., Czarczyńska-Goślińska B., Zgoła-Grześkowiak A. (2016). Current approaches in sample preparation for trace analysis of selected endocrine-disrupting compounds: Focus on polychlorinated biphenyls, alkylphenols, and parabens. TrAC Trends Anal. Chem..

[210] Keçili R., Büyüktiryaki S., Dolak İ., Hussain C.M., Mustansar Hussain C. (2020). 5—The use of magnetic nanoparticles in sample preparation devices and tools. Handbook of Nanomaterials in Analytical Chemistry.

[211] Luque-Muñoz A., Vílchez J.L., Zafra-Gómez A. (2017). Multiclass method for the determination of pharmaceuticals and personal care products in compost from sewage sludge using ultrasound and salt-assisted liquid–liquid extraction followed by ultrahigh performance liquid chromatography-tandem mass spectrometry analysis. J. Chromatogr. A.

[212] Lara-Gonzalo A., Sánchez-Uría J.E., Segovia-García E., Sanz-Medel A. (2012). Selected ion storage versus tandem MS/MS for organochlorine pesticides determination in drinking waters with SPME and GC-MS. Int. J. Environ. Anal. Chem..

[213] García-Córcoles M.T., Rodríguez-Gómez R., de Alarcón-Gómez B., Çipa M., Martín-Pozo L., Kauffmann J.-M., Zafra-Gómez A. (2019). Chromatographic Methods for the Determination of Emerging Contaminants in Natural Water and Wastewater Samples: A Review. Crit. Rev. Anal. Chem..

[214] Petrovic M., Farré M., de Alda M.L., Perez S., Postigo C., Köck M., Radjenovic J., Gros M., Barcelo D. (2010). Recent trends in the liquid chromatography–mass spectrometry analysis of organic contaminants in environmental samples. J. Chromatogr. A.

[215] Farré M., Kantiani L., Petrovic M., Pérez S., Barceló D. (2012). Achievements and future trends in the analysis of emerging organic contaminants in environmental samples by mass spectrometry and bioanalytical techniques. J. Chromatogr. A.

[216] Castro G., Roca M., Rodríguez I., Ramil M., Cela R. (2016). Identification and determination of chlorinated azoles in sludge using liquid chromatography quadrupole time-of-flight and triple quadrupole mass spectrometry platforms. J. Chromatogr. A.

[217] Wood R. (1999). How to validate analytical methods. TrAC Trends Anal. Chem..

[218] Trufelli H., Palma P., Famiglini G., Cappiello A. (2011). An overview of matrix effects in liquid chromatography–mass spectrometry. Mass Spectrom. Rev..

[219] Parr M.K., Schmidt A.H. (2018). Life cycle management of analytical methods. J. Pharm. Biomed. Anal..

[220] Schwesig D., Borchers U., Chancerelle L., Dulio V., Eriksson U., Farré M., Goksoyr A., Lamoree M., Leonards P., Wegener J.-W. (2011). A harmonized European framework for method validation to support research on emerging pollutants. TrAC Trends Anal. Chem..

[221] Cortese M., Gigliobianco M.R., Magnoni F., Censi R., Di Martino P. (2020). Compensate for or Minimize Matrix Effects? Strategies for Overcoming Matrix Effects in Liquid Chromatography-Mass Spectrometry Technique: A Tutorial Review. Molecules.

[222] Matuszewski B.K., Constanzer M.L., Chavez-Eng C.M. (2003). Strategies for the Assessment of Matrix Effect in Quantitative Bioanalytical Methods Based on HPLC−MS/MS. Anal. Chem..

[223] (2003). Method 8000C, Revision3 EPA. https://archive.epa.gov/epawaste/hazard/testmethods/web/pdf/method%208000c%2c%20revision%203%20-%202003.pdf.

[224] (2019). Capability of Detection—Part 6: Methodology for the Determination of the Critical Value and the Minimum Detectable Value in Poisson Distributed Measurements by Normal Approximations.

