# Supplementary material for: Extraction Methods of Emerging Pollutants in Sewage Sludge: A Comprehensive Review
Source: Toxics. 2025 Aug 5;13(8):661. doi: 10.3390/toxics13080661 (PMC12390028; doi:10.3390/toxics13080661)
Supplement: Supplementary file 1 [file toxics-13-00661-s001.zip › toxics-3757585-supplementary.pdf]

Supporting Information

Table S1. Classification of emerging pollutants

Category	Group of compounds
Flame retardants	Brominated flame retardants (BFRs)
	Per- and polyfluoroalkyl substances (PFAs)
	Alkylphenols and nonylphenols (APs)
	Organophosphate flame retardants (OPFRs)
Plasticizers	Butiyltin compounds
	Phthalates
	Bisphenol A and analogues
Other industrial compounds	Anticorrosives (tolytrizole, Tolytriazole, tris buthoxyethyl phosphate and tris(2-chloroethyl) phosphate)
	Surfactants
	Aliphatic primaries amines/nitrosamines
Recreational consumption/Food	Artificial sweeteners
	Nervous stimulants (illicit drugs and caffeine)
Pharmaceutical Active Compounds (PhACs)	Hormones
	Non-steroidal anti-inflammatory drugs/analgesics
	Antibiotics
	Anti-epileptics
	Anti-depressants, sedative hypnotics, anxiolytic
	β-blockers
	Lipid regulators
	Blood pressure medicines
	Bronchodilators
	Anticancer drugs
	Others ((histamine antagonist, anti-allergenic, anesthetic, anti-diabetic, antiemetic, ant parasitic, antiarrhythmic/cardiac drugs, sympathomimetic, sexual function agents)
Personal care products (PCPs)	Antimicrobial agents
	Musks
	UV-filters
	Azoles (antimycotic, preservatives)
	Parabens
	Insect repellents
	Retinoids
	UV-filters
	Azoles
	Parabens
	Insect repellents
	Retinoids

Table S2. Details of the procedure of mechanical shaking extraction of EPs in sewage sludge**.**

Analytes	SS mass (g)	Pre-treatment	Solvent volume (mL)	Extraction details	Ref.
Flame retardants (6 PBDEs)	0.5	Dried sewage sludge	10	mechanical shaking for 2 h, 20 °C, 300 rpm, 2 consecutive cycles with each solvent; organic phase collection (with 25% KOH in MeOH), centrifugation (4200 x g, 5 min)	[1]
PCPs (5 benzophenones -type UV and 2 benzotriazoles)	0.05-01	Freeze-dried		2 cycles of shaking 30 min + centrifugation (4000 rpm, 4 min) and concentrated by rotary evaporation	[2]
Plasticisers (2 butynyl compounds)	0.2	Dried at 40 °C sieved at 0.0108 mm and sterilise by γ-irradiation	20	Mechanically shaken on the elliptical table shaker for 15 h in the dark, centrifuge for 15 min at 4200 rpm	[3][a]
Plasticisers (6 phthalates)	5	Dried at 4 °C in dark conditions before being ground and sieved	60	shaken for 12 h at 180 rpm in a mechanical shaker. Organic phase was collected and concentrated in a rotary evaporator	[4]
Plasticisers (4 phthalates)	5	Dried at 4 °C in dark conditions before being ground and sieved		shaken for 12 h at 180 rpm in a mechanical shaker. Organic phase was collected and concentrated in a rotary evaporator	[5]
Plasticisers (DEHP)	1	Freeze-dried and crushed	80	mechanical shaking for 12 h	[6]
Plasticisers (4 phthalates)	1	Air-dried and crushed	80	extracted for 12 h at 250 rpm	[7]

Plasticisers (BPA and analogues)	Non-specified	5	Vortex for 10 s and centrifuge (6000 rpm, 15 min). Cycles: 3	[8]

PBDEs: Polybrominated diphenyls ethers, PCPs: personal care products, EDCs: endocrine disrupting chemicals, DEHP: Bis(2-ethylhexyl) phthalate, BPA: bisphenol-A, Ref: references.
[a]Different extraction methods were compared and there were no significant differences between them.

Table S3. Details of the procedure of Soxhlet extraction of EPs in sewage sludge.

Analytes	SS mass (g)	Pre-treatment	Solvent volume (mL)	Extraction details	Ref.
Flame retardants (4 PBDEs)					[9]
Flame retardants (13 PBDEs)	1	Freeze-dried	6	Addition 1.2 anhydrous sodium sulphate ground and sieve + solvent, vortex 10 min, centrifuged 3 min at 2500 rpm, addition of sulfuric acid 30 min of separation. ELISA analysis	[10]
PCPs (6 musks)	Soxhlet	Dried	-	Soxhlet-extraction for 72 h	[11]
PCPs (4 polycyclic musks)	1	Freeze-dried and sieved by 30-mesh		Extracted for 72 h, addition of activated Cu (10% HCl), concentrated in a rotary evaporator, eluted with n-hexane, and the solvent was replaced with n-hexane, transferred to Neutral silica gel and alumina (2:1)	[12]

PBDEs: Polybrominated diphenyls ethers, PCPs: personal care products, EDCs: endocrine disrupting chemicals, Ref: references.

Table S4. Details of the procedure of UAE of EPs in sewage sludge**.**

Analytes	SS mass (g)	Pre-treatment	Solvent volume (mL)	Extraction details	Ref.
EDCs (3) NSAIDs (4)		Non-specified		Previously described [13]	[14]

EPs (119)		Non-specified	2	Ultrasonicated for 15 min and directly filtered by 0.45 µm regenerated cellulose	[15]
EPs (43)	2	Freeze-dried for 48 h at –60 °C, ground int uniform particles and sieve by 2 mm mesh	5+5	Sonicated for 10 min and centrifuge at 8500 rpm for 10 min. Repeated 2-3 times.	[16]
EPs (178)		Non-specified		Ultrasonicated and concentrated in a TurboVap evaporator	[17]
EPs (68)	0.1	Freeze-dried	2+2+2	Suspension was vortexed, ultra-sonicated for 15 min at 50 °C, and centrifuged (2260 g, 15 min). Repeated 2 more times different solvent mixtures.	[18]
EPs (41 illicit drugs)	1	Freeze-dried, sequentially sieved by 2 mm, 500 µm and 125 µm, stored in dark at –20 °C	10	Shaken 15 s, sonicated for 10 min and centrifuged at 1200 rcf for 15 min. The supernatant was, diluted with distilled water, adjusted to pH 6 with 1 M NaOH and bought to final volume with distilled water	[19]
Flame retardants (HBCD and TBBPA)	0.5	Freeze-dried, grounded and stored at –20 °C	10	Sonication (60 min at 30 °C), agitation (3 h, 25 °C), centrifugation	[20]
Flame Retardants (13 PFAS)	1	Dried at 40°C until constant weight	10	Ultrasonicated at 40 °C for 30 min and centrifuge at 3000 rpm for 15 min	[21]

Flame retardants (OPFRs, PBDEs and NBFRs)	0.1	Freeze-dried, homogenised and sieve by 120 µm	3x10	Vortex for 1 min, ultrasonicated for 10 min, centrifuged at 3000 rpm for 10 min, 2 cycles	[22]
Flame retardants, plasticiser and PCPs (23 compounds)	0.5	Freeze-dried, Sieved < 100 µm, store at – 18 °C until analysis	3x3	Sonicated in an ultrasonic bath for 7 min, 3 cycles and centrifugation (2900 x g, 10 min)	[23]
Flame retardants (PBDEs and HBCD)	2-5	Freeze-dried, ground and sieved by 2 mm. stored in amber-glass bottles	30	Ultrasonicated (30 min), centrifuged (1800 rpm, 5 min), cycles: 2, concentrated in a rotavapor. Copper addition	[24][2]
Flame retardants (7 BDEs)	0.5	Freeze-dried, ground and stored on darkness at 4 °C	8	Pulsation of 0.5 and 65% of power at 0 °C in an ice-water bath for 20 s, 2 cycles). Centrifugation (5 min, 3000 rpm)	[25]
Flame retardants (7 PCBs)	0.5	Freeze-dried	10+10	Tubes were placed into a rotary shaker for 30 min at 150 rpm and ultrasonicated for 30 min at 35 °C, centrifuged (10 min at ~1328 × g, 20 °C) .	[26]
Industrial/Domestic products (11 PFASs)	0.3	Freeze-dried, homogenised, sieved until 100-mesh stored at – 20 °C until analysis	5	Ultrasonic bath at 50 °C, 20 min, centrifuge at 4500 rpm for 5 min, 3 cycles	[27]

Industrial surfactants and flame retardants (APEs and BDEs)	5	Centrifuged and supernatant discarded	30	Pre-treated with 20 g of anhydrous sodium sulphate, ultrasonicated at 55 °C, 45 min, cycles: 2	[28]
Industrial/Domestic products (PFAS)	0.5	Freeze-dried	5+1	Sonication (15 min), centrifugation (3000 rpm, 10 min)	[29]
Industrial al domestic products (46 PFAS)	1	Freeze-dried, ground to homogeneity, sieved through a 150 µm mesh, stored at –20 °C	4	Each sample was treated with solvent and 0.162 g of 60 mM $K_2S_2O_8$ solution and 0.15 mL of 150 mM NaOH, sonication for 5 min. Then, the samples were placed in a thermostatic water bath at 85 ◦C for 6 h. After the reaction, the samples were cooled to RT in an ice water bath, and the pH was adjusted to 5–9 by adding HCl, 3 cycles.	[30]
PCPs (6 azoles)	0.1	Freeze-dried, homogenised	4	Vortex for 2 min, ultrasonicate for 15 min, centrifuge (4000 rpm, 5 min, 4 °C), 2 cycles	[31]
PCPs (musks)	1	Freeze-dried	20+20	Rotation-shaker for 150 min and ultrasonic batch for 3 min, 2nd cycle with n-hexane (5 mL). Centrifugation (3000 rpm)	[32]
PCPs and steroids (14)	0.2-1	Homogenised, Freeze-dried and stored at –20 °C	6	30 min at 30 °C	[33]
PCPs (19 biocides)	2	Freeze-dried	10	2 cycles of vortex 30 s, ultrasonicated 15 min, centrifuged 2800 rpm 10 min.	[34]
PCPs (TCB and TCC)	0.25	Homogenise the sample in the container with a clean spatula	20	Sonicated 30 min, centrifugation (3000 rpm, 5 min)	[35][1]

PCPs and EDCs (6 retinoids 7 EDCs)	0.1	Freeze-dried and kept it at – 18 °C until extraction	5	Ultrasonicated (10 min), centrifugated (2375 x g, 5 min), 2 cycles	[36]
PCPs and EDC (5 retinoids and 7 EDCs 7)	0.06-0.1	Freeze-dried		Methods described by Zhou et al. (2019) [36]	[37]
PCPs (TCC and transformation products)	1.5	Extracted in fresh weight	15/20/15+20	Vortex after phosphate buffer addition, sonicate 30 min after ACN addition, centrifuge and filtration	[38]
PCPs (5 musks)	0.2	Freeze-dried and kept at RT in amber glass bottles until analysis	20	Ultrasonicated in water bath at 40 Hz intensity and 30 °C for 30 min, centrifuge (3000 rpm, 5 min)	[39]
PCPs (TCB)	0.1	Freeze-dried	10	Vortex with the extraction solvent (1 min), ultrasonicated (120 Hz, 30 °C, 20 min, 2 cycles), centrifugation (3500 rpm, 5 min)	[40]
PCPs (TCS and metabolites)	1-1.5	Frozen		Vortex, placed in an ultrasonic bath, centrifuged	[41]
PCPs (musks)	0.5	Freeze-dried	10+10	Rotation-shaker (30 min, 35 °C, 150 rpm), centrifugation (1328 x g, 10 min)	[42]
PhACs (16)	1 and 1.5	Freeze-dried, sieved<100 µm and stored at - 30 °C	9	Shaken for 30 s, ultrasonicated 15 min and centrifugation.	[43]
PhACs (18 estrogens)	0.5	Freeze-dried	25	Ultrasonicated and centrifuged	[44]

PhACs and EDCs (9)	0.04	Homogenised using a mortar and pestle, dried at 60 °C for 48 h and stored at −18 °C	8	Sonication for 30 min at 50 °C + centrifugation	[13]
PhACs (15 antidepressants)	0.2	Freeze-dried	8	Shaken vigorously for 15 min in a rotary extractor. Addition of 4 mL of Milli-Q water. Sonicated in bath for 15 min, centrifugation (320 x g, 5 min)	[45]
PhACs (4 antibiotic and 2 estrogens)	2	Freeze-dried and grinded to 425 μm		Vortex for 1 min, sonicated for 20 min, centrifuged at 4800 rpm for 10 min, filtered by 0.45 μm	[46]
PhACs (22 antibiotics)	0.5	Freeze-dried	10	Vortex for 1 min, ultrasonicated for 15 min, centrifuged at 13710 x g for 10 min	[47]
PhACs (13 quinolones antibiotics)	0.5	Centrifuged and dried at 60 °C until constant weight, ground and store in dark at 4 °C until analysis	5	2 cycles: vortex 2 min, sonicated 15 min, centrifugation 20 min at 3634 x g	[48]
PhACs (NSAIDs, lipid regulators and antibiotics)	0.2	Freeze-dried sieved by 0.2 mm stored at −20 °C until analysis	6	Sonicated for 15 min; centrifugated at 4000 rpm for 10 min; cycles: 3	[49]
PhACs (21 progestogens)	0.5	Addition of sodium azide,	10	Vortex for 30 s; placed in ultrasonicated bath for 15 min; centrifuged at 1371 x g for 10 min; cycles: 3	[44]

		samples were freeze-dried, homogenized, and stored at 4 °C until use			
PhACs (26)	0.5	Freeze-dried, ground with a mortar; sieved ≤0.5 mm; and stored at -20 °C until analysis	10	Vortex 30 s; ultrasonicated for 10 min; centrifugate at 5000 rpm for 10 min; cycles: 3	[50]
PhACs (13)	0.1	Frozen at –24 °C immediately after collection and freeze-dried	2	Vortex, sonicated 15 min at 40 kHz, centrifuged (12000 rpm, 10 min) 2 cycles	[51]
PhACs (8)	1	Stored at –20 °C left overnight at 4 °C for defrosting 2 days before extraction	6+15	Sonicated 10 min, vortex 10 s, sonicated 10 min, centrifuge (10900 x g, 10 min, 20 °C)	[52]
PhACs (11 acidic drugs and estrogenic hormones)	0.5	Freeze-dried	15+20	Sonicated 20 min, centrifugation (10000 rpm, 5 min)	[53]
PhACs (5)	0.1	Dehydrated by centrifugation. Freeze-dried	9	Shaken for 30 s, ultrasonicated 15 min and centrifugation.	[54]

PhACs (5 NSAIDs)	-	Stored in bottles made of HDPE plastic at –20 °C prior to analysis	20	Solvent was added and hand shake thoroughly, sonicated (45 min) centrifuged (15 min, 3000 rad/min) 1 cycle with each solvent	[55]
PhACs (4)	1-1.5	Frozen		Vortex, placed in an ultrasonic bath, centrifuged and filtrated	[56]
PhACs (5)	1-2	Freeze-dried/dehydrated		Described by [54]	[57]
PhACs (69)	0.1	Freeze-dried/milled	12	Sonication for 15 min at 50 °C, centrifuged at 4000 g 10 min, 2 cycles	[58]
PhACs and illicit drugs (148)	0.1	Freeze-dried	2	Vortex for 1 min, ultrasonicated for 15 min at 50 °C, centrifuge0d at 4000 rpm for 10 min, 3 cycles	[59]
PhACs (7 antibiotics)	1	Freeze-dried	3	Vortex-mixed and sonicated for 15 min, 3 cycles, centrifuged 10 min 2900 xg.	[60]
Plasticisers/PCPs (3 alkylphenols)	0.1	Freeze-dried	20+10	Ultrasonicated 3 times: 2x H-DCM + 1x DCM-acetone	[61]
Plasticisers (2 butyltin compounds)	0.2	Dried at 40 °C sieved at 0.0108 mm and sterilise by γ-irradiation	4	Ultrasonicated for 30 min and centrifuge for 15 min at 4200 rpm	[3][1]
Plasticisers (7)	0.2	Freeze-dried, ground and sieved ≤40 mesh, stored at 4 °C	5	Ultrasonicated for 15 min; centrifugate at 9000 x g for 5 min	[62]

Plasticisers/PCPs (4 nonylphenols ethoxylates)	0.5	Freeze-dried, grinded with a mortar and pestle; sieved until 1 mm and homogenised and stored at – 18 °C until analysis	10	Ultrasonicated for 5 min, centrifuged for 5 min, Filter by anhydrous sodium sulphate column.	[63]
Plasticisers (9)	0.2	Freeze-dried	5	Ultrasonicated for 5 min, shaking, 30 min, 2 cycles), centrifugation (4500 g, 5 min)	[64]
PPCPs (17 azoles)	1	Freeze-dried-mill-sieved <0.5 mm, stored at – 20 °C	5	290 w, 50/60 Hz - 15 min	[65]
PPCPs and industrial products (10)	0.1	Freeze-dried, ground and sieved ≤80 mesh	8	40 kHz, 300 W for 15 min and centrifuge 3000 rpm for 5 min at 4 °C	[66]
PPCPs (5 azoles)	-	Freeze-dried		Described by Huang et al. 2010	[67]
PPCPs (7 antibiotics and antibacterial agents)	0.2	Freeze-dried and homogenised	8	Vortex for 1 min, ultrasonicated for 10 min, centrifugated at 4000 rpm for 5 min, 2 cycles	[68]
PPCPs (14)	0.1	Air-dried at room temperature sieved to 0.5	8	Vortex for 2 min, ultrasonicated for 20 min, centrifuged at 3000 rpm for 10 min, 3 cycles	[69]

		mm store at 0 °C until analysis.			
PPCPs (10)	-	Freeze-dried sieve by 80 µm and stored at –20 °C	6+2+4	Vortex (1 min), ultrasonicated (20 min at 30 °C), centrifuged (4000 g, 10 min), cycles: 5	[70]
PPCPs (22)	0.8	Freeze-dried	24	Vortex-stirred, ultrasonicated (RT, 30 min, 50 W, 60 Hz cycles:2), centrifuged (5 min, 2655 x g), supernatant transferred to DI-SPME	[71]
PPCPs and metabolites (19)	2	Freeze-dried, homogenised, crushed and store at –18 °C prior to analysis	15	Sonication for 5 min, cycles: 3. Centrifugation (2900 xg, 20 min)	[72][b]

HBCD: hexabromocyclododecane, TBBPA: tetrabromobisphenol A, PCPs: personal care products, PhACs: pharmaceutical compounds, PFAS: Per- and Polyfluoroalkyl substance, EDCs: endocrine disrupting compounds, PPCPs: pharmaceutical and personal care products, APEs: alkylphenols ethoxylates, BDEs: brominated diphenyl ethers, OPFRs: organophosphate flame retardants, PBDEs: Polybrominated diphenyls ethers, NBFRs: novel brominated flame retardants, NSAIDs: nonsteroidal ant-inflammatory drugs, TCS: triclosan, TCC: triclocarban. Ref: references.

[1]*According to methods described by EPA* [73] [2]*According to methods described by EPA* [74].

[a]*Different extraction methods were compared and there were no significant differences between them.*

[b]*Different extraction methods were compared and validated.*

Table S5. Details of the procedure of MAE of EPs in sewage sludge**.**

**Analytes**	**SS mass (g)**	**Pre-treatment**	**Solvent volume (mL)**	**Extraction details**	**Ref.**
EDCs (12)	1	Stirred to homogenise and	5	Power:300 W, 10 min + cooling down microwave fan (10 min) + cool down RT (10 min) + filtered	[75]

		air-dried for 12 h at RT			
EDCs (2 butynyl compounds)	0.2	Dried at 40 °C sieved at 0.0108 mm and sterilise by γ-irradiation	4	Power: 1200 W for 4 min	[3][a]
EDCs (13)	0.5-2	Freeze-dried and sieved <2 mm	10	Power: 350 W, 3 min, centrifugation (5000 rpm, 10 min)	[76]
EDCs (hormones and corticoids)	0.1	Freeze-dried	10	Power: 500 W, 4 min, MeOH at 65 °C, cooling down 5 min, RT for 10 min, filtration by 0.20-µm PET syringe	[77]
EDCs (14 phenols)	0.25	Dewatered by centrifugation and kept at 4 °C	30.5	Power max: 600 W, T max: 80 °C, ramp: 15 min, hold: 30 min, cooling: 20 min, filtration: Celite.	[78]
EDCs (BPA)	0.2	Deactivated with sodium azide sol. (0.2% w/w), shaking for 2 h, 200 rpm, centrifuged (30 min, 10000 rpm) and dried at 60 °C (12 h). addition of 5 g of anhydrous sodium sulphate	20	3 cycles, power: 1200 W ramp to 110 °C for 10 min and held at 110 °C for 10 min	[79] [1]
EPs (61)	0.2	Frozen and stored at –20 °C, freeze-dried,	14	For ilicit drugs: 1) 5 min, 120 W at 90 °C. 2) 5 min, 100 W at 90 °C. 3) Cooling phase: 5 min // For PhACs: 1) 5 min, 450 W, 70 °C. 2) 5 min, 400 W, 70 °C. 3) Cooling phase: 10 min.	[80]

		ground and sifted.			
PCPs (4 LAS)	5	Dried at 105 °C milled at 0.25 µm+ store at 4 ° C	25	25 min (20 min for holding) at 105 °C	[81]
PhACs (4 NSAIDs)	0.5	Freeze-dried, grinding mill and store at 5 °C		Power: 700 W - 5 min up to 60 °C and 600 W - 5min up to 100 °C	[82]
PhACs (5 fluoroquinolone antibiotics)	2	Dried	15	Power: 500 W, 15 min for extraction, cool down 10 min with microwave fan + 10 min at RT	[83]
PhACs (13 quinolones antibiotics)	0.5	Centrifuged and dried at 60 °C until constant weight, ground and store in dark at 4 °C until analysis	10	5 min for holding at 87 °C, power: 1000 W, air-flow cooled until less 45 °C, centrifuge for 30 min at 3634 x g	[48][a]
PhACs and Ilicit drugs (18)	Among 1-3	Frozen and freeze-dried	20	Heated to 120 °C for 30 min	[84]
PhACs and stimulants (5 antidepressants and caffeine)	1-5	Freeze-dried and sieved in a 32-mesh (0.5 mm) sieve and then stored in	20	Vortex (30 s), warm it up in the microwave 20 s and power: 10 W. Cool at RT, 3 cycles. Centrifuged 2 min at 2000 rpm	[85,86]

		dark in glass bottles at RT			
Plasticisers (phthalates)	10	Freeze-dried, ground and sieved	100	Addition of 50 µL of decafluorobiphenyl solution (40 µg/mL). Mixed with solvent for 16–18 h of microwave extraction using a Soxhlet extractor. Addition of 5 g of an activated copper strip fixed to 1 mL with acetonitrile for GC–MS analysis. The GC–MS instrument (QP2010Ultra equipped with DB-5MS column,	[87]
PPCPs (22)	1	Freeze-dried, homogenised, sieved and stored at –20 °C until analysis.	10	Power: 500 W for 6 min and filter (0.20 µm)	[88]
PPCPs and illicit drugs (90)	0.5	Freeze-dried	25	T: 110 °C, 30 min, filtered and diluted 5% with the extraction solvent	[89]

PhACs: pharmaceutical compounds, NSAIDs: nonsteroidal ant-inflammatory drugs, PCPs: personal care products, EDCs: endocrine disrupting compounds, PPCPs: pharmaceutical and personal care products, BPA: bisphenol, Ref: references.

[1]*According to method described by EPA* [90]

[a]*Different extraction methods were compared and there were no significant differences between them.*

Table S6. Details of the procedure of PLE of EPs in sewage sludge**.**

**Analytes**	**Sample mass (g)**	**Pre-treatment**	**Solvent volume (mL)**	**Extraction details**	**Ref.**
EDCs (estrogens and BPA)	0.5	Freeze-dried, crushed		Pre-treated with 5 g of Florisil, T:80 °C, P: 1500 psi, static extraction time: 8 min, cycles: 2.	[34]
EDCs and caffeine (22)	1	Freeze-dried ground, homogenised and sored at –	-	heating up to 50 °C, P:1500 psi, heat up time: 5 min, static time: 5 min, cycles: 3	[91]

		20 °C prior to use			
EPs (Pharmaceuticals, PFOA, PFOS, 44)	0.1	Freeze-dried		Previously mixed with 0.25 g of Extrelut. T:100 °C, P: 1500 psi, preheating period: 3 min, heat up time: 5 min heating, stati time: 5 min static, flush volume: 80%, purge: 2 min cycles: 3	[92]
PhACs (8 sedative hypnotics)	1	Freeze-dried and sieved by 125 µm stored at RT prior to use		(2 g of diatomaceous earth) T: 100 °C, P: 1500 psi, preheating period: 5 min, static extraction time: 5 min, cycle: 1, flush volume: 60%, nitrogen purge 120 s, final volume: ~15 mL	[93]
Flame retardants (15)	5	Freeze-dried		T: 100 °C, P: 1500 psi, heating time: 5 min, cycles: 3, flush: 80% of the extraction cell, final volume ~150 mL	[94]
Flame retardants (DBDPE)	0.5	Dried at 40 °C, grounded and stored at –18 °C (mixed with 0.5 g copper powder)		T: 100 °C, 10.3 Mpa, flush volume: 90%, cycles: 3	[95]
Flame retardants (7 BDEs)	-	Freeze-dried, ground and stored at 4 °C	-	Florisil+$NaSO_4$+ at 40 °C for 2 min + MEPS	[96]
Flame retardants and PCPs (99 PCBs, musk, etc.)	1	Freeze-dried, homogenised with a mortar and pestle with pre-cleaned sand or pre-baked	50	T: 120 °C, static extraction time: 5 min, cycles: 3, flush volume 100%, nitrogen purge: 60 s	[97]

		sodium sulphate (3:2, w/w)			
Flame retardants (9)	0.2	Freeze-dried, finely ground		T: 130 °C, P: 1.700 psi. Addition of 50 ng TnBP-d27 and activated copper powder.	[98]
Industrial/Domestic products (PFPAs/PFOS)	1	Freeze-dried + previous clean-up		T: 100 °C, cycles: 3 cycles, reconstituted with 10 mL water	[99]
Industrial/domestic products (18 PFCs)	0.5	Freeze-dried		T: 70 °C, 100, cycles: 2, reconstituted in 50 ml of water	[100]
Illicit drugs (20)	0.5	Freeze-dried, ground with a mortar and strored at – 20 °C until analysis		T:50 °C; P: 1250 psi; preheating period: 5 min; heating time: 5 min and static extraction: 5 min, cycles: 1	[101]
PCPs (10 musks)	1	Freeze-dried and sieved by 125 μm	4	(1 g Florisil and 1 g diatomaceous earth and to cover the sample in the cell) T: 80 °C, pre-heating 120 s, cycles: 2, P: 120 s, P 1500 psi; static extraction: 5 min.	[102]
PCPs (TCS and transformation products)	0.5	Dewatered aluminium foil and stored at – 18 °C and Freeze-dried		Preheat: 1 min, heat: 5 min, static: 5 min, cycles: 2, P: 1200 psi, T: 100 °C	[103]
PhACs (10 β-blockers)	1	Freeze-dried and stored at RT	-	Equilibration: 5 min, static time: 5 min, flush volume: 60%, purge time: 60 s, T: 50 °C, cycles: 2, final volume ~22 mL	[104]
PhACs (5 estrogens)	0.5	Freeze-dried		Preheat: 5 min, heat: 5 min, static solvent extraction: 5 min, flush: 100% of the volume, purge: 2 min, cycles: 3. T: 100 °C, P: 100 bar	[105]

PhACs (carbamazepine)	0.5	Freeze-dried and homogenised	20	T: 140 °C, P: 1500 psi, pre-heating period: 6 min, static extraction: 5 min, flush: 20 mL, centrifugation at 7650 x g for 15 min.	[106][a]
PhACs (14)	0.5	Freeze-dried and sieved by 0.45 mm		2 + 1 g of diatomite; extraction: 3 cycles (300 s x3) of 100 °C, 10 Mpa, preheating period: 60 s, flush volume: 40%, nitrogen purge: 60 s, final volume ~50 mL	[107]
PhACs (22 sulfonamide antibiotics)	2	Freeze-dried - 50 °C, 0.044 BAR vacuum and stored at -30 °C until analysis		T: 50 °C, P: 1500 psi; preheating period: 5 min; static extraction: 5 min; flush volume: 100%, nitrogen purge: 60 s, cycles: 3	[108]
PhACs (13 quinolones antibiotics)	0.5	Centrifuged and dried at 60 °C until constant weight, ground and store in dark at 4 °C until analysis	5	T: 86 °C, P: 1000 psi, preheating time: 1 min, static extraction time: 5 min, solvent flush: 30% of the cell volume, nitrogen purge: 90 s, final volume: ~15 mL	[48][b]
PhACs (9 glucocoinoids)	1	Freeze-dried and sieved by 125 µm		1 g of diatomaceous + (first 2 cycles with *n*-hexane were discard at 40 °C; 1500 psi; preheating period: 5 min; static extraction: 1 min). T: 40 °C; P: 1500 psi; preheating period: 5 min; static extraction: 10 min, cycle: 1	[109]
PhACs (2 anticancer drugs)	0.35	Freeze-dried-mill-sieved <0.5 mm, stored at –	15	T: 100 °C, P: 85 bar, no preheat period; static extraction time: 9 min, cycles: 4	[110]

		20 °C prior to use			
Domestic products (8 sweeteners)	1	Freeze-dried, grinded by mortar and pestle, sieved to 125 and stored at RT		2 gC_{18} + 1 diatomaceous earth, preheating period: 5 min, extraction T: 40 °C, P: 1500 psi, static period: 5 min, cycle: 1, flush volume: 40%, nitrogen purge time: 90 s, extract: ~15 mL	[111]
PhACs (5 antibiotics)	0.5	Freeze-dried for 48 h ground in a mortar and sieved to 0.25 mm	-	completed with diatomaceous earth, T: 80 °C, P: 120 bar, static time: 10 min, cycles: 2	[112]
PhACs (12)	1	Freeze-dried		Preheating: 5 min, heating: 5 min, static period: 15 min, T: 100 °C, P: 100 bar, solvent flush: 10%, nitrogen purge: 150 s, recovery: 11 mL	[113]
PhACs (diclophenac)	0.5	Freeze-dried, ground by a mortar and pestle and store at 4 °C until analysis	20	T: 100 °C, P: 1500 psi, heating time:10 min, static extraction: 5 min, cycles: 1, final volume ~150 mL	[114]
PPCPs and illicit drugs (14)	1-10	Freeze-dried		T: 70 °C, static time: 10 min, purge time: 1 min, cycles: 2	[115]
PPCPs and flame retardants (71)	0.1-2.5 g	Non-specified		Extracted during 10 min, T: 80 °C, P: 120 bars. depend on the compound, condition may change	[116][c]
PPCPs and metabolites (19)	2	Freeze-dried, homogenised,	15	T: 50 °C, pre-heating: 5 min, P: 1500 psi, static extraction time: 5 min, cycles: 2, nitrogen purge: 2 min	[72][d]

		crushed and store at –18 °C prior to analysis			
PPCPs (17)	0.5	Dried (non-specified)		Mixed with Hydromatrix (Thermo Scientific). T: 90 °C, pre-heating: 5 min, P: 1500 psi, static extaction: 5 min, cycles: 3, solvent flush: 100%, nitrogen purge: 60 s	[117]

PhACs: pharmaceutical compounds, PFAS: per and polyfluoroalkyl substances, PFOs: perfluorooctanesulfonic acids, PFCs: perfluorinated compounds, PPCPs: pharmaceutical and personal care products, EDCs: endocrine disrupting compounds, BPA: bisphenol-A, PCBs: polychlorinated biphenyls, MeOH: methanol, ACN: acetonitrile, P: pressure, T: temperature, c: number of cycles, Ref: references.

[a]More extraction techniques were used, and this showed the best efficiency.

[b]Different extraction methods were compared and there were no significant differences between them.

[c]In this study many different compounds were determined and according to the compounds different conditions were applied in each step.

[d]Different extraction method were compared and validated.

Table S7. Details of the procedure of PHWE of EPs in sewage sludge**.**

**Analytes**	**Sample mass (g)**	**Pre-treatment**	**Solvent volume (mL)**	**Extraction details**	**Ref.**
Industrial application (aliphatic primary amines)	1	Freeze-dried and sieved 125μm	-	T: 100 °C, 1500 psi, 2 cycles	[118]
Industrial application (nitrosamines)	5	Freeze-dried and sieved by 125 μm	-	125 °C, 1500 psi, preheating period: 6 min, static extraction: 5 min, 2 cycles	[119]
PhACs (NSAIDs)	0.5	Dried at 40 °C, ground by a	-	T: 120 °C, P: 100 bar, static time 5 min, 5 cycles	[120]

		mortar, sieved <0.5 mm			
PhACs (10 azoles)	1	Freeze-dried	-	Addition of 1 g +1 g of diatomaceous earth, 1 cycle at: 80 °C, 1500 psi, preheating time: 5 min, extraction time: 5 min, flush volume: 60%, purge time: 120 s. Final volume: ~20 mL	[121]
PhACs (23)	0.2	Freeze-dried	-	Heat to 150 °C, mixed with 8 g of silicon carbide	[122]

PhACs: pharmaceutical compounds, HF-LPME: hollow fibre liquid phase microextraction, *HS-SPME:* headspace solid-phase microextraction.

Table S8. Details of the procedure of QuEChERS of EPs in sewage sludge**.**

**Analytes**	**Sample mass (g)**	**Pre-treatment**	**Solvent volume (mL)**	**Extraction details**	**Ref.**
EPs (42)	5	Wet weight	10	Addition of solvent and vortex 1 min. Addition of 4 g of $MgSO_4$, 1 g of NaCl, 1 g of trisodium citrate dihydrate, and 0.5 g of disodium hydrogen citrate sesquihydrate) and vortex 1 min. Centrifuged at 2000 rpm for 15 min.	[123]
PCPs (13 azoles and benzene sulfonamide derivates)	1	Freeze-dried, crushed, sieved by 125 µm	10+10	Addition of cooled water + mix (1 min), addition of ACN + salt packet extraction vortex for 1 min), centrifugation (4000 rpm, 5 min)	[121]
PCPs (19 musks and UV filters)	0.5	Freeze-dried	10	Vortex (2.5 min), ultrasonicated (15 min, 420 W) centrifuged (2670 x g, 15 min)	[124]
PhACs (136)	2	Freeze-dried and homogenized	~23	Solvent addition, shaking $MgSO_4$ addition and centrifugation (10000 x g, 5 min). Supernatant + DMSO + H_2O	[125]
PhACs (13 NSAIDs)	1	Freeze-dried and stored at −20 °C	15	Hand-shaken 15 s, vortex 1 min, addition of 10 mL of CH_3CN; same mix process with addition of 2 g of NaCl and 2 g of $MgSO_4$, repeat mix process and centrifugation (1200 x g, 4 min), filter	[126]

		until analysis			
PhACs (12)	0.1	Dried and store at 4 °C prior to analysis	10	Vigorously shaken for 2 min, 4 g $MgSO_4$ and 1 g NaCl and shaken for 1 min, centrifuged (3200 x g for 5 min)	[127]
PhACs (33)	1	Freeze-dried, homogenized, ground and sieved to < 250 μm	10	Vigorously shaken for 1 min by a vortex mixer (3000 rpm) and 5 min in a platform shaker. Addition of 10 mL of ACN solution with 0.1% acetic acid, stirred for 1 min by a vortex and for 5 min in a platform shaker. Addition of citrate buffer salts (4 g MgSO4, 1 g NaCl, 1 g tri-sodium citrate, and 0.5 g sodium citrate dibasic sesquihydrate) and manually shaken for 10 s and vortex for 1 min. Ultrasonicated for 10 min and centrifuge (4000 rpm, 5 min).	[128]
PhACs (32)	0.2	Freeze-dried and homogenization	10		[129]
PhACs (17 antibiotics)	1	Frozen at at –20 °C. Air-dried, ground and sieved, frozen again until analyses	10+8+2	Vortex mixed for 15 s. Addition of citrate buffer salt (consisting of 4 g $MgSO_4$, 1.0 g NaCl, 1.0 g Na_3Cit, and 0.5 g Na_2Cit). Shaken in vortex for 1 min. Sonicated for 10 min and centrifuged at 3500 rpm for 10 min.	[130]
PPCPs (12)	10	Frozen at –18 °C until analysis		mix and hand-shaken with ACN by hand; vortex, addition of 4 g $MgSO_4$ + NaCl, mix manually 15 s; vortex (1 min); centrifugation (5000 rpm, 5 min).	[131]

PPCPs (21)	10	Frozen at –18 °C prior to analysis	10	Mix and hand-shaken with ACN by hand, vortex, addition of 4 g of $MgSO_4$ and NaCl, mix manually 15 s, vortex for 1 min, centrifugation (3400 rpm, 8 min)	[132]
PPCPs (more than 100)	-	Freeze-dried, crushed and sieved by 0.2 mm		Buffer salt (citrate buffering-salt) + solvent (ACN)	[133]
PPCPs and flame retardants (71)	0.1-2.5	Non-specified	-	In this study depends on the compounds an extraction method and detection techniques were used	[116]
PPCPs and metabolites (19)	2	Freeze-dried, homogenized, crushed and store at –18 °C prior to analysis	15	Vortex-mixed for 5 min, 4 g $MgSO_4$ and 1 g NaCl and vortex-mixed for 1 min. Centrifuged (2900 x g for 20 min)	[72][a]

PhACs: pharmaceutical compounds, PPCPs: pharmaceutical and personal care products, PCPs: personal care products NSAIDs: non-steroidal anti-inflammatory drugs. [1]Clean-up step was not applied for erythromycin and ciprofloxacin. [a]Different extraction methods were compared and validated.

Table S9. Details of the procedure of MSDP of EPs in sewage sludge**.**

Analytes	Sample mass (g)	Pre-treatment	Solvent volume (mL)	Extraction details	Ref.
Flame retardants/Plasticisers (8 organophosphates compounds)	0.5	Freeze-dried and stored at 4 °C until analysis	15	Mixed and dispersed with 2g of C18 with a pestle for 5 min; mixed with PSA as clean-up sorbent. Recover in 15 mL ACN	[134]

PCPs (TCS and MTCS)	0.5	Freeze-dried			[135]
PCPs (TCS and MTCS)	1	Freeze-dried	~13	Previously treated with C18-Na_2SO_4 (2:1, p/p)	[136]
PCPs (9)	1	Freeze-dried		C18 (2 g) and anhydrous sodium sulphate (1 g) were previously added, blended with a glass pestle and eluted in the solvent	[137]
PCPs (10 UV stabilizers)	0.5	Freeze-dried + diatomaceous earth mixed with a pestle	10	Transferred to polypropylene syringe with the clean-up sorbent	[138]
PCPs (8 azoles)	0.5	Freeze-dried	~10	Dispersed with C_{18} with a pestle for 5 min, added PSA to MSPD syringe, the sample were loaded above the PSA layer	[139]
PhACs (2 cardiac drug)	0.5	Freeze-dried	20	C_{18} (2 g) was added and mixed with a pestle and mortar for 5 min; MSPD syringes loaded with PSA (1 g) + Florisil and Na_2SO_4 (1 g) were previously	[140]
PhACs (5 NSAIDs)	0.2	Freeze-dried	15	soaked in KOH (60%, w/v), mixed with 1 g Na_2SO_4	[141]
PhACs (5 azoles)	0.5	Freeze-dried and maintained at 4 °C	10	2 g of C_{18} as dispersant	[142]
PPCPs (45)	0.1	Freeze-dried	6+10	0.4 g of C_{18}-bonded silica sorbent elution by extraction solvent	[143]
PPCPs (27)	2	Freeze-dried	5	Macerated (5 min) in a glass mortar, vortex (1 min), solvent addition, centrifugation (5 min, 11011 x g)	[144]
PPCPs (68)	0.5	Freeze-dried and store at		Mixed and dispersed with 2 g of C18 with a pestle for 5 min; mixed with PSA as clean-up sorbent. Recover in 15 mL ACN	[145]

4 °C until analyses

PCPs: personal care products, TCS: triclosan, MTCS: methyl triclosan, PhACs: pharmaceutical compounds, NSAIDs: non- steroidal anti-inflammatory drugs, Ref: references.

Table S10. Details of the procedure of different extraction techniques in sewage sludge**.**

Analytes	Sample mass (g)	Pre-treatment	Solvent volume (mL)	Extraction details	Ref.
Flame retardants (2 PCBs) by SLE-LTP	4	non-specified	~15	vortex (1 min), freeze aqueous phase (–18 °C, 1 h), addition of 375 mg of Na_2SO_4 (375 mg), vortex (1 min), centrifuged (4000 rpm, 10 min) kept at 4 °C until GC-MS	[146]
Flame retardants (6 PCBs 6) by SLE-LTP	4	non-specified	8	sonicated (15 min), incubated in a stirring water bath at 40 °C (1 h), frozen at –20 °C (2h)	[147]
PCPs (6 musks) by MA-HS-SPME	5	Stirring and filtrate by Whatman 934-AH glass fibre papers.	20	Stir at 300 rpm and vaporized HS MW 80 W 5 min	[148]
PCPs (8 macrocyclic musks) by HS-SPME	0.25	Freeze-dried and sieved by 125 µm	0.5	Heating/stirring at 80 °C, 1 min for stabilization, PDMS/DVB 65 µm was introduced 45 min at 750 rpm, inserted for desorption	[149]
Plasticisers (5 phthalates) by SLE-LTP	not specified	store in glass jars and stored at 4 °C. Prior to analysis were left 8 h RT, vortexed and sieved	4	solvent addition, freezing for 90 min at –20 °C, vortex 30 s	[150]

PhACs (4 NSAIDs) by HF-LPME	0.5-1	Refrigerate in closed bottle at 4 °C		solvent is acceptor phase:	[151]
PhACs (4 NSAIDs) by HF-LPME	0.5-1	Homogenised and diluted 10 times in water pH 2 (adjusted with sulfuric acid)		4 hours, stirring at 660 rpm	[152]
PhACs (3 metabolites from NSAIDs) by HF-LPME	2	stored refrigerated at 4 °C until analysis		Acceptor phase: 0.1 M ammonium carbonate (pH: 9); extraction time: 180 min; 440 rpm; fiber length: 6 cm; 5% of NaCl	[153]
PPCPs (6) by SBSE	0.5	Dried-sieved <1 mm stored at 4 °C	5	Addition of $NaHCO_3$ + insertion of stir bar with PDMS coating film + addition of acetic acid anhydride. Mix (1000 rpm, 60 min). The Twister was washed and dried for TD	[154]

PCPs: personal care products, PCBs: polychlorinated biphenyls, PhACs: pharmaceutical compounds, NSAIDs: non-steroidal anti-inflammatory drugs, MA-HS-SPME: microwave-assisted headspace solid-phase, HS-SPME: headspace solid-phase microextraction, SLE-LTP: solid–liquid extraction with low-temperature purification, HF-LPME: hollow fiber based-liquid phase micro extraction, SBSE: stir bar sorptive extraction. Ref: reference.

References

1. Novak, P.; Zuliani, T.; Milačič, R.; Ščančar, J. Development of an Analytical Method for the Determination of Polybrominated Diphenyl Ethers in Sewage Sludge by the Use of Gas Chromatography Coupled to Inductively Coupled Plasma Mass Spectrometry. *Analytica Chimica Acta* **2016**, *915*, 27–35, doi:10.1016/j.aca.2016.02.022.
2. Zhang, Z.; Ren, N.; Li, Y.-F.; Kunisue, T.; Gao, D.; Kannan, K. Determination of Benzotriazole and Benzophenone UV Filters in Sediment and Sewage Sludge. *Environ. Sci. Technol.* **2011**, *45*, 3909–3916, doi:10.1021/es2004057.
3. Zuliani, T.; Milačič, R.; Ščančar, J. Preparation of a Sewage Sludge Laboratory Quality Control Material for Butyltin Compounds and Their Determination by Isotope-Dilution Mass Spectrometry. *Anal Bioanal Chem* **2012**, *403*, 857–865, doi:10.1007/s00216-012-5897-7.
4. Gao, D.; Li, Z.; Wen, Z.; Ren, N. Occurrence and Fate of Phthalate Esters in Full-Scale Domestic Wastewater Treatment Plants and Their Impact on Receiving Waters along the Songhua River in China. *Chemosphere* **2014**, *95*, 24–32, doi:10.1016/j.chemosphere.2013.08.009.
5. Gani, K.M.; Kazmi, A.A. Comparative Assessment of Phthalate Removal and Risk in Biological Wastewater Treatment Systems of Developing Countries and Small Communities. *Science of The Total Environment* **2016**, *569–570*, 661–671, doi:10.1016/j.scitotenv.2016.06.182.
6. Gani, K.M.; Bux, F.; Kazmi, A.A. Diethylhexyl Phthalate Removal in Full Scale Activated Sludge Plants: Effect of Operational Parameters. *Chemosphere* **2019**, *234*, 885–892, doi:10.1016/j.chemosphere.2019.06.130.
7. Gani, K.M.; Kazmi, A.A. Ecotoxicological Risk Evaluation and Regulatory Compliance of Endocrine Disruptor Phthalates in a Sustainable Wastewater Treatment Scheme. *Environ Sci Pollut Res* **2020**, *27*, 7785–7794, doi:10.1007/s11356-019-07418-7.
8. Qian, Y.; Jia, X.; Ding, T.; Yang, M.; Yang, B.; Li, J. Occurrence and Removal of Bisphenol Analogues in Wastewater Treatment Plants and Activated Sludge Bioreactor. *Science of The Total Environment* **2021**, *758*, 143606, doi:10.1016/j.scitotenv.2020.143606.
9. Vrkoslavová, J.; Demnerová, K.; Macková, M.; Zemanová, T.; Macek, T.; Hajšlová, J.; Pulkrabová, J.; Hrádková, P.; Stiborová, H. Absorption and Translocation of Polybrominated Diphenyl Ethers (PBDEs) by Plants from Contaminated Sewage Sludge. *Chemosphere* **2010**, *81*, 381–386, doi:10.1016/j.chemosphere.2010.07.010.
10. Cincinelli, A.; Martellini, T.; Misuri, L.; Lanciotti, E.; Sweetman, A.; Laschi, S.; Palchetti, I. PBDEs in Italian Sewage Sludge and Environmental Risk of Using Sewage Sludge for Land Application. *Environmental Pollution* **2012**, *161*, 229–234, doi:10.1016/j.envpol.2011.11.001.
11. Zeng, X.-Y.; Cao, S.-X.; Zhang, D.-L.; Gao, S.-T.; Yu, Z.-Q.; Li, H.-R.; Sheng, G.-Y.; Fu, J.-M. Levels and Distribution of Synthetic Musks and Polycyclic Aromatic Hydrocarbons in Sludge Collected from Guangdong Province. *J Environ Sci Health A Tox Hazard Subst Environ Eng* **2012**, *47*, 389–397, doi:10.1080/10934529.2012.646099.
12. Gao, S.; Tian, B.; Zeng, X.; Yu, Z. Enantiomeric Analysis of Polycyclic Musks AHTN and HHCB and HHCB-Lactone in Sewage Sludge by Gas Chromatography/Tandem Mass Spectrometry. *Rapid Communications in Mass Spectrometry* **2019**, *33*, 607–612, doi:10.1002/rcm.8390.
13. Samaras, V.G.; Thomaidis, N.S.; Stasinakis, A.S.; Lekkas, T.D. An Analytical Method for the Simultaneous Trace Determination of Acidic Pharmaceuticals and Phenolic Endocrine Disrupting Chemicals in Wastewater and Sewage Sludge by Gas Chromatography-Mass Spectrometry. *Anal Bioanal Chem* **2011**, *399*, 2549–2561, doi:10.1007/s00216-010-4607-6.
14. Koumaki, E.; Noutsopoulos, C.; Mamais, D.; Fragkiskatos, G.; Andreadakis, A. Fate of Emerging Contaminants in High-Rate Activated Sludge Systems. *Int J Environ Res Public Health* **2021**, *18*, E400, doi:10.3390/ijerph18020400.
15. Golovko, O.; Örn, S.; Sörengård, M.; Frieberg, K.; Nassazzi, W.; Lai, F.Y.; Ahrens, L. Occurrence and Removal of Chemicals of Emerging Concern in Wastewater Treatment Plants and Their Impact on Receiving Water Systems. *Science of The Total Environment* **2021**, *754*, 142122, doi:https://doi.org/10.1016/j.scitotenv.2020.142122.
16. Meng, Y.; Liu, W.; Fiedler, H.; Zhang, J.; Wei, X.; Liu, X.; Peng, M.; Zhang, T. Fate and Risk Assessment of Emerging Contaminants in Reclaimed Water Production Processes. *Front. Environ. Sci. Eng.* **2021**, *15*, 104, doi:10.1007/s11783-021-1392-8.
17. Santana, J.M.; Fraga, S.V.B.; Zanatta, M.C.K.; Martins, M.R.; Pires, M.S.G. Characterization of Organic Compounds and Drugs in Sewage Sludge Aiming for Agricultural Recycling. *Heliyon* **2021**, *7*, e06771, doi:10.1016/j.heliyon.2021.e06771.
18. Wilschnack, K.; Homer, B.; Cartmell, E.; Yates, K.; Petrie, B. Targeted Multi-Analyte UHPLC-MS/MS Methodology for Emerging Contaminants in Septic Tank Wastewater, Sludge and Receiving Surface Water. *Anal. Methods* **2024**, *16*, 709–720, doi:10.1039/D3AY01201H.

19. Álvarez-Ruiz, R.; Andrés-Costa, M.J.; Andreu, V.; Picó, Y. Simultaneous Determination of Traditional and Emerging Illicit Drugs in Sediments, Sludges and Particulate Matter. *Journal of Chromatography A* **2015**, *1405*, 103–115, doi:10.1016/j.chroma.2015.05.062.
20. Guerra, P.; Eljarrat, E.; Barceló, D. Simultaneous Determination of Hexabromocyclododecane, Tetrabromobisphenol A, and Related Compounds in Sewage Sludge and Sediment Samples from Ebro River Basin (Spain). *Anal Bioanal Chem* **2010**, *397*, 2817–2824, doi:10.1007/s00216-010-3670-3.
21. Navarro, I.; Sanz, P.; Martínez, M.Á. Analysis of Perfluorinated Alkyl Substances in Spanish Sewage Sludge by Liquid Chromatography–Tandem Mass Spectrometry. *Anal Bioanal Chem* **2011**, *400*, 1277–1286, doi:10.1007/s00216-011-4655-6.
22. Cristale, J.; Lacorte, S. Development and Validation of a Multiresidue Method for the Analysis of Polybrominated Diphenyl Ethers, New Brominated and Organophosphorus Flame Retardants in Sediment, Sludge and Dust. *Journal of Chromatography A* **2013**, *1305*, 267–275, doi:10.1016/j.chroma.2013.07.028.
23. Abril, C.; Santos, J.L.; Malvar, J.L.; Martín, J.; Aparicio, I.; Alonso, E. Determination of Perfluorinated Compounds, Bisphenol A, Anionic Surfactants and Personal Care Products in Digested Sludge, Compost and Soil by Liquid-Chromatography-Tandem Mass Spectrometry. *Journal of Chromatography A* **2018**, *1576*, 34–41, doi:10.1016/j.chroma.2018.09.028.
24. Demirtepe, H.; Imamoglu, I. Levels of Polybrominated Diphenyl Ethers and Hexabromocyclododecane in Treatment Plant Sludge: Implications on Sludge Management. *Chemosphere* **2019**, *221*, 606–615, doi:10.1016/j.chemosphere.2019.01.060.
25. Martínez-Moral, M.P.; Tena, M.T. Focused Ultrasound Solid–Liquid Extraction of Perfluorinated Compounds from Sewage Sludge. *Talanta* **2013**, *109*, 197–202, doi:10.1016/j.talanta.2013.02.020.
26. Košnář, Z.; Mercl, F.; Pierdonà, L.; Chane, A.D.; Míchal, P.; Tlustoš, P. Concentration of the Main Persistent Organic Pollutants in Sewage Sludge in Relation to Wastewater Treatment Plant Parameters and Sludge Stabilisation. *Environmental Pollution* **2023**, *333*, 122060, doi:10.1016/j.envpol.2023.122060.
27. Liu, R.; Ruan, T.; Wang, T.; Song, S.; Yu, M.; Gao, Y.; Shao, J.; Jiang, G. Trace Analysis of Mono-, Di-, Tri-Substituted Polyfluoroalkyl Phosphates and Perfluorinated Phosphonic Acids in Sewage Sludge by High Performance Liquid Chromatography Tandem Mass Spectrometry. *Talanta* **2013**, *111*, 170–177, doi:10.1016/j.talanta.2013.02.063.
28. Chokwe, T.B.; Okonkwo, J.O.; Sibali, L.L.; Ncube, E.J. An Integrated Method for the Simultaneous Determination of Alkylphenol Ethoxylates and Brominated Flame Retardants in Sewage Sludge Samples by Ultrasonic-Assisted Extraction, Solid Phase Clean-up, and GC-MS Analysis. *Microchemical Journal* **2015**, *123*, 230–236, doi:10.1016/j.microc.2015.07.001.
29. Zacs, D.; Bartkevics, V. Trace Determination of Perfluorooctane Sulfonate and Perfluorooctanoic Acid in Environmental Samples (Surface Water, Wastewater, Biota, Sediments, and Sewage Sludge) Using Liquid Chromatography – Orbitrap Mass Spectrometry. *Journal of Chromatography A* **2016**, *1473*, 109–121, doi:10.1016/j.chroma.2016.10.060.
30. Zhao, M.; Yao, Y.; Dong, X.; Fang, B.; Wang, Z.; Chen, H.; Sun, H. Identification of Emerging PFAS in Industrial Sludge from North China: Release Risk Assessment by the TOP Assay. *Water Research* **2025**, *268*, 122667, doi:10.1016/j.watres.2024.122667.
31. Huang, Q.; Yu, Y.; Tang, C.; Peng, X. Determination of Commonly Used Azole Antifungals in Various Waters and Sewage Sludge Using Ultra-High Performance Liquid Chromatography-Tandem Mass Spectrometry. *J Chromatogr A* **2010**, *1217*, 3481–3488, doi:10.1016/j.chroma.2010.03.022.
32. Clara, M.; Gans, O.; Windhofer, G.; Krenn, U.; Hartl, W.; Braun, K.; Scharf, S.; Scheffknecht, C. Occurrence of Polycyclic Musks in Wastewater and Receiving Water Bodies and Fate during Wastewater Treatment. *Chemosphere* **2011**, *82*, 1116–1123, doi:10.1016/j.chemosphere.2010.11.041.
33. Viglino, L.; Prévost, M.; Sauvé, S. High Throughput Analysis of Solid-Bound Endocrine Disruptors by LDTD-APCI-MS/MS. *J. Environ. Monit.* **2011**, *13*, 583–590, doi:10.1039/C0EM00550A.
34. Chen, Z.-F.; Ying, G.-G.; Lai, H.-J.; Chen, F.; Su, H.-C.; Liu, Y.-S.; Peng, F.-Q.; Zhao, Jian.-L. Determination of Biocides in Different Environmental Matrices by Use of Ultra-High-Performance Liquid Chromatography–Tandem Mass Spectrometry. *Anal Bioanal Chem* **2012**, *404*, 3175–3188, doi:10.1007/s00216-012-6444-2.
35. Healy, M.G.; Fenton, O.; Cormican, M.; Peyton, D.P.; Ordsmith, N.; Kimber, K.; Morrison, L. Antimicrobial Compounds (Triclosan and Triclocarban) in Sewage Sludges, and Their Presence in Runoff Following Land Application. *Ecotoxicology and Environmental Safety* **2017**, *142*, 448–453, doi:10.1016/j.ecoenv.2017.04.046.
36. Zhou, G.-J.; Li, X.-Y.; Leung, K.M.Y. Retinoids and Oestrogenic Endocrine Disrupting Chemicals in Saline Sewage Treatment Plants: Removal Efficiencies and Ecological Risks to Marine Organisms. *Environment International* **2019**, *127*, 103–113, doi:10.1016/j.envint.2019.03.030.

37. Zhou, G.-J.; Lin, L.; Li, X.-Y.; Leung, K.M.Y. Removal of Emerging Contaminants from Wastewater during Chemically Enhanced Primary Sedimentation and Acidogenic Sludge Fermentation. *Water Research* **2020**, *175*, 115646, doi:10.1016/j.watres.2020.115646.
38. Kor-Bicakci, G.; Abbott, T.; Ubay-Cokgor, E.; Eskicioglu, C. Occurrence of the Persistent Antimicrobial Triclosan in Microwave Pretreated and Anaerobically Digested Municipal Sludges under Various Process Conditions. *Molecules* **2020**, *25*, 310, doi:10.3390/molecules25020310.
39. Tasselli, S.; Guzzella, L. Polycyclic Musk Fragrances (PMFs) in Wastewater and Activated Sludge: Analytical Protocol and Application to a Real Case Study. *Environ Sci Pollut Res* **2020**, *27*, 30977–30986, doi:10.1007/s11356-019-06767-7.
40. Wang, Y.; Teng, Y.; Wang, D.; Han, K.; Wang, H.; Kang, L. The Fate of Triclocarban in Activated Sludge and Its Influence on Biological Wastewater Treatment System. *Journal of Environmental Management* **2020**, *276*, 111237, doi:10.1016/j.jenvman.2020.111237.
41. Abbott, T.; Eskicioglu, C. Comparison of Anaerobic, Cycling Aerobic/Anoxic, and Sequential Anaerobic/Aerobic/Anoxic Digestion to Remove Triclosan and Triclosan Metabolites from Municipal Biosolids. *Science of The Total Environment* **2020**, *745*, 140953, doi:https://doi.org/10.1016/j.scitotenv.2020.140953.
42. Košnář, Z.; Mercl, F.; Chane, A.D.; Pierdonà, L.; Míchal, P.; Tlustoš, P. Occurrence of Synthetic Polycyclic and Nitro Musk Compounds in Sewage Sludge from Municipal Wastewater Treatment Plants. *Science of The Total Environment* **2021**, *801*, 149777, doi:10.1016/j.scitotenv.2021.149777.
43. Martín, J.; Santos, J.L.; Aparicio, I.; Alonso, E. Multi-Residue Method for the Analysis of Pharmaceutical Compounds in Sewage Sludge, Compost and Sediments by Sonication-Assisted Extraction and LC Determination. *Journal of Separation Science* **2010**, *33*, 1760–1766, doi:10.1002/jssc.200900873.
44. Liu, S.-S.; Ying, G.-G.; Liu, S.; Lai, H.-J.; Chen, Z.-F.; Pan, C.-G.; Zhao, J.-L.; Chen, J. Analysis of 21 Progestagens in Various Matrices by Ultra-High-Performance Liquid Chromatography Tandem Mass Spectrometry (UHPLC-MS/MS) with Diverse Sample Pretreatment. *Anal Bioanal Chem* **2014**, *406*, 7299–7311, doi:10.1007/s00216-014-8146-4.
45. Lajeunesse, A.; Smyth, S.A.; Barclay, K.; Sauvé, S.; Gagnon, C. Distribution of Antidepressant Residues in Wastewater and Biosolids Following Different Treatment Processes by Municipal Wastewater Treatment Plants in Canada. *Water Research* **2012**, *46*, 5600–5612, doi:10.1016/j.watres.2012.07.042.
46. Shafrir, M.; Avisar, D. Development Method for Extracting and Analyzing Antibiotic and Hormone Residues from Treated Wastewater Sludge and Composted Biosolids. *Water Air Soil Pollut* **2012**, *223*, 2571–2587, doi:10.1007/s11270-011-1049-5.
47. Zhou, L.-J.; Ying, G.-G.; Liu, S.; Zhao, J.-L.; Chen, F.; Zhang, R.-Q.; Peng, F.-Q.; Zhang, Q.-Q. Simultaneous Determination of Human and Veterinary Antibiotics in Various Environmental Matrices by Rapid Resolution Liquid Chromatography–Electrospray Ionization Tandem Mass Spectrometry. *Journal of Chromatography A* **2012**, *1244*, 123–138, doi:10.1016/j.chroma.2012.04.076.
48. Dorival-García, N.; Zafra-Gómez, A.; Camino-Sánchez, F.J.; Navalón, A.; Vílchez, J.L. Analysis of Quinolone Antibiotic Derivatives in Sewage Sludge Samples by Liquid Chromatography–Tandem Mass Spectrometry: Comparison of the Efficiency of Three Extraction Techniques. *Talanta* **2013**, *106*, 104–118, doi:10.1016/j.talanta.2012.11.080.
49. Garcia-Rodríguez, A.; Sagristà, E.; Matamoros, V.; Fontàs, C.; Hidalgo, M.; Salvadó, V. Determination of Pharmaceutical Compounds in Sewage Sludge Using a Standard Addition Method Approach. *International Journal of Environmental Analytical Chemistry* **2014**, *94*, 1199–1209, doi:10.1080/03067319.2014.921292.
50. Yuan, X.; Qiang, Z.; Ben, W.; Zhu, B.; Liu, J. Rapid Detection of Multiple Class Pharmaceuticals in Both Municipal Wastewater and Sludge with Ultra High Performance Liquid Chromatography Tandem Mass Spectrometry. *Journal of Environmental Sciences* **2014**, *26*, 1949–1959, doi:10.1016/j.jes.2014.06.022.
51. Boix, C.; Ibáñez, M.; Fabregat-Safont, D.; Morales, E.; Pastor, L.; Sancho, J.V.; Sánchez-Ramírez, J.E.; Hernández, F. Analytical Methodologies Based on LC–MS/MS for Monitoring Selected Emerging Compounds in Liquid and Solid Phases of the Sewage Sludge. *MethodsX* **2016**, *3*, 333–342, doi:10.1016/j.mex.2016.04.010.
52. Ferhi, S.; Bourdat-Deschamps, M.; Daudin, J.-J.; Houot, S.; Nélieu, S. Factors Influencing the Extraction of Pharmaceuticals from Sewage Sludge and Soil: An Experimental Design Approach. *Anal Bioanal Chem* **2016**, *408*, 6153–6168, doi:10.1007/s00216-016-9725-3.
53. Zhang, M.; Mao, Q.; Feng, J.; Yuan, S.; Wang, Q.; Huang, D.; Zhang, J. Validation and Application of an Analytical Method for the Determination of Selected Acidic Pharmaceuticals and Estrogenic

Hormones in Wastewater and Sludge. *Journal of Environmental Science and Health, Part A* **2016**, *51*, 914–920, doi:10.1080/10934529.2016.1191304.
54. Martínez-Alcalá, I.; Guillén-Navarro, J.M.; Fernández-López, C. Pharmaceutical Biological Degradation, Sorption and Mass Balance Determination in a Conventional Activated-Sludge Wastewater Treatment Plant from Murcia, Spain. *Chemical Engineering Journal* **2017**, *316*, 332–340, doi:https://doi.org/10.1016/j.cej.2017.01.048.
55. Lindholm-Lehto, P.C.; Ahkola, H.S.J.; Knuutinen, J.S. Pharmaceuticals in Processing of Municipal Sewage Sludge Studied by Grab and Passive Sampling. *Water Quality Research Journal* **2018**, *53*, 14–23, doi:10.2166/wqrj.2018.022.
56. Abbott, T.; Kor-Bicakci, G.; Eskicioglu, C. Examination of Single-Stage Anaerobic and Anoxic/Aerobic and Dual-Stage Anaerobic-Anoxic/Aerobic Digestion to Remove Pharmaceuticals from Municipal Biosolids. *Science of The Total Environment* **2021**, *791*, 148237, doi:10.1016/j.scitotenv.2021.148237.
57. Martínez-Alcalá, I.; Guillén-Navarro, J.M.; Lahora, A. Occurrence and Fate of Pharmaceuticals in a Wastewater Treatment Plant from Southeast of Spain and Risk Assessment. *Journal of Environmental Management* **2021**, *279*, 111565, doi:https://doi.org/10.1016/j.jenvman.2020.111565.
58. Mercl, F.; Košnář, Z.; Maršík, P.; Vojtíšek, M.; Dušek, J.; Száková, J.; Tlustoš, P. Pyrolysis of Biosolids as an Effective Tool to Reduce the Uptake of Pharmaceuticals by Plants. *Journal of Hazardous Materials* **2021**, *405*, doi:10.1016/j.jhazmat.2020.124278.
59. Gago-Ferrero, P.; Borova, V.; Dasenaki, M.E.; Thomaidis, N.S. Simultaneous Determination of 148 Pharmaceuticals and Illicit Drugs in Sewage Sludge Based on Ultrasound-Assisted Extraction and Liquid Chromatography–Tandem Mass Spectrometry. *Anal Bioanal Chem* **2015**, *407*, 4287–4297, doi:10.1007/s00216-015-8540-6.
60. Mejías, C.; Santos, J.L.; Martín, J.; Aparicio, I.; Alonso, E. Multiresidue Method for the Determination of Critically and Highly Important Classes of Antibiotics and Their Metabolites in Agricultural Soils and Sewage Sludge. *Anal Bioanal Chem* **2023**, *415*, 7161–7173, doi:10.1007/s00216-023-04982-3.
61. Fernández-Sanjuan, M.; Lacorte, S.; Rigol, A.; Sahuquillo, A. New Quality-Control Materials for the Determination of Alkylphenols and Alkylphenol Ethoxylates in Sewage Sludge. *Anal Bioanal Chem* **2012**, *404*, 2499–2505, doi:10.1007/s00216-012-6342-7.
62. Yang, Y.; Lu, L.; Zhang, J.; Yang, Y.; Wu, Y.; Shao, B. Simultaneous Determination of Seven Bisphenols in Environmental Water and Solid Samples by Liquid Chromatography–Electrospray Tandem Mass Spectrometry. *Journal of Chromatography A* **2014**, *1328*, 26–34, doi:10.1016/j.chroma.2013.12.074.
63. Ömeroğlu, S.; Kara Murdoch, F.; Dilek Sanin, F. Investigation of Nonylphenol and Nonylphenol Ethoxylates in Sewage Sludge Samples from a Metropolitan Wastewater Treatment Plant in Turkey. *Talanta* **2015**, *131*, 650–655, doi:10.1016/j.talanta.2014.08.014.
64. Sun, X.; Peng, J.; Wang, M.; Wang, J.; Tang, C.; Yang, L.; Lei, H.; Li, F.; Wang, X.; Chen, J. Determination of Nine Bisphenols in Sewage and Sludge Using Dummy Molecularly Imprinted Solid-Phase Extraction Coupled with Liquid Chromatography Tandem Mass Spectrometry. *Journal of Chromatography A* **2018**, *1552*, 10–16, doi:10.1016/j.chroma.2018.04.004.
65. García-Valcárcel, A.I.; Tadeo, J.L. Determination of Azoles in Sewage Sludge from Spanish Wastewater Treatment Plants by Liquid Chromatography-Tandem Mass Spectrometry. *J Sep Sci* **2011**, *34*, 1228–1235, doi:10.1002/jssc.201000814.
66. Yu, Y.; Huang, Q.; Cui, J.; Zhang, K.; Tang, C.; Peng, X. Determination of Pharmaceuticals, Steroid Hormones, and Endocrine-Disrupting Personal Care Products in Sewage Sludge by Ultra-High-Performance Liquid Chromatography-Tandem Mass Spectrometry. *Anal Bioanal Chem* **2011**, *399*, 891–902, doi:10.1007/s00216-010-4295-2.
67. Huang, Q.; Zhang, K.; Wang, Z.; Wang, C.; Peng, X. Enantiomeric Determination of Azole Antifungals in Wastewater and Sludge by Liquid Chromatography–Tandem Mass Spectrometry. *Anal Bioanal Chem* **2012**, *403*, 1751–1760, doi:10.1007/s00216-012-5976-9.
68. Tang, C.; Yu, Y.; Huang, Q.; Peng, X. Simultaneous Determination of Fluoroquinolone and Tetracycline Antibacterials in Sewage Sludge Using Ultrasonic-Assisted Extraction and HPLC-MS/MS. *International Journal of Environmental Analytical Chemistry* **2012**, *92*, 1389–1402, doi:10.1080/03067319.2010.535124.
69. Yu, Y.; Wu, L. Analysis of Endocrine Disrupting Compounds, Pharmaceuticals and Personal Care Products in Sewage Sludge by Gas Chromatography–Mass Spectrometry. *Talanta* **2012**, *89*, 258–263, doi:10.1016/j.talanta.2011.12.023.
70. Hajj-Mohamad, M.; Aboulfadl, K.; Darwano, H.; Madoux-Humery, A.-S.; Guérineau, H.; Sauvé, S.; Prévost, M.; Dorner, S. Wastewater Micropollutants as Tracers of Sewage Contamination: Analysis of Combined Sewer Overflow and Stream Sediments. *Environmental Science: Processes & Impacts*

2014, *16*, 2442–2450, doi:10.1039/C4EM00314D.
71. López-Serna, R.; Marín-de-Jesús, D.; Irusta-Mata, R.; García-Encina, P.A.; Lebrero, R.; Fdez-Polanco, M.; Muñoz, R. Multiresidue Analytical Method for Pharmaceuticals and Personal Care Products in Sewage and Sewage Sludge by Online Direct Immersion SPME On-Fiber Derivatization – GCMS. *Talanta* **2018**, *186*, 506–512, doi:10.1016/j.talanta.2018.04.099.
72. Malvar, J.L.; Santos, J.L.; Martín, J.; Aparicio, I.; Alonso, E. Comparison of Ultrasound-Assisted Extraction, QuEChERS and Selective Pressurized Liquid Extraction for the Determination of Metabolites of Parabens and Pharmaceuticals in Sludge. *Microchemical Journal* **2020**, *157*, 104987, doi:10.1016/j.microc.2020.104987.
73. Method 1694: Pharmaceuticals and Personal Care Products in Water, Soil, Sediment, and Biosolids by HPLC/MS/MS. **2007**, 77.
74. US EPA, U. *Method 3550C–Ultrasonic Extraction*; Environmental Protection Agency of United States EPA, Washington, Virginia, USA, 2000;
75. Vega-Morales, T.; Sosa-Ferrera, Z.; Santana-Rodríguez, J.J. Determination of Various Estradiol Mimicking-Compounds in Sewage Sludge by the Combination of Microwave-Assisted Extraction and LC–MS/MS. *Talanta* **2011**, *85*, 1825–1834, doi:10.1016/j.talanta.2011.07.051.
76. Azzouz, A.; Ballesteros, E. Determination of 13 Endocrine Disrupting Chemicals in Environmental Solid Samples Using Microwave-Assisted Solvent Extraction and Continuous Solid-Phase Extraction Followed by Gas Chromatography–Mass Spectrometry. *Anal Bioanal Chem* **2016**, *408*, 231–241, doi:10.1007/s00216-015-9096-1.
77. Guedes-Alonso, R.; Santana-Viera, S.; Montesdeoca-Esponda, S.; Afonso-Olivares, C.; Sosa-Ferrera, Z.; Santana-Rodríguez, J.J. Application of Microwave-Assisted Extraction and Ultra-High Performance Liquid Chromatography–Tandem Mass Spectrometry for the Analysis of Sex Hormones and Corticosteroids in Sewage Sludge Samples. *Anal Bioanal Chem* **2016**, *408*, 6833–6844, doi:10.1007/s00216-016-9810-7.
78. Lee, H.-B.; Lewina Svoboda, M.; Peart, T.E.; Smyth, S.A. Optimization of a Microwave-Assisted Extraction Procedure for the Determination of Selected Alkyl, Aryl, and Halogenated Phenols in Sewage Sludge and Biosolids. *Water Quality Research Journal* **2016**, *51*, 344–356, doi:10.2166/wqrjc.2016.002.
79. Banihashemi, B.; Droste, R.L. Trace Level Determination of Bisphenol-A in Wastewater and Sewage Sludge by High-Performance Liquid Chromatography and UV Detection. *Water Quality Research Journal* **2013**, *48*, 133–144, doi:10.2166/wqrjc.2013.037.
80. Devault, D.A.; Amalric, L.; Bristeau, S.; Cruz, J.; Tapie, N.; Karolak, S.; Budzinski, H.; Lévi, Y. Removal Efficiency of Emerging Micropollutants in Biofilter Wastewater Treatment Plants in Tropical Areas. *Environ Sci Pollut Res Int* **2021**, *28*, 10940–10966, doi:10.1007/s11356-020-10868-z.
81. Cantarero, S.; Zafra-Gómez, A.; Ballesteros, O.; Navalón, A.; Vílchez, J.L.; Verge, C.; De Ferrer, J.A. Matrix Effect Study in the Determination of Linear Alkylbenzene Sulfonates in Sewage Sludge Samples. *Environ. Toxicol. Chem.* **2011**, *30*, 813–818, doi:10.1002/etc.447.
82. Dobor, J.; Varga, M.; Yao, J.; Chen, H.; Palkó, G.; Záray, G. A New Sample Preparation Method for Determination of Acidic Drugs in Sewage Sludge Applying Microwave Assisted Solvent Extraction Followed by Gas Chromatography–Mass Spectrometry. *Microchemical Journal* **2010**, *94*, 36–41, doi:10.1016/j.microc.2009.08.007.
83. Montesdeoca-Esponda, S.; Sosa-Ferrera, Z.; Santana-Rodríguez, J.J. Combination of Microwave-Assisted Micellar Extraction with Liquid Chromatography Tandem Mass Spectrometry for the Determination of Fluoroquinolone Antibiotics in Coastal Marine Sediments and Sewage Sludges Samples. *Biomedical Chromatography* **2012**, *26*, 33–40, doi:10.1002/bmc.1621.
84. Evans, S.E.; Davies, P.; Lubben, A.; Kasprzyk-Hordern, B. Determination of Chiral Pharmaceuticals and Illicit Drugs in Wastewater and Sludge Using Microwave Assisted Extraction, Solid-Phase Extraction and Chiral Liquid Chromatography Coupled with Tandem Mass Spectrometry. *Analytica Chimica Acta* **2015**, *882*, 112–126, doi:https://doi.org/10.1016/j.aca.2015.03.039.
85. Junior, I.L.C.; Machado, C.S.; Pletsch, A.L.; Torres, Y.R. Simultaneous HPLC-PDA Determination of Commonly Prescribed Antidepressants and Caffeine in Sludge from Sewage Treatment Plants and River Sediments in the Itaipu Reservoir Region, Paraná, Brazil. *International Journal of Environmental Analytical Chemistry* **2020**, *100*, 1004–1020, doi:10.1080/03067319.2019.1646738.
86. Junior, I.L.C.; Machado, C.S.; Ramalho, A.N.; Pletsch, A.L.; Torres, Y.R. Optimisation of Caffeine and Antidepressants Extraction from Sediments and Sewage Sludge Using Experimental Designs. *International Journal of Environmental Analytical Chemistry* **2017**, *97*, 935–948, doi:10.1080/03067319.2017.1373772.
87. Zhou, S.; Peng, S.; Li, Z.; Zhang, D.; Zhu, Y.; Li, X.; Hong, M.; Li, W.; Lu, P. Risk Assessment of

Pollutants in Flowback and Produced Waters and Sludge in Impoundments. *Science of The Total Environment* **2022**, *811*, 152250, doi:10.1016/j.scitotenv.2021.152250.
88. Azzouz, A.; Ballesteros, E. Combined Microwave-Assisted Extraction and Continuous Solid-Phase Extraction Prior to Gas Chromatography–Mass Spectrometry Determination of Pharmaceuticals, Personal Care Products and Hormones in Soils, Sediments and Sludge. *Science of The Total Environment* **2012**, *419*, 208–215, doi:10.1016/j.scitotenv.2011.12.058.
89. Petrie, B.; Youdan, J.; Barden, R.; Kasprzyk-Hordern, B. Multi-Residue Analysis of 90 Emerging Contaminants in Liquid and Solid Environmental Matrices by Ultra-High-Performance Liquid Chromatography Tandem Mass Spectrometry. *Journal of Chromatography A* **2016**, *1431*, 64–78, doi:10.1016/j.chroma.2015.12.036.
90. Rhodes, L. Microwave-Assisted Extraction Using US EPA Method 3546. *LC GC NORTH AMERICA* **2002**, *20*, 23–23.
91. Gorga, M.; Insa, S.; Petrovic, M.; Barceló, D. Analysis of Endocrine Disrupters and Related Compounds in Sediments and Sewage Sludge Using On-Line Turbulent Flow Chromatography–Liquid Chromatography–Tandem Mass Spectrometry. *Journal of Chromatography A* **2014**, *1352*, 29–37, doi:10.1016/j.chroma.2014.05.028.
92. Riva, F.; Zuccato, E.; Pacciani, C.; Colombo, A.; Castiglioni, S. A Multi-Residue Analytical Method for Extraction and Analysis of Pharmaceuticals and Other Selected Emerging Contaminants in Sewage Sludge. *Anal. Methods* **2021**, *13*, 526–535, doi:10.1039/D0AY02027C.
93. Arbeláez, P.; Granados, J.; Borrull, F.; Marcé, R.M.; Pocurull, E. Determination of Sedative Hypnotics in Sewage Sludge by Pressurized Liquid Extraction with High-Performance Liquid Chromatography and Tandem Mass Spectrometry. *Journal of Separation Science* **2014**, *37*, 3481–3488, doi:10.1002/jssc.201400791.
94. Mascolo, G.; Locaputo, V.; Mininni, G. New Perspective on the Determination of Flame Retardants in Sewage Sludge by Using Ultrahigh Pressure Liquid Chromatography-Tandem Mass Spectrometry with Different Ion Sources. *J Chromatogr A* **2010**, *1217*, 4601–4611, doi:10.1016/j.chroma.2010.05.003.
95. De la Torre, A.; Concejero, M.A.; Martínez, M.A. Concentrations and Sources of an Emerging Pollutant, Decabromodiphenylethane (DBDPE), in Sewage Sludge for Land Application. *J Environ Sci (China)* **2012**, *24*, 558–563, doi:10.1016/s1001-0742(11)60801-2.
96. Martínez-Moral, M.P.; Tena, M.T. Use of Microextraction by Packed Sorbents Following Selective Pressurised Liquid Extraction for the Determination of Brominated Diphenyl Ethers in Sewage Sludge by Gas Chromatography–Mass Spectrometry. *Journal of Chromatography A* **2014**, *1364*, 28–35, doi:10.1016/j.chroma.2014.08.075.
97. Veenaas, C.; Haglund, P. Methodology for Non-Target Screening of Sewage Sludge Using Comprehensive Two-Dimensional Gas Chromatography Coupled to High-Resolution Mass Spectrometry. *Anal Bioanal Chem* **2017**, *409*, 4867–4883, doi:10.1007/s00216-017-0429-0.
98. Zhang, Y.; Zhao, B.; Chen, Q.; Zhu, F.; Wang, J.; Fu, X.; Zhou, T. Fate of Organophosphate Flame Retardants (OPFRs) in the "Cambi® TH + AAD" of Sludge in a WWTP in Beijing, China. *Waste Management* **2023**, *169*, 363–373, doi:10.1016/j.wasman.2023.07.030.
99. Esparza, X.; Moyano, E.; de Boer, J.; Galceran, M.T.; van Leeuwen, S.P.J. Analysis of Perfluorinated Phosponic Acids and Perfluorooctane Sulfonic Acid in Water, Sludge and Sediment by LC–MS/MS. *Talanta* **2011**, *86*, 329–336, doi:10.1016/j.talanta.2011.09.024.
100. Llorca, M.; Farré, M.; Picó, Y.; Barceló, D. Analysis of Perfluorinated Compounds in Sewage Sludge by Pressurized Solvent Extraction Followed by Liquid Chromatography–Mass Spectrometry. *Journal of Chromatography A* **2011**, *1218*, 4840–4846, doi:10.1016/j.chroma.2011.01.085.
101. Mastroianni, N.; Postigo, C.; de Alda, M.L.; Barcelo, D. Illicit and Abused Drugs in Sewage Sludge: Method Optimization and Occurrence. *Journal of Chromatography A* **2013**, *1322*, 29–37, doi:10.1016/j.chroma.2013.10.078.
102. Vallecillos, L.; Borrull, F.; Pocurull, E. Determination of Musk Fragrances in Sewage Sludge by Pressurized Liquid Extraction Coupled to Automated Ionic Liquid-Based Headspace Single-Drop Microextraction Followed by GC-MS/MS. *Journal of Separation Science* **2012**, *35*, 2735–2742, doi:10.1002/jssc.201200326.
103. Tohidi, F.; Cai, Z. GC/MS Analysis of Triclosan and Its Degradation by-Products in Wastewater and Sludge Samples from Different Treatments. *Environ Sci Pollut Res* **2015**, *22*, 11387–11400, doi:10.1007/s11356-015-4289-x.
104. Scheurer, M.; Ramil, M.; Metcalfe, C.D.; Groh, S.; Ternes, T.A. The Challenge of Analyzing Beta-Blocker Drugs in Sludge and Wastewater. *Anal Bioanal Chem* **2010**, *396*, 845–856, doi:10.1007/s00216-009-3225-7.
105. Gabet-Giraud, V.; Miege, C.; Herbreteau, B.; Hernandez-Raquet, G.; Coquery, M. Development and

Validation of an Analytical Method by LC-MS/MS for the Quantification of Estrogens in Sewage Sludge. *Anal Bioanal Chem* **2010**, *396*, 1841–1851, doi:10.1007/s00216-009-3428-y.
106. Mohapatra, D.P.; Brar, S.K.; Tyagi, R.D.; Picard, P.; Surampalli, R.Y. Carbamazepine in Municipal Wastewater and Wastewater Sludge: Ultrafast Quantification by Laser Diode Thermal Desorption-Atmospheric Pressure Chemical Ionization Coupled with Tandem Mass Spectrometry. *Talanta* **2012**, *99*, 247–255, doi:10.1016/j.talanta.2012.05.047.
107. Yongshan Chen; Cao, Q.; Deng, S.; Huang, J.; Wang, B.; Yu, G. Determination of Pharmaceuticals from Various Therapeutic Classes in Dewatered Sludge by Pressurized Liquid Extraction and High Performance Liquid Chromatography and Tandem Mass Spectrometry (HPLC-MS/MS). *International Journal of Environmental Analytical Chemistry* **2013**, *93*, 1159–1173, doi:10.1080/03067319.2012.717271.
108. García-Galán, M.J.; Díaz-Cruz, S.; Barceló, D. Multiresidue Trace Analysis of Sulfonamide Antibiotics and Their Metabolites in Soils and Sewage Sludge by Pressurized Liquid Extraction Followed by Liquid Chromatography–Electrospray-Quadrupole Linear Ion Trap Mass Spectrometry. *Journal of Chromatography A* **2013**, *1275*, 32–40, doi:10.1016/j.chroma.2012.12.004.
109. Herrero, P.; Borrull, F.; Marcé, R.M.; Pocurull, E. Pressurised Liquid Extraction and Ultra-High Performance Liquid Chromatography-Tandem Mass Spectrometry to Determine Endogenous and Synthetic Glucocorticoids in Sewage Sludge. *Talanta* **2013**, *103*, 186–193, doi:10.1016/j.talanta.2012.10.030.
110. Seira, J.; Claparols, C.; Joannis-Cassan, C.; Albasi, C.; Montréjaud-Vignoles, M.; Sablayrolles, C. Optimization of Pressurized Liquid Extraction Using a Multivariate Chemometric Approach for the Determination of Anticancer Drugs in Sludge by Ultra High Performance Liquid Chromatography–Tandem Mass Spectrometry. *Journal of Chromatography A* **2013**, *1283*, 27–38, doi:10.1016/j.chroma.2013.01.114.
111. Arbeláez, P.; Borrull, F.; Maria Marcé, R.; Pocurull, E. Trace-Level Determination of Sweeteners in Sewage Sludge Using Selective Pressurized Liquid Extraction and Liquid Chromatography–Tandem Mass Spectrometry. *Journal of Chromatography A* **2015**, *1408*, 15–21, doi:10.1016/j.chroma.2015.07.001.
112. Salvia, M.-V.; Fieu, M.; Vulliet, E. Determination of Tetracycline and Fluoroquinolone Antibiotics at Trace Levels in Sludge and Soil Available online: https://www.hindawi.com/journals/aess/2015/435741/ (accessed on 23 April 2020).
113. vom Eyser, C.; Palmu, K.; Otterpohl, R.; Schmidt, T.C.; Tuerk, J. Determination of Pharmaceuticals in Sewage Sludge and Biochar from Hydrothermal Carbonization Using Different Quantification Approaches and Matrix Effect Studies. *Anal Bioanal Chem* **2015**, *407*, 821–830, doi:10.1007/s00216-014-8068-1.
114. Lonappan, L.; Pulicharla, R.; Rouissi, T.; Brar, S.K.; Verma, M.; Surampalli, R.Y.; Valero, J.R. Diclofenac in Municipal Wastewater Treatment Plant: Quantification Using Laser Diode Thermal Desorption--Atmospheric Pressure Chemical Ionization--Tandem Mass Spectrometry Approach in Comparison with an Established Liquid Chromatography-Electrospray Ionization-Tandem Mass Spectrometry Method. *J Chromatogr A* **2016**, *1433*, 106–113, doi:10.1016/j.chroma.2016.01.030.
115. Langford, K.H.; Reid, M.; Thomas, K.V. Multi-Residue Screening of Prioritised Human Pharmaceuticals, Illicit Drugs and Bactericides in Sediments and Sludge. *J Environ Monit* **2011**, *13*, 2284–2291, doi:10.1039/c1em10260e.
116. Mailler, R.; Gasperi, J.; Patureau, D.; Vulliet, E.; Delgenes, N.; Danel, A.; Deshayes, S.; Eudes, V.; Guerin, S.; Moilleron, R.; et al. Fate of Emerging and Priority Micropollutants during the Sewage Sludge Treatment: Case Study of Paris Conurbation. Part 1: Contamination of the Different Types of Sewage Sludge. *Waste Management* **2017**, *59*, 379–393, doi:10.1016/j.wasman.2016.11.010.
117. Shukla, R.; Ahammad, S.Z. Performance Assessment of a Modified Trickling Filter and Conventional Activated Sludge Process along with Tertiary Treatment in Removing Emerging Pollutants from Urban Sewage. *Science of The Total Environment* **2023**, *858*, 159833, doi:10.1016/j.scitotenv.2022.159833.
118. Llop, A.; Borrull, F.; Pocurull, E. Pressurised Hot Water Extraction Followed by Simultaneous Derivatization and Headspace Solid-Phase Microextraction and Gas Chromatography-Tandem Mass Spectrometry for the Determination of Aliphatic Primary Amines in Sewage Sludge. *Anal. Chim. Acta* **2010**, *665*, 231–236, doi:10.1016/j.aca.2010.03.042.
119. Llop, A.; Borrull, F.; Pocurull, E. Pressurised Hot Water Extraction Followed by Headspace Solid-Phase Microextraction and Gas Chromatography-Tandem Mass Spectrometry for the Determination of N-Nitrosamines in Sewage Sludge. *Talanta* **2012**, *88*, 284–289, doi:10.1016/j.talanta.2011.10.042.
120. Saleh, A.; Larsson, E.; Yamini, Y.; Jönsson, J.Å. Hollow Fiber Liquid Phase Microextraction as a Preconcentration and Clean-up Step after Pressurized Hot Water Extraction for the Determination of

Non-Steroidal Anti-Inflammatory Drugs in Sewage Sludge. *J Chromatogr A* **2011**, *1218*, 1331–1339, doi:10.1016/j.chroma.2011.01.011.
121. Herrero, P.; Borrull, F.; Pocurull, E.; Marcé, R.M. A Quick, Easy, Cheap, Effective, Rugged and Safe Extraction Method Followed by Liquid Chromatography-(Orbitrap) High Resolution Mass Spectrometry to Determine Benzotriazole, Benzothiazole and Benzenesulfonamide Derivates in Sewage Sludge. *Journal of Chromatography A* **2014**, *1339*, 34–41, doi:10.1016/j.chroma.2014.02.081.
122. Svahn, O.; Björklund, E. Extraction Efficiency of a Commercial Espresso Machine Compared to a Stainless-Steel Column Pressurized Hot Water Extraction (PHWE) System for the Determination of 23 Pharmaceuticals, Antibiotics and Hormones in Sewage Sludge. *Applied Sciences* **2019**, *9*, 1509, doi:10.3390/app9071509.
123. Rede, D.; Teixeira, I.; Delerue-Matos, C.; Fernandes, V.C. Assessing Emerging and Priority Micropollutants in Sewage Sludge: Environmental Insights and Analytical Approaches. *Environ Sci Pollut Res* **2024**, *31*, 3152–3168, doi:10.1007/s11356-023-30963-1.
124. Ramos, S.; Homem, V.; Santos, L. Development and Optimization of a QuEChERS-GC–MS/MS Methodology to Analyse Ultraviolet-Filters and Synthetic Musks in Sewage Sludge. *Science of The Total Environment* **2019**, *651*, 2606–2614, doi:10.1016/j.scitotenv.2018.10.143.
125. Peysson, W.; Vulliet, E. Determination of 136 Pharmaceuticals and Hormones in Sewage Sludge Using Quick, Easy, Cheap, Effective, Rugged and Safe Extraction Followed by Analysis with Liquid Chromatography–Time-of-Flight-Mass Spectrometry. *Journal of Chromatography A* **2013**, *1290*, 46–61, doi:10.1016/j.chroma.2013.03.057.
126. Rossini, D.; Ciofi, L.; Ancillotti, C.; Checchini, L.; Bruzzoniti, M.C.; Rivoira, L.; Fibbi, D.; Orlandini, S.; Del Bubba, M. Innovative Combination of QuEChERS Extraction with On-Line Solid-Phase Extract Purification and Pre-Concentration, Followed by Liquid Chromatography-Tandem Mass Spectrometry for the Determination of Non-Steroidal Anti-Inflammatory Drugs and Their Metabolites in Sewage Sludge. *Analytica Chimica Acta* **2016**, *935*, 269–281, doi:10.1016/j.aca.2016.06.023.
127. Benedetti, B.; Majone, M.; Cavaliere, C.; Montone, C.M.; Fatone, F.; Frison, N.; Laganà, A.; Capriotti, A.L. Determination of Multi-Class Emerging Contaminants in Sludge and Recovery Materials from Waste Water Treatment Plants: Development of a Modified QuEChERS Method Coupled to LC–MS/MS. *Microchemical Journal* **2020**, *155*, 104732, doi:10.1016/j.microc.2020.104732.
128. Miserli, K.; Kosma, C.; Konstantinou, I. Determination of Pharmaceuticals and Metabolites in Sludge and Hydrochar after Hydrothermal Carbonization Using Sonication—QuEChERS Extraction Method and UHPLC LTQ/Orbitrap MS. *Environ Sci Pollut Res* **2023**, *30*, 1686–1703, doi:10.1007/s11356-022-22215-5.
129. Angeles-de Paz, G.; Ledezma-Villanueva, A.; Robledo-Mahón, T.; Pozo, C.; Calvo, C.; Aranda, E.; Purswani, J. Assembled Mixed Co-Cultures for Emerging Pollutant Removal Using Native Microorganisms from Sewage Sludge. *Chemosphere* **2023**, *313*, 137472, doi:10.1016/j.chemosphere.2022.137472.
130. Ajibola, A.S.; Tisler, S.; Zwiener, C. Simultaneous Determination of Multiclass Antibiotics in Sewage Sludge Based on QuEChERS Extraction and Liquid Chromatography-Tandem Mass Spectrometry. *Anal. Methods* **2020**, *12*, 576–586, doi:10.1039/C9AY02188D.
131. Cerqueira, M.B.R.; Caldas, S.S.; Primel, E.G. New Sorbent in the Dispersive Solid Phase Extraction Step of Quick, Easy, Cheap, Effective, Rugged, and Safe for the Extraction of Organic Contaminants in Drinking Water Treatment Sludge. *Journal of Chromatography A* **2014**, *1336*, 10–22, doi:10.1016/j.chroma.2014.02.002.
132. Cerqueira, M.B.R.; Guilherme, J.R.; Caldas, S.S.; Martins, M.L.; Zanella, R.; Primel, E.G. Evaluation of the QuEChERS Method for the Extraction of Pharmaceuticals and Personal Care Products from Drinking-Water Treatment Sludge with Determination by UPLC-ESI-MS/MS. *Chemosphere* **2014**, *107*, 74–82, doi:10.1016/j.chemosphere.2014.03.026.
133. Bergé, A.; Buleté, A.; Fildier, A.; Vulliet, E. High-Resolution Mass Spectrometry as a Tool To Evaluate the Sample Preparation of Sludge. *Anal. Chem.* **2017**, *89*, 9685–9694, doi:10.1021/acs.analchem.7b01081.
134. Celano, R.; Rodríguez, I.; Cela, R.; Rastrelli, L.; Piccinelli, A.L. Liquid Chromatography Quadrupole Time-of-Flight Mass Spectrometry Quantification and Screening of Organophosphate Compounds in Sludge. *Talanta* **2014**, *118*, 312–320, doi:10.1016/j.talanta.2013.10.024.
135. González-Mariño, I.; Rodríguez, I.; Quintana, J.B.; Cela, R. Matrix Solid-Phase Dispersion Followed by Gas Chromatography-Mass Spectrometry for the Determination of Triclosan and Methyl Triclosan in Sludge and Sediments. *Anal Bioanal Chem* **2010**, *398*, 2289–2297, doi:10.1007/s00216-010-4136-3.

136. Sánchez-Brunete, C.; Miguel, E.; Albero, B.; Tadeo, J.L. Determination of Triclosan and Methyl Triclosan in Environmental Solid Samples by Matrix Solid-Phase Dispersion and Gas Chromatography-Mass Spectrometry. *J Sep Sci* **2010**, *33*, 2768–2775, doi:10.1002/jssc.201000284.
137. Albero, B.; Pérez, R.A.; Sánchez-Brunete, C.; Tadeo, J.L. Occurrence and Analysis of Parabens in Municipal Sewage Sludge from Wastewater Treatment Plants in Madrid (Spain). *Journal of Hazardous Materials* **2012**, *239–240*, 48–55, doi:10.1016/j.jhazmat.2012.05.017.
138. Casado, J.; Rodríguez, I.; Carpinteiro, I.; Ramil, M.; Cela, R. Gas Chromatography Quadrupole Time-of-Flight Mass Spectrometry Determination of Benzotriazole Ultraviolet Stabilizers in Sludge Samples. *Journal of Chromatography A* **2013**, *1293*, 126–132, doi:10.1016/j.chroma.2013.03.050.
139. Casado, J.; Castro, G.; Rodríguez, I.; Ramil, M.; Cela, R. Selective Extraction of Antimycotic Drugs from Sludge Samples Using Matrix Solid-Phase Dispersion Followed by on-Line Clean-Up. *Anal Bioanal Chem* **2015**, *407*, 907–917, doi:10.1007/s00216-014-8167-z.
140. Montes, R.; Rodríguez, I.; Casado, J.; López-Sabater, M.C.; Cela, R. Determination of the Cardiac Drug Amiodarone and Its N-Desethyl Metabolite in Sludge Samples. *Journal of Chromatography A* **2015**, *1394*, 62–70, doi:10.1016/j.chroma.2015.03.024.
141. Triñanes, S.; Casais, M.C.; Mejuto, M.C.; Cela, R. Matrix Solid-Phase Dispersion Followed by Liquid Chromatography Tandem Mass Spectrometry for the Determination of Selective Ciclooxygenase-2 Inhibitors in Sewage Sludge Samples. *Journal of Chromatography A* **2016**, *1462*, 35–43, doi:10.1016/j.chroma.2016.07.044.
142. Castro, G.; Carpinteiro, I.; Rodríguez, I.; Cela, R. Determination of Cardiovascular Drugs in Sewage Sludge by Matrix Solid-Phase Dispersion and Ultra-Performance Liquid Chromatography Tandem Mass Spectrometry. *Anal Bioanal Chem* **2018**, *410*, 6807–6817, doi:10.1007/s00216-018-1268-3.
143. Li, M.; Sun, Q.; Li, Y.; Lv, M.; Lin, L.; Wu, Y.; Ashfaq, M.; Yu, C. Simultaneous Analysis of 45 Pharmaceuticals and Personal Care Products in Sludge by Matrix Solid-Phase Dispersion and Liquid Chromatography Tandem Mass Spectrometry. *Anal Bioanal Chem* **2016**, *408*, 4953–4964, doi:10.1007/s00216-016-9590-0.
144. Cerqueira, M.B.R.; Soares, K.L.; Caldas, S.S.; Primel, E.G. Sample as Solid Support in MSPD: A New Possibility for Determination of Pharmaceuticals, Personal Care and Degradation Products in Sewage Sludge. *Chemosphere* **2018**, *211*, 875–883, doi:10.1016/j.chemosphere.2018.07.165.
145. Castro, G.; Ramil, M.; Cela, R.; Rodríguez, I. Identification and Determination of Emerging Pollutants in Sewage Sludge Driven by UPLC-QTOF-MS Data Mining. *Science of The Total Environment* **2021**, *778*, 146256, doi:https://doi.org/10.1016/j.scitotenv.2021.146256.
146. Andrade, V.F.; Durães, A.F.S.; Cassimiro, D.L.; Pinho, G.P. de; Silvério, F.O. Fast Extraction of Polychlorinated Dibenzo-p-Dioxin and Polychlorinated Dibenzofuran in Sewage Sludge and Soil Samples. *Journal of Environmental Science and Health, Part B* **2017**, *52*, 267–273, doi:10.1080/03601234.2016.1273003.
147. Maia, M.R.; Arcanjo, A.L.P.; Pinho, G.P.; Silvério, F.O.; Maia, M.R.; Arcanjo, A.L.P.; Pinho, G.P.; Silvério, F.O. Solid-Liquid Extraction with Low Temperature Purification Coupled with Gas Chromatography and Mass Spectrometry for Determination of Polychlorinated Biphenyls in Sewage Sludge. *Journal of the Brazilian Chemical Society* **2017**, *28*, 179–186, doi:10.5935/0103-5053.20160161.
148. Wu, S.-F.; Ding, W.-H. Fast Determination of Synthetic Polycyclic Musks in Sewage Sludge and Sediments by Microwave-Assisted Headspace Solid-Phase Microextraction and Gas Chromatography-Mass Spectrometry. *J Chromatogr A* **2010**, *1217*, 2776–2781, doi:10.1016/j.chroma.2010.02.067.
149. Vallecillos, L.; Pocurull, E.; Borrull, F. A Simple and Automated Method to Determine Macrocyclic Musk Fragrances in Sewage Sludge Samples by Headspace Solid-Phase Microextraction and Gas Chromatography–Mass Spectrometry. *Journal of Chromatography A* **2013**, *1314*, 38–43, doi:10.1016/j.chroma.2013.09.033.
150. Pereira, N.G.F.; Silvério, F.O.; Pinho, G.P. Optimisation, Validation and Application of the Solid-Liquid Extraction with Low-Temperature Purification Followed by Gas Chromatography-Mass Spectrometry for Determination of Phthalates in Sewage Sludge. *International Journal of Environmental Analytical Chemistry* **2020**, *100*, 968–980, doi:10.1080/03067319.2019.1646735.
151. Sagristà, E.; Larsson, E.; Ezoddin, M.; Hidalgo, M.; Salvadó, V.; Jönsson, J.Å. Determination of Non-Steroidal Anti-Inflammatory Drugs in Sewage Sludge by Direct Hollow Fiber Supported Liquid Membrane Extraction and Liquid Chromatography–Mass Spectrometry. *Journal of Chromatography A* **2010**, *1217*, 6153–6158, doi:10.1016/j.chroma.2010.08.005.
152. Larsson, E.; Rabayah, A. Sludge Removal of Nonsteroidal Anti-Inflammatory Drugs during Wastewater Treatment Studied by Direct Hollow Fiber Liquid Phase Microextraction. **2013**.
153. Manso, J.; Larsson, E.; Jönsson, J.Å. Determination of 4′-Isobutylacetophenone and Other

Transformation Products of Anti-Inflammatory Drugs in Water and Sludge from Five Wastewater Treatment Plants in Sweden by Hollow Fiber Liquid Phase Microextraction and Gas Chromatography–Mass Spectrometry. *Talanta* **2014**, *125*, 87–93, doi:10.1016/j.talanta.2014.02.056.
154. Ferreira, A.M.C.; Möder, M.; Laespada, M.E.F. Stir Bar Sorptive Extraction of Parabens, Triclosan and Methyl Triclosan from Soil, Sediment and Sludge with in Situ Derivatization and Determination by Gas Chromatography–Mass Spectrometry. *Journal of Chromatography A* **2011**, *1218*, 3837–3844, doi:10.1016/j.chroma.2011.04.055.
